# ﻿Catalog of the invertebrate type specimens hosted at the Pontificia Universidad Católica del Ecuador and Escuela Politécnica Nacional natural history collections

**DOI:** 10.3897/zookeys.1169.102030

**Published:** 2023-07-05

**Authors:** Fernanda Salazar-Buenaño, Diego Guevara, Alvaro Barragán, Vladimir Carvajal, David A. Donoso

**Affiliations:** 1 Museo de Zoología, Escuela de Ciencias Biológicas, Pontificia Universidad Católica del Ecuador, Quito, Ecuador Pontificia Universidad Católica del Ecuador Quito Ecuador; 2 Museo de Historia Natural Gustavo Orcés, Escuela Politécnica Nacional, Quito, Ecuador Museo de Historia Natural Gustavo Orcés, Escuela Politécnica Nacional Quito Ecuador

**Keywords:** Arthropods, biodiversity, Cajanuma, conservation, insects, Otonga, type localities

## Abstract

This work updates the invertebrate type specimen catalog published by Donoso et al. (2009). The catalog is increased by 2281 type specimens (from 454 species or subspecies) to a total of 4180 type specimens (from 770 species or subspecies) hosted at the Pontificia Universidad Católica del Ecuador and Escuela Politécnica Nacional natural history collections. The new material adds 307 holotypes, 1910 paratypes, and 64 allotypes. It provides original information from four phyla (Arthropoda, Mollusca, Nemata, and Platyhelminthes), eight classes, 21 orders, 73 families, and 156 genera. This updated catalog includes a map showing the type localities in the country, a list of the 71 new type specimens (from 23 species or subspecies) from other countries hosted at both museums, corrections to the previous catalog published by Donoso et al. (2009), and label information from each new specimen.

## ﻿Introduction

In 2009, we published the first catalog of invertebrate type specimens in the collection at Pontificia Universidad Católica del Ecuador (QCAZI), the largest in Ecuador (Donoso et al. 2009). The catalog provided information on type specimens in the collection up to the year 2008 and explored general patterns of collection biases associated with this type material. Donoso et al. (2009) found that invertebrate type material was associated with roads near Quito (i.e., the country’s capital city with the major international airport). Furthermore, these type specimens were biased towards a few localities not incorporated in the national system of protected areas. Since then, several hundred specimens have accumulated in the QCAZI collection.

The museum at Escuela Politécnica Nacional (MEPN), in Quito, Ecuador, was created in 1946 by the naturalist Gustavo Orcés and manages invertebrate, mammal, amphibian, reptile, and fish collections (Carrera et al. 2020). After QCAZI, the MEPN museum likely holds the second most important invertebrate collection in the country. The MEPN invertebrate collection preserves more than 10 million invertebrates in collection jars. It is well represented by canopy fogging and aquatic invertebrate samples but there is no catalog. Thus, an analysis of the updated QCAZI and the new MEPN catalogs provide us with important insights into the development of the study of invertebrates in Ecuador in the last decade.

## ﻿Materials and methods

In this work, we update the 2008 dataset to include the new type specimens deposited at the Pontificia Universidad Católica del Ecuador **(QCAZI)** museum after 2008 and up to 2020 and, for the first-time, list the type specimens stored in the Museum at Escuela Politécnica Nacional **(MEPN)**, in Quito. Type specimens from other countries hosted in both museums have also been included. Errors of type designation that were included in the 2008 dataset have been excluded from the list in this update. Finally, type names misspelled in the 2008 dataset have been corrected.

We compiled specimens for this list by gathering all recent entries of type specimens: holotype, paratypes, and allotypes (i.e., paratypes of the sex opposite to that of the holotype) in the QCAZI and MEPN collections. We also searched all cabinets at both museums for invertebrate type specimens. Additionally, we searched for invertebrate type specimens in the primary literature using online search engines. Coordinates to the localities were extracted; localities with no coordinates or with incorrect information were georeferenced using the Google Earth Engine and the locality database from the QCAZI Museum hosted at Bioweb (Pontificia Universidad Católica del Ecuador 2021).

## ﻿Results

**Figure 1.**
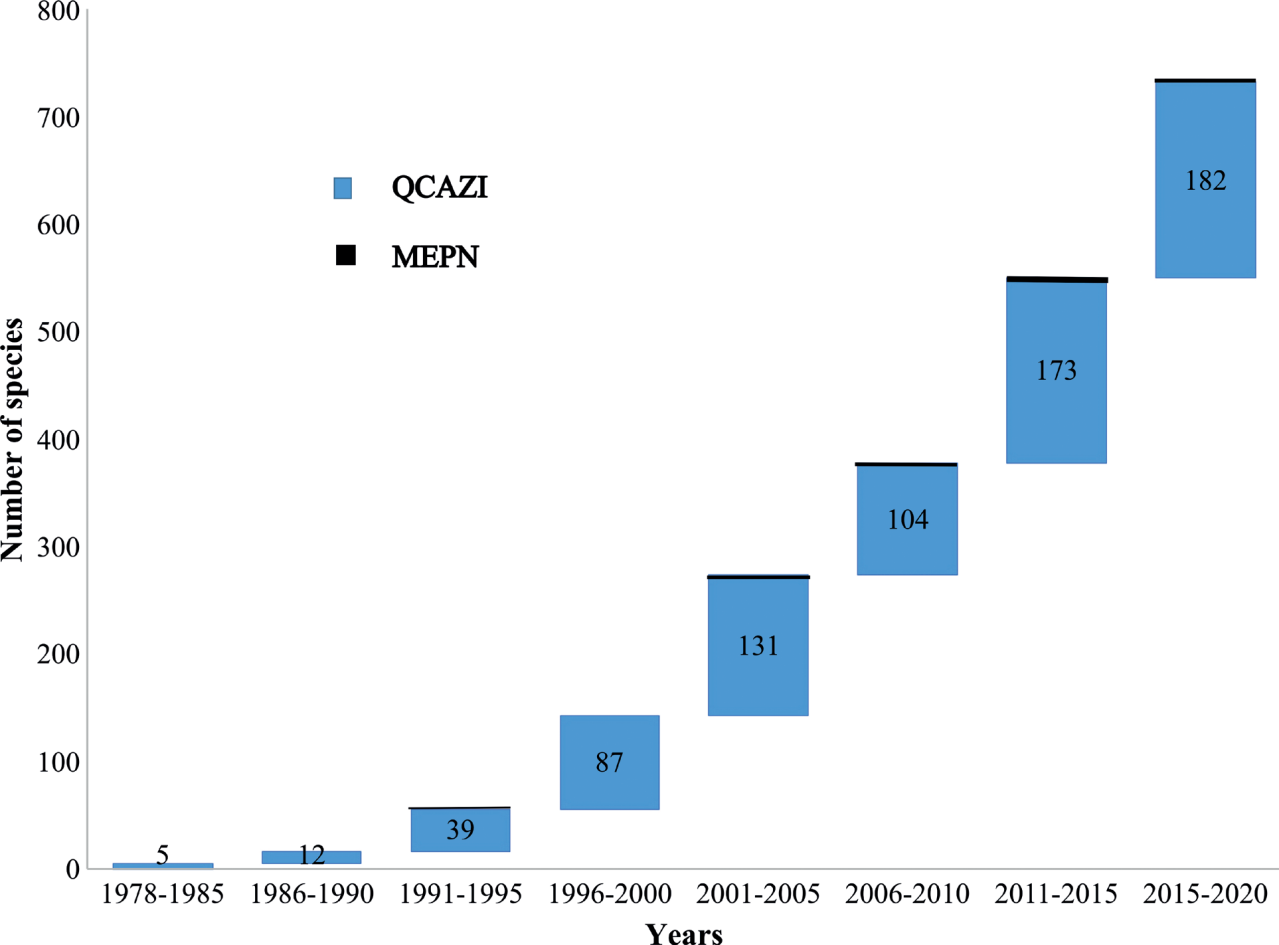
Cumulative number of Ecuadorian species with types in the QCAZI (blue) and MEPN (black) museums. The size and number inside the box correspond to the number of species lodged in both museums in that time period.

The new list of type material includes 2281 type specimens from 307 holotypes, 1910 paratypes, and 64 allotypes (Table 1). The specimens belong to four phyla, eight classes, 21 orders, 73 families, 156 genera, and 454 species or subspecies. A species accumulation curve of the complete datasets (2008 and 2020) shows a continuing increase in the number of Ecuadorian species described since 1978 (Fig. 1); for example, in the last five years, type specimens of 182 new invertebrate species were deposited at these two museums. We provide verbatim label information for all new type material in the Suppl. material 1.

**Table 1.** New type specimens of Ecuadorian species deposited at the QCAZI and MEPN museums. The species are organized by phylum, class, order, family, genus, species, and authority. All the species are preserved at the QCAZI except those with the acronym MEPN with type status as follows: H = holotype, P = paratype, and A = allotype; the bibliographic reference where the type was designated is in parentheses.

**Phylum Arthropoda**
**Class Arachnida**
**Order Araneae**
**Family Anapidae**
*Anapisanabelleae* Dupérré & Tapia, 2018; H, P; (Dupérré and Tapia 2018)
*Anapiscarmencita* Dupérré & Tapia, 2018; H; (Dupérré and Tapia 2018)
*Anapischuru* Dupérré & Tapia, 2018; H, P; (Dupérré and Tapia 2018)
*Anapismariebertheae* Dupérré & Tapia, 2018; H, P; (Dupérré and Tapia 2018)
*Anapisnaranja* Dupérré & Tapia, 2018; H, P; (Dupérré and Tapia 2018)
*Anapisnawchi* Dupérré & Tapia, 2018; H, P; (Dupérré and Tapia 2018)
*Anapisshina* Dupérré & Tapia, 2018; H, P; (Dupérré and Tapia 2018)
**Family Anyphaenidae**
*Katissaguayasamini* Dupérré & Tapia, 2016; H; (Dupérré and Tapia 2016)
*Katissakurusiki* Dupérré & Tapia, 2016; H; P; (Dupérré and Tapia 2016)
*Katissapuyu* Dupérré & Tapia, 2016; H; P; (Dupérré and Tapia 2016)
*Katissatamya* Dupérré & Tapia, 2016; H; (Dupérré and Tapia 2016)
*Katissayaya* Dupérré & Tapia, 2016; H; (Dupérré and Tapia 2016)
*Patrerahatunkiru* Dupérré & Tapia, 2016; H; (Dupérré and Tapia 2016)
*Patreraphilipi* Dupérré & Tapia, 2016; H; (Dupérré and Tapia 2016)
*Patrerashida* Dupérré & Tapia, 2016; H; (Dupérré and Tapia 2016)
*Patrerasuni* Dupérré & Tapia, 2016; H; (Dupérré and Tapia 2016)
*Patrerawitsu* Dupérré & Tapia, 2016; H; (Dupérré and Tapia 2016)
*Shuyushkaachachay* Dupérré & Tapia, 2016; H; (Dupérré and Tapia 2016)
*Shuyushkamoscai* Dupérré & Tapia, 2016; H; (Dupérré and Tapia 2016)
*Shuyushkawachi* Dupérré & Tapia, 2016; H; (Dupérré and Tapia 2016)
**Family Caponiidae**
*Nopscesari* Dupérré, 2014; H, P; (Dupérré 2014)
*Nopsquito* Dupérré, 2014; H; (Dupérré 2014)
*Nyetnopsjuchuy* Dupérré, 2014; H; (Dupérré 2014)
**Family Ctenidae**
*Chococtenuscappuccino* Dupérré, 2015; H, P; (Dupérré 2015a)
*Chococtenuscuchilla* Dupérré, 2015; H, P; (Dupérré 2015a)
*Chococtenusduendecito* Dupérré, 2015; H; (Dupérré 2015a)
*Chococtenusfantasma* Dupérré, 2015; H, P; (Dupérré 2015a)
*Chococtenuskashkara* Dupérré, 2015; H, P; (Dupérré 2015a)
*Chococtenuslasdamas* Dupérré, 2015; H, P; (Dupérré 2015a)
*Chococtenusluchoi* Dupérré, 2015; H; (Dupérré 2015a)
*Chococtenusneblina* Dupérré, 2015; H, P; (Dupérré 2015a)
*Chococtenusotonga* Dupérré, 2015; H, P; (Dupérré 2015a)
*Chococtenusotongachi* Dupérré, 2015; H, P; (Dupérré 2015a)
*Chococtenuspiemontana* Dupérré, 2015; H; (Dupérré 2015a)
*Chococtenussuffuscus* Dupérré, 2015; H, P; (Dupérré 2015a)
*Chococtenuswaitti* Dupérré, 2015; H; (Dupérré 2015a)
**Family Dipluridae**
*Linothelepukachumpi* Dupérré & Tapia, 2015; H, P; (Dupérré and Tapia 2015a)
*Linothelequori* Dupérré & Tapia, 2015; H, P; (Dupérré and Tapia 2015a)
*Linotheletsachilas* Dupérré & Tapia, 2015; H, P; (Dupérré and Tapia 2015a)
*Linotheleyanachanka* Dupérré & Tapia, 2015; H, P; (Dupérré and Tapia 2015a)
*Linothelezaia* Dupérré & Tapia, 2015; H; (Dupérré and Tapia 2015a)
**Family Mysmenidae**
*Mysmenopsisalvaroi* Dupérré & Tapia, 2020; H, A, P; (Dupérré and Tapia 2020a)
*Mysmenopsisamazonica* Dupérré & Tapia, 2020; H, P; (Dupérré and Tapia 2020a)
*Mysmenopsisangamarca* Dupérré & Tapia, 2020; H, P; (Dupérré and Tapia 2020a)
*Mysmenopsisawa* Dupérré & Tapia, 2020; H, A; (Dupérré and Tapia 2020a)
*Mysmenopsisbaerti* Dupérré & Tapia, 2020; H, A, P; (Dupérré and Tapia 2020a)
*Mysmenopsisbartolozzii* Dupérré & Tapia, 2020; H, A, P; (Dupérré and Tapia 2020a)
*Mysmenopsischiquita* Dupérré & Tapia, 2015; H, P; (Dupérré and Tapia 2015a)
*Mysmenopsischoco* Dupérré & Tapia, 2020; H, A, P; (Dupérré and Tapia 2020a)
*Mysmenopsiscorazon* Dupérré & Tapia, 2020; H; (Dupérré and Tapia 2020a)
*Mysmenopsiscube* Dupérré & Tapia, 2020; H, A, P; (Dupérré and Tapia 2020a)
*Mysmenopsisfernandoi* Dupérré & Tapia, 2015; H, P; (Dupérré and Tapia 2015a)
*Mysmenopsisguanza* Dupérré & Tapia, 2020; H, A, P; (Dupérré and Tapia 2020a)
*Mysmenopsisguayaca* Dupérré & Tapia, 2020; H, A, P; (Dupérré and Tapia 2020a)
*Mysmenopsishunachi* Dupérré & Tapia, 2020; H; (Dupérré and Tapia 2020a)
*Mysmenopsisjunin* Dupérré & Tapia, 2020; H, A, P; (Dupérré and Tapia 2020a)
*Mysmenopsislasrocas* Dupérré & Tapia, 2020; H, A; (Dupérré and Tapia 2020a)
*Mysmenopsislloa* Dupérré & Tapia, 2020; H; (Dupérré and Tapia 2020a)
*Mysmenopsisonorei* Dupérré & Tapia, 2015; H, P; (Dupérré and Tapia 2015a)
*Mysmenopsisotokiki* Dupérré & Tapia, 2020; H, A, P; (Dupérré and Tapia 2020a)
*Mysmenopsisotonga* Dupérré & Tapia, 2015; H, P; (Dupérré and Tapia 2015a)
*Mysmenopsispululahua* Dupérré & Tapia, 2020; H, A, P; (Dupérré and Tapia 2020a)
*Mysmenopsissalazarae* Dupérré & Tapia, 2020; H, A, P; (Dupérré and Tapia 2020a)
*Mysmenopsisshushufindi* Dupérré & Tapia, 2020; H, A, P; (Dupérré and Tapia 2020a)
*Mysmenopsistepuy* Dupérré & Tapia, 2020; H; (Dupérré and Tapia 2020a)
*Mysmenopsistungurahua* Dupérré & Tapia, 2020; H; (Dupérré and Tapia 2020a)
**Family Ochyroceratidae**
*Ochyroceracallaina* Dupérré, 2015; H, P; (Dupérré 2015c)
*Ochyroceracashcatotoras* Dupérré, 2015; H; (Dupérré 2015c)
Ochyrocera*italoi* Dupérré, 2015; H; (Dupérré 2015c)
*Ochyroceralosrios* Dupérré, 2015; H; (Dupérré 2015c)
*Ochyroceraminotaure* Dupérré, 2015; H; (Dupérré 2015c)
*Ochyroceraotonga* Dupérré, 2015; H; (Dupérré 2015c)
*Ochyrocerarinocerotos* Dupérré, 2015; H, P; (Dupérré 2015c)
*Ochyrocerazabaleta* Dupérré, 2015; H, P; (Dupérré 2015c)
*Psiloochyroceratortilis* Dupérré, 2015; H; (Dupérré 2015c)
*Speocerabioforestae* Dupérré, 2015; H, P; (Dupérré 2015c)
*Speoceramusgo* Dupérré, 2015; H, P; (Dupérré 2015c)
*Speoceraviolacea* Dupérré, 2015; H; (Dupérré 2015c)
**Family Oonopidae**
*Bipoonopslansa* Dupérré & Tapia, 2017; H; (Dupérré and Tapia 2017a)
*Bipoonopspilan* Dupérré & Tapia, 2017; H; (Dupérré and Tapia 2017a)
*Neotropsplatnicki* Grismado & Ramírez, 2013; H; (Grismado and Ramírez 2013)
*Niarchosnormani* Dupérré & Tapia, 2017; H; (Dupérré and Tapia 2017a)
*Reductoonopsberun* Dupérré & Tapia, 2017; H, A, P; (Dupérré and Tapia 2017a)
*Scaphidysderinachirin* Dupérré & Tapia, 2017; H, A; (Dupérré and Tapia 2017a)
*Scaphidysderinalubanako* Dupérré & Tapia, 2017; H, A; (Dupérré and Tapia 2017a)
*Scaphidysderinatsaran* Dupérré & Tapia, 2017; H, A; (Dupérré and Tapia 2017a)
**Family Paratropididae**
*Paratropiselicioi* Dupérré, 2015; H, P; (Dupérré 2015b)
*Paratropisotonga* Dupérré & Tapia, 2020; H, P; (Dupérré and Tapia 2020b)
*Paratropispristirana* Dupérré & Tapia, 2020; H, A, P; (Dupérré and Tapia 2020b)
**Family Sparassidae**
*Anaptomecusparu* Guala, Labarque & Rheims, 2012; H; (Guala et al. 2012)
**Family Symphytognathidae**
*Anapistulaequatoriana* Dupérré & Tapia, 2017; H, P; (Dupérré and Tapia 2017b)
*Symphytognathacabezota* Dupérré & Tapia, 2017; H, P; (Dupérré and Tapia 2017b)
**Family Telemidae**
*Kinkuturumanya* Dupérré & Tapia, 2015; H, P; (Dupérré and Tapia 2015b)
**Family Theridiosomatidae**
*Chthonoskuyllur* Dupérré & Tapia, 2017; H, P; (Dupérré and Tapia 2017b)
*Naatlomayzana* Dupérré & Tapia, 2017; H; (Dupérré and Tapia 2017b)
*Ogulniuslaranka* Dupérré & Tapia, 2017; H, A; (Dupérré and Tapia 2017b)
*Ogulniuspaku* Dupérré & Tapia, 2017; H, P; (Dupérré and Tapia 2017b)
*Theridiosomaankas* Dupérré & Tapia, 2017; H; (Dupérré and Tapia 2017b)
*Theridiosomaesmeraldas* Dupérré & Tapia, 2017; H; (Dupérré and Tapia 2017b)
*Theridiosomakullki* Dupérré & Tapia, 2017; H, P; (Dupérré and Tapia 2017b)
*Theridiosomasacha* Dupérré & Tapia, 2017; H, P; (Dupérré and Tapia 2017b)
**Order Opiliones**
**Family Neogoveidae**
*Metagovealigiae* Giupponi & Kury, 2015; H, P; (Giupponi and Kury 2015)
**Family Cranaidae**
*Zannicranausmonoclonius* Kury, 2012; H, A; (Kury 2012)
*Zannicranausmorlaucus* Kury, 2012; H; (Kury 2012)
**Order Pseudoscorpiones**
**Family Withiidae**
*Cystowithiussmithersi* Harvey, 2004; P; (Harvey 2004)
**Order Ricinulei**
**Family Ricinoididae**
*Cryptocelluschiruisla* Botero & Flórez, 2017; H, P; (Botero and Flórez 2017)
**Order Sarcoptiformes**
**Family Lohmanniidae**
*Lohmanniavulcania* Schatz, 1993; P; (Schatz 1993)
*Torpacarusremotus* Schatz, 1994; P; (Schatz 1994)
**Order Schizomida**
**Family Hubbardiidae**
*Surazomuskitu* Villarreal, Silva & Giupponi, 2016; H; (Villarreal et al. 2016)
*Surazomuspalenque* Villarreal, Silva & Giupponi, 2016; H, P; (Villarreal et al. 2016)
**Order Scorpiones**
**Family Chactidae**
*Teuthrausteskuryi* Ythier & Lourenço, 2017; H; (Ythier and Lourenço 2017)
**Class Chilopoda**
**Order Geophilomorpha**
**Family Ballophilidae**
*Ityphilusgrismadoi* Pereira, 2018; H; (Pereira 2018b)
**Family Schendylidae**
*Pectiniunguisaequatorialis* Pereira, 2018; H; (Pereira 2018a)
**Class Copepoda**
**Order Siphonostomatoida**
**Family Caligidae**
*Pupulinamantensis* Cruz, Caña, Suárez & Santana, 2018; H, A, P; (Cruz et al. 2018)
**Class Diplopoda**
**Order Polydesmida**
**Family Platyrhacidae**
*Barydesmusnangaritza* Recuero & Sánchez, 2018; H; (Recuero and Sánchez 2018)
**Class Insecta**
**Order Coleoptera**
**Family Cantharidae**
*Maroniuspapallactae* Constantin, 2007; P; (Constantin 2007)
*Plectonotumcrassicorne* Constantin, 2008; P; (Constantin 2008a)
*Plectonotumglaber* Constantin, 2008; P; (Constantin 2008a)
*Plectonotumlatithorax* Constantin, 2008; P; (Constantin 2008a)
*Plectonotummacaraense* Constantin, 2010; P; (Constantin 2010)
*Plectonotummoreti* Constantin, 2008; P; (Constantin 2008a)
*Plectonotumnigricorne* Constantin, 2008; P; (Constantin 2008a)
*Plectonotumonorei* Constantin, 2008; P; (Constantin 2008a)
*Plectonotumpuncticollis* Constantin, 2008; P; (Constantin 2008a)
*Plectonotumzanjarajunoense* Constantin, 2010; P; (Constantin 2010)
*Silisbarragani* Constantin, 2010; H; (Constantin 2010)
*Siliselongatipennis* Constantin, 2009; P; (Constantin 2009)
*Silisgilletti* Constantin, 2009; P; (Constantin 2009).
*Silisotongae* Constantin, 2009; H, P; (Constantin 2009)
**Family Carabidae**
*Balligratusbrevis* Moret & Ortuño, 2017; P; (Moret and Ortuño 2017)
*Balligratushumerangulus* Moret & Ortuño, 2017; P; (Moret and Ortuño 2017)
*Bembidionricei* Maddison & Toledano, 2012; H; (Maddison and Toledano 2012)
*Blennidusamaluzanus* Moret, 2005; P; (Moret 2005)
*Calleidadesenderi* Casale, 2011; H; (Casale 2011)
*Chlaeniuswalterrossii* Giachino & Allegro, 2018; P; (Giachino and Allegro 2018)
*Diploharpuscurtulus* Moret, 2008; P; (Moret 2008)
*Dyscolusaquator* Moret & Murienne, 2020; H; (Moret and Murienne 2020)
*Dyscolusarauzae* Moret & Murienne, 2020; H; (Moret and Murienne 2020)
*Dyscolusbarragani* Moret & Murienne, 2020; H; (Moret and Murienne 2020)
*Dyscoluscrespoae* Moret & Murienne, 2020; H; (Moret and Murienne 2020)
*Dyscolusdonosoi* Moret & Murienne, 2020; H; (Moret and Murienne 2020)
*Dyscoluseleonorae* Moret & Murienne, 2020; H, P; (Moret and Murienne 2020)
*Dyscolusfamelicus* Moret & Murienne, 2020; H; (Moret and Murienne 2020)
*Dyscolusgiselae* Moret & Murienne, 2020; H, P; (Moret and Murienne 2020)
*Dyscolusgobbii* Moret & Murienne, 2020; H; (Moret and Murienne 2020)
*Dyscolusincommunis* Moret & Murienne, 2020; H; (Moret and Murienne 2020)
*Dyscolusmarini* Moret & Murienne, 2020; H, P; (Moret and Murienne 2020)
*Dyscoluspiscator* Moret & Murienne, 2020; H; (Moret and Murienne 2020)
*Dyscolusplacitus* Moret & Murienne, 2020; P; (Moret and Murienne 2020)
*Dyscolusravidus* Moret & Murienne, 2020; H, P; (Moret and Murienne 2020)
*Dyscolusrivinus* Moret & Murienne, 2020; H, P; (Moret and Murienne 2020)
*Dyscolusrugitarsis* Moret & Murienne, 2020; H, P; (Moret and Murienne 2020)
*Dyscolusruizi* Moret & Murienne, 2020; H, P; (Moret and Murienne 2020)
*Dyscolussilvestris* Moret & Murienne, 2020; H, P; (Moret and Murienne 2020)
*Dyscolussulcipedis* Moret & Murienne, 2020; H; (Moret and Murienne 2020)
*Dyscolusvelox* Moret, 2005; P; (Moret 2005).
*Hybopteratiputini* Erwin & Henry, 2017; P; MEPN; (Erwin and Henry 2017)
*Hybopteravestiverdis* Erwin & Henry, 2017; P; MEPN; (Erwin and Henry 2017)
*Loxandrussemperfidelis* Will, 2008; P; (Will 2008)
*Moriosomusloebli* Allegro, Giachino & Picciau, 2018; P; (Allegro et al. 2018)
*Tetrachaonorei*Naviaux 2007; A; (Naviaux 2007)
*Trechisibusbarragani* Deuve & Moret, 2017; H; (Deuve and Moret 2017)
*Trechisibusemiliae* Deuve & Moret, 2017; H; (Deuve and Moret 2017)
*Trechisibuspubescens* Deuve & Moret, 2017; H, P; (Deuve and Moret 2017)
**Family Chrysomelidae**
*Beltiaawapita* Flowers, 2018; H; (Flowers 2018)
*Beltialedesmae* Flowers, 2018; H, A, P; (Flowers 2018)
*Beltianapoensis* Flowers, 2018; P; (Flowers 2018)
*Beltiatalaga* Flowers, 2018; H; (Flowers 2018)
*Elytromenaconstantini* Daccordi, 2008; P; (Daccordi 2008)
**Family Curculionidae**
*Akrobothrusecuadoriensis* Dole & Cognato, 2007; H, A; MEPN; (Dole and Cognato 2007)
*Camptoceruslucwildi* Smith & Cognato, 2017; P; (Smith and Cognato 2017)
*Coptoborusochromactonus* Smith & Cognato, 2014; H, P; (Stilwell et al. 2014)
*Coptonotusuteq* Smith & Cognato, 2016; P; (Smith and Cognato 2016)
*Howdeniolamargheritae* Belló & Osella, 2008; P; (Belló and Osella 2008)
*Howdeniolaonorei* Belló & Osella, 2008; P; (Belló and Osella 2008)
**Family Elateridae**
*Paradrapetesserratus* Aranda, 1999; H; (Aranda 1999)
**Family Elmidae**
*Cylloepusbartolozzii* Monte & Mascagni, 2012; P; (Monte and Mascagni 2012)
*Cylloepuscesari* Monte & Mascagni, 2012; P; (Monte and Mascagni 2012)
*Cylloepusfabianorum* Monte & Mascagni, 2012; P; (Monte and Mascagni 2012)
*Cylloepusfrancescae* Monte & Mascagni, 2012; P; (Monte and Mascagni 2012)
*Cylloepusmazzai* Monte & Mascagni, 2012; P; (Monte and Mascagni 2012)
*Cylloepusterzanii* Monte & Mascagni, 2012; P; (Monte and Mascagni 2012)
*Macrelmiselicioi* Monte & Mascagni, 2012; P; (Monte and Mascagni 2012)
**Family Hybosoridae**
*Germarostesotonga* Ballerio & Gill, 2008; H, P; (Ballerio and Gill 2008)
**Family Leiodidae**
*Adelopsisazuay* Salgado, 2013; H, P; (Salgado 2013)
*Adelopsiscarolinae* Salgado, 2008; P; (Salgado 2008)
*Adelopspeleonacuminatum* Salgado, 2012; P; (Salgado 2012)
*Dissochaetusangustilis* Salgado, 2010; P; (Salgado 2010a)
*Eucatopstungurahuaensis* Salgado, 2010; H; (Salgado 2010b)
*Ptomaphaguscubensis* Salgado, 2012; H, P; (Salgado 2012)
**Family Lepiceridae**
*Lepiceruspichilingue* Flowers, Shepard & Troya, 2010; H, P; (Flowers et al. 2010)
**Family Lucanidae**
*Syndesusluki* Onore, Bartolozzi & Zilioli, 2011; P; (Onore et al. 2011)
**Family Melyridae**
*Astylusmoreti* Constantin, 2011; P; (Constantin 2011)
*Melyrodeslojaensis* Constantin, 2008; P; (Constantin 2008b)
**Family Nitidulidae**
*Pocadiusmaquipucunensis* Leschen & Carlton, 1994; P; (Leschen and Carlton 1994)
**Family Phengodidae**
*Pseudophengodesonorei* Wittmer, 1996; P; (Wittmer 1996)
**Family Scarabaeidae**
*Amithaocotopaxicus* Ratcliffe, 2017; P; (Ratcliffe 2017)
*Chrysinadzidorhum* (Arnaud, 1994); P; (Arnaud 1994)
*Cyclocephalaguaguarum* Dechambre & Endrödi, 1984; P; (Dechambre and Endrödi 1984)
*Cyclocephalaniguasa* Dechambre & Endrödi, 1984; P; (Dechambre and Endrödi 1984)
*Eurysternuscontractus* Génier, 2009; P; (Génier 2009)
*Eurysternuslanuginosus* Génier, 2009; P; (Génier 2009)
*Gymnetisdrogoni* Ratcliffe, 2018; P; (Ratcliffe 2018)
*Gymnetisviserioni* Ratcliffe, 2018; P; (Ratcliffe 2018)
*Odontolytestectipennis* (Stebnicka & Skelley, 2005); H, P; MEPN; (Stebnicka and Skelley 2005)
*Odontolyteswaoraniae* (Stebnicka & Skelley, 2005); H, P; MEPN (Stebnicka and Skelley 2005)
*Onoriusinexpectatus* Frolov & Vaz de Mello, 2015; H, P; (Frolov and Vaz de Mello 2015)
*Palaeophileurussilvestris* Neita & Ratcliffe, 2017; P; (Neita and Ratcliffe 2017)
*Phanaeusachilleslydiae* Arnaud, 2000; P; (Arnaud 2000)
*Spodochlamysnazareti* Arnaud, 1995; P; (Arnaud 1995)
**Family Staphylinidae**
*Gnathymenusrossii* Assing, 2013; P; (Assing 2013)
*Leptoniaonorei* Pace, 2008; P; (Pace 2008)
**Order Diptera**
**Family Anthomyzidae**
*Mumetopiamessor* Rohácek & Barber, 2008; P; (Rohácek and Barber 2008)
**Family Aulacigastridae**
*Aulacigasteralbifacies* Rung & Mathis, 2011; P; MEPN; (Rung and Mathis 2011)
*Aulacigasterformosa* Rung & Mathis, 2011; P; MEPN; (Rung and Mathis 2011)
*Aulacigastertrifasciata* Rung & Mathis, 2011; P; MEPN; (Rung and Mathis 2011)
*Aulacigasterunifasciata* Rung & Mathis, 2011; P; MEPN; (Rung and Mathis 2011)
*Aulacigastervespertina* Rung & Mathis, 2011; P; MEPN; (Rung and Mathis 2011)
**Family Ceratopogonidae**
*Forciponyiaaidae* Hochman, Marino & Spinelli, 2017; H, A; (Hochman et al. 2017)
*Forciponyiaivani* Hochman, Marino & Spinelli, 2017; H, A, P; (Hochman et al. 2017)
**Family Clusiidae**
*Craspedochaetaargoniae* Lonsdale & Marshall, 2006; P; (Lonsdale and Marshall 2006)
*Craspedochaetapollostos* Lonsdale & Marshall, 2006; P; (Lonsdale and Marshall 2006)
*Hendeliaheliconiae* Lonsdale & Marshall, 2011; P; (Lonsdale and Marshall 2011)
*Sobarocephalaarchisobarocephala* Lonsdale & Marshall, 2012; H; (Lonsdale and Marshall 2012)
*Sobarocephalabucki* Lonsdale & Marshall, 2012; H; (Lonsdale and Marshall 2012)
*Sobarocephalabulbosus* Lonsdale & Marshall, 2012; P; (Lonsdale and Marshall 2012)
*Sobarocephaladichotomos* Lonsdale & Marshall, 2012; P; (Lonsdale and Marshall 2012)
*Sobarocephalaechinata* Lonsdale & Marshall, 2012; P; (Lonsdale and Marshall 2012)
*Sobarocephalaepeira* Lonsdale & Marshall, 2012; P; (Lonsdale and Marshall 2012)
*Sobarocephalafuscina* Lonsdale & Marshall, 2012; P; (Lonsdale and Marshall 2012)
*Sobarocephalahispidifunda* Lonsdale & Marshall, 2012; P; (Lonsdale and Marshall 2012)
*Sobarocephalaleptolineata* Lonsdale & Marshall, 2012; P; (Lonsdale and Marshall 2012)
*Sobarocephalalita* Lonsdale & Marshall, 2012; P; (Lonsdale and Marshall 2012)
*Sobarocephalamaquipucuna* Lonsdale & Marshall, 2012; H; (Lonsdale and Marshall 2012)
*Sobarocephalapaieroi* Lonsdale & Marshall, 2012; H; (Lonsdale and Marshall 2012)
*Sobarocephalapatina* Lonsdale & Marshall, 2012; H; (Lonsdale and Marshall 2012)
*Sobarocephalapectinaria* Lonsdale & Marshall, 2012; P; (Lonsdale and Marshall 2012)
*Sobarocephalasinuata* Lonsdale & Marshall, 2012; P; (Lonsdale and Marshall 2012)
*Sobarocephalaspatulata* Lonsdale & Marshall, 2012; P; (Lonsdale and Marshall 2012)
*Sobarocephalasubtriangulina* Lonsdale & Marshall, 2012; H; (Lonsdale and Marshall 2012)
*Sobarocephalatinctoalata* Lonsdale & Marshall, 2012; H; (Lonsdale and Marshall 2012)
**Family Curtonotidae**
*Curtonotumbivittatum* Klymko & Marshall, 2011; H; (Klymko and Marshall 2011)
**Family Drosophilidae**
*Drosophilaanthurium* Llangarí & Rafael, 2020; H, P; (Llangarí and Rafael 2020)
*Drosophilaayauma* Peñafiel & Rafael, 2019; H, P; (Peñafiel and Rafael 2019a)
*Drosophilacajanuma* Peñafiel & Rafael, 2019; H, P; (Peñafiel and Rafael 2019b)
*Drosophilacarchensis* Peñafiel & Rafael, 2018; H, A, P; (Peñafiel and Rafael 2018c)
*Drosophilacartucho* Llangarí & Rafael, 2020; H, P; (Llangarí and Rafael 2020)
*Drosophilacarvalhoi* Cabezas, Llangarí & Rafael, 2015; H, A, P; (Cabezas et al. 2015)
*Drosophilacashapamba* Céspedes & Rafael, 2012; H, A, P; (Céspedes and Rafael 2012a)
*Drosophilacaxarumi* Peñafiel & Rafael, 2018; H, P; (Peñafiel and Rafael 2018b)
*Drosophilachichu* Peñafiel & Rafael, 2019; H, P; (Peñafiel and Rafael 2019a)
*Drosophilachorlavi* Céspedes & Rafael, 2012; H, A, P; (Céspedes and Rafael 2012a)
*Drosophilacondorhuachana* Céspedes & Rafael, 2012; H, P; (Céspedes and Rafael 2012b)
*Drosophilacosanga* Ramos & Rafael, 2017; H; (Ramos and Rafael 2017)
*Drosophilacruzloma* Llangarí & Rafael, 2018; H, A, P; (Llangarí and Rafael 2018)
*Drosophilacuasmali* Peñafiel & Rafael, 2018; H, P; (Peñafiel and Rafael 2018c)
*Drosophilacumanda* Llangarí & Rafael, 2018; H, A, P; (Llangarí and Rafael 2018)
*Drosophilacuyuja* Ramos & Rafael, 2015; H; (Ramos and Rafael 2015)
*Drosophiladeloscolorados* Llangarí & Rafael, 2020; H, P; (Llangarí and Rafael 2020)
*Drosophilaguacamayos* Ramos & Rafael, 2017; H, P; (Ramos and Rafael 2017)
*Drosophilaguajalito* Llangarí & Rafael, 2020; H, P; (Llangarí and Rafael 2020)
*Drosophilainti* Cabezas, Llangarí & Rafael, 2015; H, P; (Cabezas et al. 2015)
*Drosophilaintillacta* Cabezas & Rafael, 2013; H, P; (Cabezas and Rafael 2013)
*Drosophilakasha* Peñafiel & Rafael, 2019; H, P; (Peñafiel and Rafael 2019b)
*Drosophilakingmani* Peñafiel & Rafael, 2018; H; (Peñafiel and Rafael 2018a)
*Drosophilakurillakta* Peñafiel & Rafael, 2019; H; (Peñafiel and Rafael 2019a)
*Drosophilamachalilla* Acurio, Rafael, Céspedes & Ruiz, 2013; H, A, P; (Acurio et al. 2013)
*Drosophilamalacatus* Peñafiel & Rafael, 2018; H; (Peñafiel and Rafael 2018a)
*Drosophilamillmasapa* Peñafiel & Rafael, 2018; H, P; (Peñafiel and Rafael 2018a)
*Drosophilamisi* Peñafiel & Rafael, 2018; H, P; (Peñafiel and Rafael 2018b)
*Drosophilanapoensis* Ramos & Rafael, 2015; H, P; (Ramos and Rafael 2015)
*Drosophilaneoamaguana* Ramos & Rafael, 2017; H, A, P; (Ramos and Rafael 2017)
*Drosophilaneoasiri* Figuero & Rafael, 2013; H, P; (Figuero and Rafael 2013)
*Drosophilaneocapnoptera* Figuero & Rafael, 2013; H, A, P; (Figuero and Rafael 2013)
*Drosophilaneoprosaltans* Ramos & Rafael, 2017; H, A, P; (Ramos and Rafael 2017)
*Drosophilaneoyanayuyu* Ramos & Rafael, 2017; H, A, P; (Ramos and Rafael 2017)
*Drosophilanigua* Cabezas, Llangarí & Rafael, 2015; H, P; (Cabezas et al. 2015)
*Drosophilanina* Cabezas & Rafael, 2015; H, A, P; (Cabezas and Rafael 2015)
*Drosophilapapallacta* Figuero & Rafael, 2013; H, A, P; (Figuero and Rafael 2013)
*Drosophilapapaver* Tamayo & Rafael, 2016; H, A, P; (Tamayo and Rafael 2016)
*Drosophilapappobolusae* Figuero, León, Rafael & Céspedes, 2012; H, A, P; (Figuero et al. 2012a)
*Drosophilapichka* Peñafiel & Rafael, 2018; H, P; (Peñafiel and Rafael 2018a)
*Drosophilapodocarpus* Peñafiel & Rafael, 2019; H, P; (Peñafiel and Rafael 2019b)
*Drosophilapseudokorefae* Ramos & Rafael, 2018; H, P; (Ramos and Rafael 2018)
*Drosophilapseudomorelia* Ramos & Rafael, 2018; H, P; (Ramos and Rafael 2018)
*Drosophilaquijos* Ramos & Rafael, 2015; H, P; (Ramos and Rafael 2015)
*Drosophilaquinarensis* Peñafiel & Rafael, 2018; H, A, P; (Peñafiel and Rafael 2018b)
*Drosophilarucux* Céspedes & Rafael, 2012; H, A, P; (Céspedes and Rafael 2012a)
*Drosophilarusaryu* Peñafiel & Rafael, 2018; H, P; (Peñafiel and Rafael 2018a)
*Drosophilasachapuyu* Peñafiel & Rafael, 2018; H, A, P; (Peñafiel and Rafael 2018b)
*Drosophilasagittifolii* Llangarí & Rafael, 2017; H, A, P; (Llangarí and Rafael 2017)
*Drosophilasaraguru* Peñafiel & Rafael, 2019; H, P; (Peñafiel and Rafael 2019a)
*Drosophilashunku* Peñafiel & Rafael, 2018; H, A, P; (Peñafiel and Rafael 2018a)
*Drosophilashunkuku* Peñafiel & Rafael, 2018; H, A, P; (Peñafiel and Rafael 2018a)
*Drosophilasisapamba* Figuero, León, Rafael & Céspedes, 2012; H, P; (Figuero et al. 2012a)
*Drosophilataki* Peñafiel & Rafael, 2018; H, P; (Peñafiel and Rafael 2018a)
*Drosophilatinalandia* Llangarí & Rafael, 2018; H, A, P; (Llangarí and Rafael 2018)
*Drosophilatsachila* Llangarí & Rafael, 2020; H, P; (Llangarí and Rafael 2020)
*Drosophilavalenteae* Llangarí & Rafael, 2018; H, A, P; (Llangarí and Rafael 2018)
*Drosophilaverbesinae* Figuero, León, Rafael & Céspedes, 2012; H, A, P; (Figuero et al. 2012a)
*Drosophilawachi* Peñafiel & Rafael, 2019; H, A, P; (Peñafiel and Rafael 2019b)
*Drosophilawarmi* Peñafiel & Rafael, 2019; H, A, P; (Peñafiel and Rafael 2019a)
*Drosophilawayta* Figuero, León, Rafael & Céspedes, 2012; H, P; (Figuero et al. 2012a)
*Drosophilayambe* Cabezas, Llangarí & Rafael, 2015; H, P; (Cabezas et al. 2015)
*Drosophilayanacocha* Tamayo & Rafael, 2016; H; (Tamayo and Rafael 2016)
*Drosophilayanaurcus* Figuero, Rafael & Céspedes, 2012; H, A, P; (Figuero et al. 2012b)
*Drosophilayanayuyu* Céspedes & Rafael, 2012; H, A, P; (Céspedes and Rafael 2012a)
*Drosophilayurag* Figuero & Rafael, 2011; H, A, P; (Figuero and Rafael 2011)
*Drosophilayuragshina* Figuero & Rafael, 2011; H, P; (Figuero and Rafael 2011)
*Drosophilayuragyacum* Figuero, Rafael & Céspedes, 2012; H, A, P; (Figuero et al. 2012b)
*Drosophilazamorana* Peñafiel & Rafael, 2018; H, A, P; (Peñafiel and Rafael 2018b)
*Hirtodrosophilalojana* Peñafiel & Rafael, 2019; H, A, P; (Peñafiel and Rafael 2019b)
*Hirtodrosophilavillonacu* Peñafiel & Rafael, 2019; H; (Peñafiel and Rafael 2019b)
**Family Hybotidae**
*Elaphropezathoracica* Raffone, 2010; P; (Raffone 2010)
**Family Micropezidae**
*Cardiacephalaaeruginosa* Ferro & Marshall, 2018; H; (Ferro and Marshall 2018)
*Cardiacephalaaspera* Ferro & Marshall, 2018; H; (Ferro and Marshall 2018)
*Cardiacephalavitrata* Ferro & Marshall, 2018; H; (Ferro and Marshall 2018)
*Paragrallomyaecuadorensis* Ferro & Marshall, 2020; H; (Ferro and Marshall 2020)
**Family Neriidae**
*Longinaanguliceps* Buck & Marshall, 2004; P; (Buck and Marshall 2004)
*Longinasemialba* Buck & Marshall, 2004; P; (Buck and Marshall 2004)
**Family Psychodidae**
*Sycoraxwampukrum* Bravo & Salazar, 2009; H, P; (Bravo and Salazar 2009)
**Family Sphaeroceridae**
*Antropsanovariegatus* Kits & Marshall, 2013; H, P; (Kits and Marshall 2013)
*Antropsaurantifemur* Kits & Marshall, 2013; H, P; (Kits and Marshall 2013)
*Antropsbaeza* Kits & Marshall, 2013; H; (Kits and Marshall 2013)
*Antropsbellavista* Kits & Marshall, 2013; H, P; (Kits and Marshall 2013)
*Antropsbucki* Kits & Marshall, 2013; H; (Kits and Marshall 2013)
*Antropscotopaxi* Kits & Marshall, 2013; H, P; (Kits and Marshall 2013)
*Antropsdiversipennis* Kits & Marshall, 2013; H, P; (Kits and Marshall 2013)
*Antropseurus* Kits & Marshall, 2013; H; (Kits and Marshall 2013)
*Antropsfuliginosus* Kits & Marshall, 2013; H, P; (Kits and Marshall 2013)
*Antropsguandera* Kits & Marshall, 2013; H, P; (Kits and Marshall 2013)
*Antropspapallacta* Kits & Marshall, 2013; H, P; (Kits and Marshall 2013)
*Antropspecki* Kits & Marshall, 2013; H, P; (Kits and Marshall 2013)
*Antropsquadrilobus* Kits & Marshall, 2013; H, P; (Kits and Marshall 2013)
*Antropssierrazulensis* Kits & Marshall, 2013; H, P; (Kits and Marshall 2013)
*Antropstetrastichus* Kits & Marshall, 2013; H, P; (Kits and Marshall 2013)
*Antropsvariegatus* Kits & Marshall, 2013; H, P; (Kits and Marshall 2013)
*Aptilotellaangela* Luk & Marshall, 2014; H; (Luk and Marshall 2014)
*Aptilotellaebenea* Luk & Marshall, 2014; H; (Luk and Marshall 2014)
*Aptilotellagemmula* Luk & Marshall, 2014; H; (Luk and Marshall 2014)
*Aptilotellapichinchensis* Luk & Marshall, 2014; H; (Luk and Marshall 2014)
*Boreantropsauranticeps* Kits & Marshall, 2015; H, P; (Kits and Marshall 2015)
*Boreantropspollex* Kits & Marshall, 2015; H, P; (Kits and Marshall 2015)
*Boreantropssubemarginatus* Kits & Marshall, 2015; P; (Kits and Marshall 2015)
*Bromeloeciaabundantia* Yau & Marshall, 2018; H, P; (Yau and Marshall 2018)
*Bromeloeciaaculatus* Yau & Marshall, 2018; P; (Yau and Marshall 2018)
*Bromeloeciaaurita* Yau & Marshall, 2018; H, P; (Yau and Marshall 2018)
*Bromeloeciabalaena* Yau & Marshall, 2018; P; (Yau and Marshall 2018)
*Bromeloeciabrachium* Yau & Marshall, 2018; H, P; (Yau and Marshall 2018)
*Bromeloeciacercarcuata* Yau & Marshall, 2018; H, P; (Yau and Marshall 2018)
*Bromeloeciaconiclunis* Yau & Marshall, 2018; P; (Yau and Marshall 2018)
*Bromeloeciapinna* Yau & Marshall, 2018; H, P; (Yau and Marshall 2018)
*Bromeloeciaponsa* Yau & Marshall, 2018; H, P; (Yau and Marshall 2018)
*Bromeloeciaramus* Yau & Marshall, 2018; H, P; (Yau and Marshall 2018)
*Bromeloeciarobustora* Yau & Marshall, 2018; H, P; (Yau and Marshall 2018)
*Bromeloeciatriunguia* Yau & Marshall, 2018; H, P; (Yau and Marshall 2018)
*Bromeloeciaundulata* Yau & Marshall, 2018; H, P; (Yau and Marshall 2018)
*Bromeloeciawolverinei* Yau & Marshall, 2018; H, P; (Yau and Marshall 2018)
*Coproicabispatha* Bergeron, Marshall & Swann, 2015; P; (Bergeron et al. 2015)
*Coproicabrachystyla* Bergeron, Marshall & Swann, 2015; P; (Bergeron et al. 2015)
*Coproicadiabolia* Bergeron, Marshall & Swann, 2015; P; (Bergeron et al. 2015)
*Coproicagalapagosensis* Bergeron, Marshall & Swann, 2015; H, P; (Bergeron et al. 2015)
*Coproicanovacula* Bergeron, Marshall & Swann, 2015; P; (Bergeron et al. 2015)
*Leptocerapapallacta* Buck & Marshall, 2009; H, P; (Buck and Marshall 2009)
*Leptoceraplax* Buck & Marshall, 2009; P; (Buck and Marshall 2009)
*Minilimosinasclerophallus* Marshall, 1985; P; (Marshall 1985)
*Photoantropsechinus* Kits & Marshall, 2013; H; (Kits and Marshall 2013)
*Poecilantropsstellans* Kits & Marshall, 2013; H, P; (Kits and Marshall 2013)
**Family Syringogastridae**
*Syringogasteratricalyx* Marshall & Buck, 2009; P; MEPN; (Marshall et al. 2009)
*Syringogasterbrachypecta* Marshall & Buck, 2009; H, P; (Marshall et al. 2009)
*Syringogasterplesioterga* Marshall & Buck, 2009; P; MEPN; (Marshall et al. 2009)
**Family Tachinidae**
*Erythromelanacryptica* Inclan, 2013; P; (Inclan and Stireman 2013)
**Family Tanipezidae**
*Neotanypezamarshalli* Lonsdale, 2013; H, P; (Lonsdale 2013)
*Neotanypezaplotoplax* Lonsdale, 2013; H; (Lonsdale 2013)
*Neotanypezaposthos* Lonsdale, 2013; P; (Lonsdale 2013)
*Neotanypezavexilla* Lonsdale, 2013; H; (Lonsdale 2013)
**Family Tephritidae**
*Anastrephaamaryllis* Tigrero, 1998; H; (Tigrero 1998)
*Anastrephaanopla* Norrbom & Korytkowski, 2012; H, P; MEPN; (Norrbom and Korytkowski 2012)
*Anastrephagrandicanina* Norrbom & Korytkowski, 2012; P; MEPN; (Norrbom and Korytkowski 2012)
*Anastrephahadracantha* Norrbom & Korytkowski, 2012; H, P; MEPN; (Norrbom and Korytkowski 2012)
*Anastrephahaplacantha* Norrbom & Korytkowski, 2012; H; MEPN; (Norrbom and Korytkowski2012)
*Anastrephahyperacantha* Norrbom & Korytkowski, 2012; H, P; MEPN; (Norrbom and Korytkowski 2012)
*Anastrephaisolata* Norrbom & Korytkowski, 2009; H; MEPN; (Norrbom and Korytkowski 2009)
*Anastrephamacracantha* Norrbom & Korytkowski, 2012; H; MEPN; (Norrbom and Korytkowski 2012)
*Anastrephaneogigantea* Norrbom & Korytkowski, 2012; H; MEPN; (Norrbom and Korytkowski 2012)
*Molynocoeliaerwini* Norrbom, 2011; H; MEPN; (Norrbom 2011)
**Order Ephemeroptera**
**Family Leptophlebiidae**
*Atopophlebiapitculya* Flowers, 2012; H, A, P; (Flowers 2012)
**Order Hemiptera**
**Family Coreidae**
*Onoremiaacuminata* Brailovsky, 1995; H; (Brailovsky 1995)
**Order Hymenoptera**
**Family Apidae**
*Oxytrigonahuaoranii* González & Roubik, 2008; P; (González and Roubik 2008)
**Family Dryinidae**
*Gonatopussandovalae* Guglielmino, Olmi, & Speranza, 2016; H; (Guglielmino et al. 2016)
*Gonatopustapiai* Olmi & Guglielmino, 2016; H; (Olmi and Guglielmino 2016)
**Family Formicidae**
*Basicerosonorei* Baroni & De Andrade, 2007; H; (Baroni and De Andrade 2007)
*Leptanilloidescopalinga* Delsinne & Donoso, 2015; H; (Delsinne et al. 2015)
*Leptanilloidesprometea* Delsinne & Donoso, 2015; H, P; (Delsinne et al. 2015)
*Pachycondylacernua* Mackay & Mackay, 2010; P; (Mackay and Mackay 2010)
*Pyramicaheterodonta* Rigato & Scupola, 2008; P; (Rigato and Scupola 2008)
*Strumigenyslojaensis* Lattke & Aguirre, 2015; H; (Lattke and Aguirre 2015)
*Strumigenyslongimala* Baroni & De Andrade, 2007; H, P; (Baroni and De Andrade 2007)
*Strumigenysnageli* Baroni & De Andrade, 2007; H, P; (Baroni and De Andrade 2007)
*Strumigenysonorei* Baroni & De Andrade, 2007; H, P; (Baroni and De Andrade 2007)
**Family Halictidae**
*Chlerogellaeuprepia* Engel, 2010; P; (Engel 2010)
*Chlerogellamourella* Engel, 2003; P; (Engel 2003)
**Family Sphecidae**
*Pisonarachniraptor* Menke, 1988; P; (Menke 1988)
**Family Trichogrammatidae**
*Adryaserwini* Pinto & Owen, 2004; H, A; MEPN; (Pinto and Owen 2004)
*Pachamamaspeciosa* Owen & Pinto, 2004; P; MEPN; (Owen and Pinto 2004)
**Order Lepidoptera**
**Family Saturniidae**
*Automerisabdominapoensis* Brechlin & Meister, 2011; P; (Brechlin and Meister 2011a)
*Automerisabdomipichinchensis* Brechlin & Meister, 2011; P; (Brechlin and Meister 2011a)
*Automerisisabellae* Brechlin & Käch, 2017; P; (Brechlin et al. 2017)
*Automerismanzonoi* Brechlin, Käch & Meister, 2013; P; (Brechlin et al. 2013)
*Automerisparapichinchaensis* Brechlin & Meister, 2011; P; (Brechlin and Meister 2011a)
*Citheroniakaechi* Brechlin, 2019; P; (Brechlin et al. 2019)
*Copaxaandorientalis* Brechlin & Meister, 2012; P; (Brechlin and Meister 2012c)
*Copaxalitensis* Wolf & Colan, 2002; P; (Wolfe and Colan 2002)
*Dirphiaapeggyae* Brechlin, Meister & Käch, 2011; P; (Brechlin and Meister 2011c)
*Dirphiasachai* Brechlin & Käch, 2017; P; (Brechlin 2017)
*Gameliakaechi* Brechlin & Meister, 2012; P; (Brechlin and Meister 2012a)
*Hirpidakaechi* Brechlin, 2019; P; (Brechlin 2019)
*Perigabarragani* Brechlin, Käch & Meister, 2013; P; (Brechlin and Meister 2013)
*Rothschildiaariciaariciopichichensis* Brechlin, Käch & Meister, 2012; P; (Brechlin and Meister 2012b)
*Rothschildiaaricianapoecuadoriana* Brechlin & Meister, 2010; P; (Brechlin and Meister 2010)
*Rothschildiaincaincecuatoriana* Brechlin & Meister, 2012; P; (Brechlin and Meister 2012b)
*Rothschildialebecuatoriana* Brechlin & Meister, 2012; P; (Brechlin and Meister 2012b)
**Order Orthoptera**
**Family Tettigoniidae**
*Artiotonustinae* Montealegre, Morris, Sarria & Mason, 2011; H, A; (Montealegre et al. 2011)
*Supersonusundulus* Sarria, Morris, Windmill, Jackson & Montealegre, 2014; H, A; (Sarria et al. 2014)
**Order Trichoptera**
**Family Anomalopsychidae**
*Contulmapaluguillensis* Holzenthal & Ríos, 2012; P; (Holzenthal and Ríos 2012)
**Phylum Nemata**
**Class Secernentea**
**Order Strongylida**
**Family Molineidae**
*Neomolineuspierredesseti* Guerrero, 2020; P; MEPN; (Guerrero 2020)
**Family Strongyloididae**
*Parastrongyloidesneotropicalis* Guerrero, 2016; H, A; MEPN; (Guerrero 2016)
**Phylum Platyhelminthes**
**Class Cestoda**
**Order Phyllobothriidea**
**Family Phyllobothriidae**
*Clistobothriumamyae* Caira, Hayes & Jensen, 2020; H, P; MEPN; (Caira et al. 2020)
*Clistobothriumgabywalterorum* Caira, Hayes & Jensen, 2020; H; MEPN; (Caira et al. 2020)
*Scyphophyllidiumtimvickiorum* Caira, Hayes & Jensen, 2020; H, P; MEPN; (Caira et al. 2020)
**Order Tetraphyllidea**
**Family Serendipidae**
*Serendipdeborahae* Brooks & Barriga, 1994; H, P; MEPN; (Brooks and Barriga 1994)

**Figure 2.**
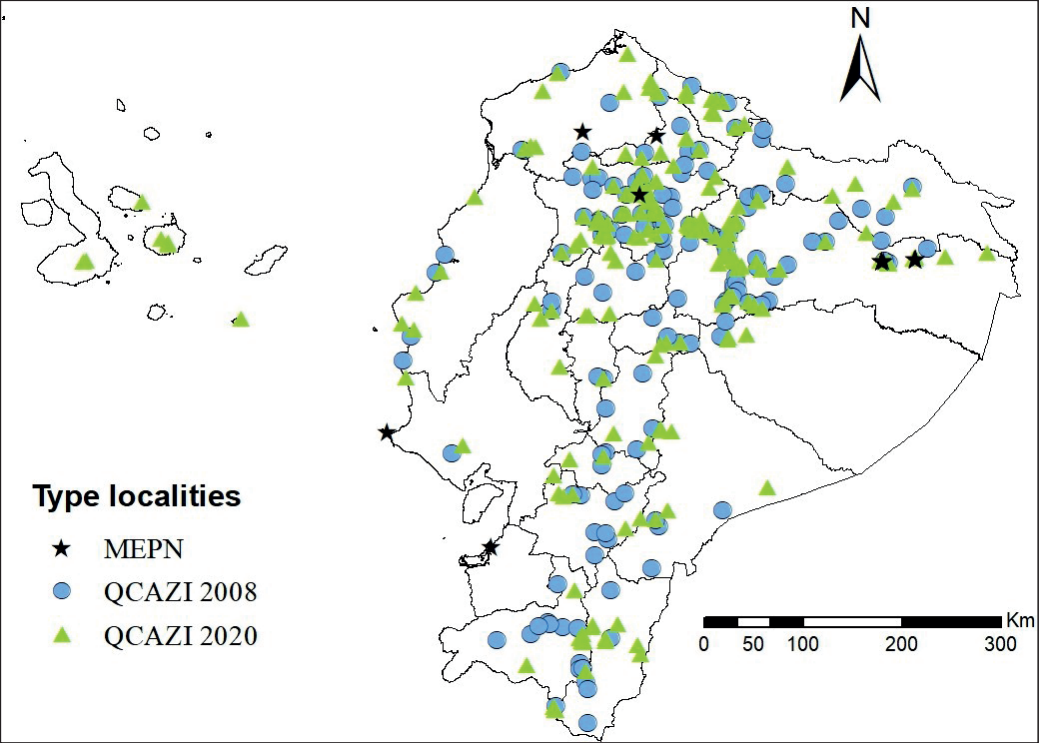
Geographical distribution of Ecuadorian type localities deposited at the MEPN and QCAZI museums showing the 24 provinces.

A map of type localities (Fig. 2) shows an increase of collection points compared to the ones published in Donoso et al. (2009). The new type specimens are distributed in all 24 provinces of Ecuador. Napo (96 spp.), Cotopaxi (93 spp.), and Pichincha (63 spp.) are the provinces with the highest number of new registered types. However, there are several provinces where more studies are required; this is the case for Bolívar (1 sp.), Cañar (2 spp.), El Oro (1 sp.), and Guayas (3 spp.), among others. Table 2 provides information for 71 type specimens (from 23 species or subspecies) from countries other than Ecuador.

**Table 2.** Type specimens from other countries.

**Phylum Arthropoda**
**Class Insecta**
**Order Coleoptera**
**Family Curculionidae**
*Pandeleteiuscampbelli* Howden, 1976; P; QCAZI; Colombia; (Howden 1976)
**Order Diptera**
**Family Clusiidae**
*Sobarocephalathrinax* Lonsdale & Marshall, 2012; P; QCAZI; Bolivia; (Lonsdale and Marshall 2012)
**Family Tachinidae**
*Erythromelanaarciforceps* Inclan, 2013; P; QCAZI; Brazil; (Inclan and Stireman 2013)
*Erythromelanacatarina* Inclan, 2013; P; QCAZI; Brazil; (Inclan and Stireman 2013)
*Erythromelanadistincta* Inclan, 2013; P; QCAZI; Brazil; (Inclan and Stireman 2013)
*Erythromelanaleptoforceps* Inclan, 2013; P; QCAZI; Brazil; (Inclan and Stireman 2013)
*Erythromelanawoodi* Inclan, 2013; P; QCAZI; Bolivia; (Inclan and Stireman 2013)
**Order Hymenoptera**
**Family Diapriidae**
*Turripriawoldai* Masner & García, 2002; P; QCAZI; Panamá; (Masner and García 2002)
**Family Encyrtidae**
*Anagyruslizanorum* Noyes & Menezes, 2000; P; MEPN; Costa Rica; (Noyes 2000)
*Anagyrusparalia* Noyes & Menezes, 2000; P; MEPN; Costa Rica; (Noyes 2000)
*Anagyrussinope* Noyes & Menezes, 2000; P; MEPN; Estados Unidos y Bahamas; (Noyes 2000)
*Blepyrushansoni* Noyes, 2000; P; MEPN; Costa Rica; (Noyes 2000)
*Blepyruszenonis* Noyes, 2000; P; MEPN; Costa Rica; (Noyes 2000)
*Gyranusoideaamasis* Noyes, 2000; P; MEPN; Costa Rica; (Noyes 2000)
*Gyranusoidearhodope* Noyes, 2000; P; MEPN; Costa Rica; (Noyes 2000)
*Hambletoniapilosifrons* Sharkov & Woolley, 1997; P; MEPN; Costa Rica; (Sharkov and Woolley 1997)
**Family Formicidae**
*Simopeltatransversa* Mackay & Mackay, 2008; P; QCAZI; Colombia; (Mackay and Mackay 2008)
**Phylum Mollusca**
**Class Gastropoda**
**Order Stylommatophora**
**Family Bulimulidae**
*Bostryxbermudezae* Weyrauch, 1958; P; MEPN; (Weyrauch 1958)
*Bostryxvilchezi* Weyrauch, 1960; P; MEPN; (Weyrauch 1960)
*Scutalusversicolorlachayensis* Weyrauch, 1967; P; MEPN; (Weyrauch 1967)
**Family Clausiliidae**
*Hemicenacerrateae* Weyrauch, 1958; P; MEPN; (Weyrauch 1958)
*Parabaleaomissa* (Weyrauch, 1957); P; MEPN; (Weyrauch 1957)
*Steerianacelendinensisisidroensis* Weyrauch & Zilch, 1954; P; MEPN; (Zilch 1954)

**Figure 3.**
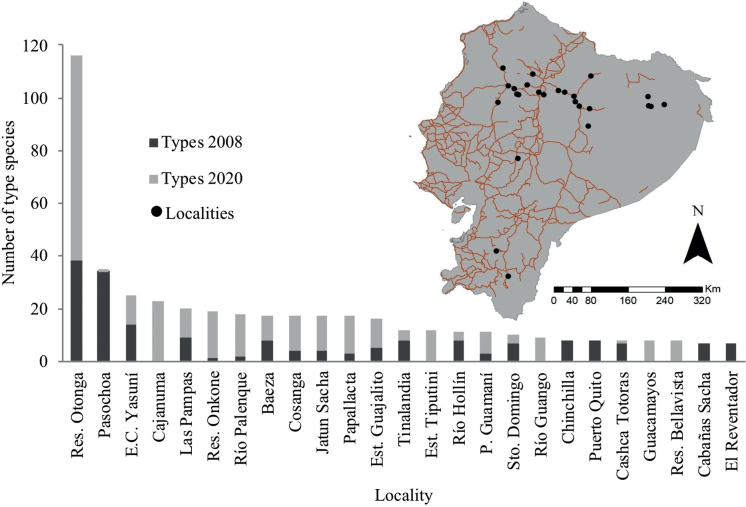
The twenty-five localities with the most type species registered in the 2008 and 2020 publications. We include a map of the main road system for the country and these 25 type localities.

With the new additions, 29% of described species of invertebrates were collected in five localities: Reserva Integral Otonga (116 spp.), Pasochoa (35 spp.), Estación Científica Yasuni (25 spp.), Cajanuma (23 spp.), and Las Pampas (20 spp.) (Fig. 3). Most type-rich localities were all found near the major roads of the country; except those found within Yasuni National Park (Fig. 3). 59% of localities provided type specimens for both 2008 and 2020 datasets. 19% of localities are new providers of type specimens, and the remaining 22% of localities provided type specimens for only the 2008 dataset. Insecta and Arachnida are the most abundant classes in the catalog. Insecta comprises eight orders, 43 families, 108 genera, and 327 species. From these, Diptera, with 1,473 type specimens, provides 65% of all types. In particular, Drosophilidae (71 species) and Sphaeroceridae (47 species) are the best-represented families in our catalog. Arachnida comprises seven orders, 20 families, 34 genera, and 111 species. Finally, we corrected information provided in the 2008 dataset, by removing nine species wrongly identified as type material (Table 3) and by correcting spelling mistakes in the names of four species (Table 4).

**Table 3.** Species excluded from the 2008 dataset.

**Phylum Arthropoda**
**Class Insecta**
**Order Lepidoptera**
**Family Nymphalidae**
*Manerebiagermaniae* Pyrcz & Hall, 2005
*Manerebiagolondrina* Pyrcz & Willmott, 2005
*Manerebiainderenaclara* Pyrcz & Willmott, 2005
*Manerebiainderenalaeniva* Pyrcz & Willmott, 2005
*Manerebiainderenamirena* Pyrcz & Willmott, 2005
*Manerebiainderenasimilis* Pyrcz & Willmott, 2005
*Manerebiasaturapauperata* Pyrcz & Willmott, 2005
*Manerebiaundulataundulata* Pyrcz & Willmott, 2005
*Pedaliodesmorenoipilaloensis* Pyrcz & Viloria, 1999

**Table 4.** Types that were misspelled in the 2008 dataset.

**Phylum Arthropoda**
**Class Insecta**
**Order Coleoptera**
**Family Cerambycidae**
*Apteraleidionlapierrei* Hovore, 1992 should be replaced with *Apteralcidionlapierrei* Hovore, 1992
**Family Curculionidae**
*BaillytesBartolozzi* Voisin, 1996 should be replaced with *Baillytesbartolozzii* Voisin, 1996
**Family Brentidae**
*Stereodermusjonathani* Mantilleri, 2004 is classified within the Brentidae family, not Rhysodidae.
**Order Diptera**
**Family Sphaeroceridae**
*Druciatustricetus* Marshall, 1995 should be replaced with *Druciatustrisetus* Marshall, 1995

## ﻿Discussion

The updated catalog endorses these two museums as benchmarks in invertebrate conservation at the national and international levels. The four largest orders of Insecta, Coleoptera, Diptera, Hymenoptera, and Lepidoptera that are highly diverse globally, are the focus of the highest number of studies in the country and are the best-represented insect groups in both collections. For example, Moret and Murienne (2020) described 25 carabid species of Andean Ecuador. Lonsdale and Marshall (2006, 2011, 2012) increased our understanding of Clusiidae dipterans in Ecuador with 21 new species. Since 2011, Adriano Kury, at the Museu Nacional at Rio de Janeiro (MNRJ, Brazil) has considerably curated the Opiliones collection at QCAZI museum (Kury 2012; Giupponi and Kury 2015). Unfortunately, a great number of these specimens were burned in the 2018 fire at the MNRJ (Kury et al. 2018) and it is only recently that some of these losses have been contextualized (Medrano et al. 2022).

The Arachnida collections in Ecuador are currently one of the best-represented and the most exhaustively studied. 90% of the Aranea species were described by Nadine Dupérré and Elicio Tapia in several publications as part of their project on a survey of Ecuadorian spiders (Dupérré 2022; Dupérré and Tapia 2020a). Pichincha, Cotopaxi, and Napo provinces are again reported as the most explored, and the invertebrates of many localities already listed in the 2008 catalog (e.g., Reserva Otonga and Estación Científica Yasuni) continue to be studied (Monte and Mascagni 2012; Erwin and Henry 2017; Flowers 2018). The southern provinces of Ecuador are starting to appear, i.e., Cajanuma, at the Parque Nacional Podocarpus, in Loja, where 19 species of Diptera: Drosophilidae were discovered and described by Peñafiel and Rafael (2018a, b, c; 2019a, b).

Taxonomists’ roles in studying invertebrates is crucial to increase biodiversity knowledge and promote its conservation. The lack of specialists in our country devoted to the study of taxonomy and the diversity of abundant and complex invertebrates prevents the rapid development of this subject needed for our region. In most cases, the specimens need to be sent out for identification to specialists overseas, and their studies can last several years. In this race against habitat loss in Ecuador, it is time to become aware and join efforts with government entities and academia to document and conserve our biodiversity in the hopes to achieve health, food, and clean water security, in other words, attain a place suitable for living.

## ﻿Additional information

### Author ORCIDs

Fernanda Salazar-Buenaño https://orcid.org/0009-0002-5633-909X

Diego Guevara https://orcid.org/0009-0008-3334-9790

Alvaro Barragán https://orcid.org/0000-0001-6843-2975

Vladimir Carvajal https://orcid.org/0000-0002-6201-063X

David A. Donoso https://orcid.org/0000-0002-3408-1457

### Data availability

All of the data that support the findings of this study are available in the main text or Supplementary Information.

## Supplementary materials

Supplementary material 1 Type specimen catalog with original information from their labels Data type text file Explanation note Type specimen catalog with original information from their labels. Organized in alphabetical order by class, order, family, genus, and species. The species taxonomic designation given in the initial publications is maintained in this catalog. https://binary.pensoft.net/file/873337This dataset is made available under the Open Database License (http://opendatacommons.org/licenses/odbl/1.0/). The Open Database License (ODbL) is a license agreement intended to allow users to freely share, modify, and use this Dataset while maintaining this same freedom for others, provided that the original source and author(s) are credited.Fernanda Salazar-Buenaño, Diego Guevara, Alvaro Barragán, Vladimir Carvajal, David A. Donoso
