# Supplementary material for: ﻿Catalog of the invertebrate type specimens hosted at the Pontificia Universidad Católica del Ecuador and Escuela Politécnica Nacional natural history collections
Source: Zookeys. 2023 Jul 5;1169:15–45. doi: 10.3897/zookeys.1169.102030 (PMC10339112; doi:10.3897/zookeys.1169.102030)
Supplement: Supplementary material 1 — Type specimen catalog with original information from their labels [file zookeys-1169-015_article-102030__-s001.docx]

**Supplementary Material 1**

Type specimen catalog with original information from their labels. Organized in alphabetical order by class, order, family, genus, and species. The species’ taxonomic designation given in the initial publications was maintained for this catalog.

**PHYLUM ARTHROPODA**

**CLASS ARACHNIDA**

**ORDER ARANEAE**

**FAMILY ANAPIDAE**

***Anapis anabelleae* Dupérré & Tapia, 2018**

Holotype QCAZI 254342 and Paratype QCAZI 254343. Label 1: Ecuador, Cotopaxi Prov. Otonga Bio. Reserve (00,41994° S 79,00623° W) 1997 m, 24 May–08 June 2014, E. Tapia, C. Tapia, N. Dupérré. Label 2: Beating epiphytes. Label 3: *Anapis anabelleae* 1 **♂** Holotype 1 **♀** Paratype

Comments: Holotype and Paratype preserved in the same vial

***Anapis carmencita* Dupérré & Tapia, 2018**

Holotype QCAZI 254336. Label 1: Ecuador, Cotopaxi Prov. Otonga Bio Reserve, río Esmeraldas (00,41941° S 78,6607° W) 1717 m, pitfall trap, E. Tapia, C. Tapia, N. Dupérré. Label 2: 16 Augt–5 Sep 2014. Anapidae (carmencita). Label 3: *Anapis carmencita* Det. N. Dupérré 2018. Label 4: **♂** Holotype

***Anapis churu* Dupérré & Tapia, 2018**

Holotype and Paratype QCAZI 254331. Label 1: Ecuador, Cotopaxi Prov. San Francisco de Las Pampas, Reserva Otonga 00,41994° S 79,00623°W 1997 msnm; N. Dupérré, E. E. Tapia, C. A. Tapia. Label 2: 24 May–08 Jun 2014. Label 3: *Anapis churu* Det. N. Dupérré 2018. Label 4: 1 **♂** Holotype; 1 **♂** Paratype

Comments: Holotype and Paratype preserved in the same vial

***Anapis mariebertheae* Dupérré & Tapia, 2018**

Holotype QCAZI 254340. Label 1: Ecuador, Cotopaxi Prov. Otonga Bio Reserve (00,41433° S 79,00035° W) 1888 m, pitfall trap; E. Tapia, C. Tapia, N. Dupérré. Label 2: 3–16 Aug. 2014. Anapidae 4. Label 3: *Anapis mariebertheae* Det. N. Dupérré 2018. Label 4: Holotype 1 **♂**

Paratype QCAZI 254332. Label 1: same data as the Holotype. Label 2: 7 Oct 2014. In tela de dipluridae *Anapis* sp. 4. Label 3: *Anapis mariebertheae* Det. N. Dupérré 2018. Label 4: Paratype 1 **♂**

***Anapis* *naranja* Dupérré & Tapia, 2018**

Holotype: QCAZI 254337. Label 1: Ecuador, Cotopaxi Prov. San Francisco de Las Pampas, Reserva Otonga 00,41994° S 79,00623° W, 1997 msnm, 13–15 Nov 2014, N. Dupérré, E. E. Tapia, C. A. Tapia. Label 2: sifting mosses, Berlese. Anapidae sp. 6. Label 3: *Anaspis naranja* 1 **♂** Holotype

Paratype QCAZI 254339. Label 1: Ecuador, Cotopaxi Prov. Otonga Bio Reserve (00,41433° S 79,00035° W) 1888 m, pitfall trap, 08–21 June 2014, E. Tapia, C. Tapia, N. Dupérré. Label 2: *Anaspis naranja* 1 **♀** Paratype

Paratype QCAZI 254341 **♂**. Label 1: Ecuador, Cotopaxi Pr. Otonga Bio Reserve (00,41433° S 79,00035° W) 1888 m, pitfall, N. Dupérré, E. Tapia, C. Tapia. Label 2: 13–25 Nov 2014. Anapidae sp. 6. Label 3: *Anaspis naranja* 1 **♂** Paratype

***Anapis nawchi* Dupérré & Tapia, 2018**

Holotype QCAZI 254338. Label 1: Ecuador, Cotopaxi Prov. Otonga Bio Reserve (00,41564° S 79,00452° W) 2105 m, pitfall trap, E. Tapia, C. Tapia, N. Dupérré. Label 2: 24 May–8 Jun 2014. *Anapis nawchi* 1 **♂** Holotype. Label 3: *Anapis nawchi* Det. N. Dupérré 2018

Paratype QCAZI 254344 Label 1: same data as the Holotype. Label 2: 3–16 viii 2014. *Anapis nawchi*. Label 2: Paratype 1 **♀**

***Anapis shina* Dupérré & Tapia, 2018**

Holotype QCAZI 254333, Paratypes QCAZI 254334 and QCAZI 254335. Label 1: Ecuador, Cotopaxi Prov. San Francisco de Las Pampas, Reserva Otonga 00,41994° S 79,00623° W 1997 msnm; N. Dupérré, E. E. Tapia, C. A. Tapia. Label 2: 13–15. xi. 2014 in mosses Berlese. Anapidae sp. 4. Label 3: *Anapis shina* Det. N. Dupérré 2018. Label 4: **♂** Holotype, 2 **♀** Paratypes

Comments: Holotype and Paratypes preserved in the same vial

**FAMILY ANYPHAENIDAE**

***Katissa guayasamini* Dupérré & Tapia, 2016**

Holotype QCAZI 3221. Label 1: Ec: Otonga Bio. Res. 24–30 May 2014; Beating tres, E. Tapia, C. Tapia, N. Dupérré. Label 2: *Katissa guayasamini* Holotype **♂**

***Katissa kurusiki* Dupérré & Tapia, 2016**

Holotype QCAZI 3225 and QCAZI 3226. Label 1: Ec: Cotopaxi Pr. Otonga Biol. Res.; 8–21 June 2014; sifting moss, 1717 m. E. Tapia, C. Tapia, N. Dupérré. Label 2: *Katissa kurusiki* Holotype **♂** Paratype **♀**

Comments: Holotype and Paratype preserved in the same vial

***Katissa puyu* Dupérré & Tapia, 2016**

Holotype QCAZI 3219 and QCAZI 3220. Label 1: Ec: Otonga Bio. Res. 21 June 2014, sift. Moss, Foothill; E. Tapia, C. Tapia, N. Dupérré. Label 2: **♂** Holotype **♀** Paratype

Comments: Holotype and Paratype preserved in the same vial

***Katissa tamya* Dupérré & Tapia, 2016**

Holotype QCAZI 3204. Label 1: Ec: Otonga Bio. Res. 21 Jun 2014, sifting moss foothill, E. Tapia, C. Tapia, N. Dupérré. Label 2: *Katissa tamya* **♂** Holotype

***Katissa yaya* Dupérré & Tapia, 2016**

Holotype QCAZI 3217. Label 1: Ec: Otonga Bio. Res. 4–7 Sept 2014; Hand coll. In moss, E. Tapia, C. Tapia, N. Dupérré. Label 2: *Katissa yaya* **♂** Holotype

***Patrera hatunkiru* Dupérré & Tapia, 2016**

Holotype QCAZI 3207. Label 1: Ec: Otonga Bio. Res. 4–7 Sept 2014; 2105 m, -00.41564° S, -79.00425° W, Beating trees, E. Tapia, C. Tapia, N. Dupérré. Label 2: *Patrera hatunkiru* **♂** Holotype

***Patrera philipi* Dupérré & Tapia, 2016**

Holotype QCAZI 3210. Label 1: Ec: Otonga Bio. Res. 4–7 Sept 2014; Hand coll. In moss., E. Tapia, C. Tapia, N. Dupérré. Label 2: *Patrera philipi* **♂** Holotype

***Patrera shida* Dupérré & Tapia, 2016**

Holotype QCAZI 3265. Label 1: Ec: Otonga Bio. Res. 24–30 May 2014, hand coll. E. Tapia, C. Tapia and N. Dupérré leg. Label 2: *Patrera shida* 1 **♂** Holotype

***Patrera suni* Dupérré & Tapia, 2016**

Holotype QCAZI 3224. Label 1: Ec: Otonga Bio. Res., 4–7 Sept. 2014, 1700 m, hand coll. E. Tapia, C. Tapia, N. Dupérré leg. Label 2: *Patrera suni* 1 **♂** Holotype

***Patrera witsu* Dupérré & Tapia, 2016**

Holotype QCAZI 3227. Label 1: Ec: Otonga Bio. Res. 24–30 May 2014, night collecting, E. Tapia, C. Tapia and N. Dupérré leg. Label 2: *Patrera witsu* Holotype 1 **♂**

***Shuyushka achachay* Dupérré & Tapia, 2016**

Holotype QCAZI 3223. Label 1: Ec: Otonga Bio. Res. 24–30 May 2014, beating trees, E. Tapia, C. Tapia and N. Dupérré. Label 2: *Shuyushka achachay* 1 **♂** Holotype

***Shuyushka moscai* Dupérré & Tapia, 2016**

Holotype QCAZI 3205. Label 1: Ec: Otonga Bio. Res. 24–30 May 2014, beating trees, E. Tapia, C. Tapia and N. Dupérré leg. Label 2: *Shuyushka moscai* **♂** Holotype

***Shuyushka wachi* Dupérré & Tapia, 2016**

Holotype QCAZI 3208. Label 1: Ec: Otonga Bio. Res. 24–30 May 2014, sifting moss, E. Tapia, C. Tapia and N. Dupérré leg. Label 2: Shuyuska wachi **♂** Holotype

**FAMILY CAPONIIDAE**

***Nops cesari* Dupérré, 2014**

Holotype QCAZI 3193 and Paratype QCAZI 3194. Label 1: Casa Cesar Tapia, 13 Sept 2013, sifting litter, E. Tapia. Label 2: *Nops cesari* 1 **♂** Holotype 1 **♀** Paratype

Comments: Holotype and Paratype preserved in the same vial

***Nops quito* Dupérré, 2014**

Holotype QCAZI 3188. Label 1: Ecuador, Pichincha, Quito 2810 m, 00° W, 00' S, 10 Oct 98, S. Castelo. Label 2: *Nops quito* Holotype

***Nyetnops juchuy* Dupérré, 2014**

Holotype QCAZI 3190. Label 1: Ec. Los Ríos. CCRP, 30 Ago 1977, S. Sandoval. Label 2: *Nyetnops juchuy* 1 **♂** Holotype

**FAMILY CTENIDAE**

***Chococtenus cappuccino* Dupérré, 2015**

Holotype QCAZI 3252 and Paratype QCAZI 3253. Label 1: Ec: Otonga Biol. Stat. 24 May–08 June 2014, S00.41433, W79.00035, 1888 m, pitfall, E. Tapia, C. Tapia, N. Dupérré. Label 2: *Chococtenus cappuccino* 1 **♂** Holotype 1 **♀** Paratype

***Chococtenus cuchilla* Dupérré, 2015**

Holotype QCAZI 3260. Label 1: Ec: Otonga Biol. Stat. 8–21 June 2014, 00.41564 S, 79.00425 W, pitfall, 2105 m, E. Tapia, C. Tapia, N. Dupérré. Label 2: *Chococtenus cuchilla* 1 **♂** Holotype

Paratype QCAZI 3261. Label 1: Ec: Otonga Biol. Stat. 24 May–8 June 2014, Night collecting, 2105 m, E. Tapia, N. Dupérré, C. Tapia. Label 2: *Chococtenus cuchilla* 1 **♀** Paratype

***Chococtenus duendecito* Dupérré, 2015**

Holotype QCAZI 3242. Label 1: Ec: Otonga Biol. Stat. 3–16 August 2014 00.41433 S, 79.00035 W, 1888 m, pitfall, E. Tapia, C. Tapia, N. Dupérré. Label 2: *Chococtenus duendecito* 1 **♂** Holotype

***Chococtenus fantasma* Dupérré, 2015**

Holotype QCAZI 3258. Label 1: Ec: Otonga Biol. Stat. 8 May–21 June 2014, S00.41564 W79.00425, 2105 m, pitfall, E. Tapia, C. Tapia, N. Dupérré. Label 2: *Chococtenus fantasma* Holotype 1 **♂**

Paratype QCAZI 3259: same data as holotype except 24. v.–08. vi. 2014. Label 2: *Chococtenus fantasma* Paratype 1 **♀**

***Chococtenus kashkara* Dupérré, 2015**

Holotype QCAZI 3236. Label 1: Ec: Otonga Biol. Res. 16 Ag–5 Sept 2014, S00.42261, W79.5107, 2225 m, pitfall, E. Tapia, C. Tapia, N. Dupérré. Label 2: *Chococtenus kashkara* Holotype 1 **♂**

Paratype QCAZI 3237. Label 1: Ec: Otonga Biol. Rese. 24 May–08 June 2014, hand collecting, E. Tapia, C. Tapia, N. Dupérré. Label 2: *Chococtenus kashkara* Paratype 1 **♀**

***Chococtenus lasdamas* Dupérré, 2015**

Holotype QCAZI 3238. Label 1: Ec: Otonga Biol. Stat. 23 July–5 August 2013, 00.39506 S, 78.98100 W, 1209 m, pitfall, E. Tapia, N. Dupérré. Label 2: *Chococtenus lasdamas* 1 **♂** Holotype

Paratypes QCAZI 3239 and QCAZI 3240. Label 1: Ec: Otonga Biol. Stat. Las Damas 1209 m, 5–16 Aug 2014, S00.39506, W78.98100, pitfall, E. Tapia, C. Tapia, N. Dupérré. Label 2: *Chococtenus lasdamas* 2 **♀** Paratype

***Chococtenus luchoi* Dupérré, 2015**

Holotype QCAZI 3241. Label 1: Ecuador, Cotopaxi Province, Otonga Biological Reserve (-00.42261° S -79.5107° W), 05–19. ix. 2014, pitfall, 2225 m, E. Tapia, C. Tapia, N. Dupérré. Label 2: *Chococtenus luchoi* Holotype 1 **♂**

***Chococtenus neblina* Dupérré, 2015**

Holotype QCAZI 3262. Label 1: Ecuador, Cotopaxi Province, Otonga Biological Reserve, 21 June–02 July 2014, pitfall, E. Tapia, C. Tapia, N. Dupérré. Label 2: *Chococtenus neblina* Holotype **♂**

Paratypes QCAZI 3263 and QCAZI 3264. Label 1: Ecuador, Cotopaxi Province, Otonga Biological Reserve, 24 May–08 June 2014, 2105 m, night collecting, E. Tapia, C. Tapia, N. Dupérré. Label 2: *Chococtenus neblina* Paratypes 2 **♀**

***Chococtenus otonga* Dupérré, 2015**

Holotype QCAZI 3244. Label 1: Ec: Otonga Bio. Res. 05–19. ix. 2014, pitfall, 1997 m, N. Dupérré, E. Tapia, C. Tapia. Label 2: Chococtenus otonga **♂** Holotype

Paratype QCAZI 3245. Label 1: Ec: Otonga Biol. Stat. 24 May–08 June 2014, hand collecting, E. Tapia, C. Tapia, N. Dupérré. Label 2: *Chococtenus otonga* **♀** Paratype

***Chococtenus otongachi* Dupérré, 2015**

Holotype QCAZI 3254 and Paratype QCAZI 3255. Label 1: Ec, Otongachi Biol. Sta. 03 April 2015, night collecting, E. Tapia. Label 2: *Chococtenus otongachi* Holotype **♂** Paratype **♀**

***Chococtenus piemontana* Dupérré, 2015**

Holotype QCAZI 3243. Label 1: Ecuador, Santo Domingo de los Tsáchilas, Otonga Biological Reserve, Las Damas (-00.39506° S -78.98100° W), 23. vii–05. viii. 2014, pitfall, 1209 m, E. Tapia, C. Tapia, N. Dupérré. Label 2: *Chococtenus piemontana* Holotype 1 **♂**

***Chococtenus suffuscus* Dupérré, 2015**

Holotype QCAZI 3246. Label 1: Ecuador, Pichincha Province, La Unión del Toachi, Centro de Educación Ambiental Otongachi, 03. iv. 2015, night collecting, E. Tapia. Label 2: *Chococtenus suffuscus* **♂** Holotype 5 **♀** Paratypes

Paratypes QCAZI 3247 and QCAZI 3248. Label 1: same data as holotype. Label 2: *Chococtenus suffuscus* **♂** Holotype 5 **♀** Paratypes

***Chococtenus waitti* Dupérré, 2015**

Holotype QCAZI 3232. Label 1: Ecuador, Cotopaxi Province, Otonga Biological Reserve (-00.41564° S -79.00425° W), 03–16. viii. 2014, pitfall, 2105 m, E. Tapia, C. Tapia, N. Dupérré. Label 2: *Chococtenus waitti* Holotype **♂**

**FAMILY DIPLURIDAE**

***Linothele pukachumpi* Dupérré & Tapia, 2015**

Holotype QCAZI 251097. Label 1: Ecuador, Cotopaxi Pr, Otonga Biol. Reserve, S00,42261, W79,5107, 2225 m, hand collected, in web with symbiot *Mysmenopsis otonga*, 04–09. ix. 2014, N. Dupérré, E. Tapia and C. Tapia. Label 2: Dipluridae *Linothele pukachumpi* Holotype

Paratype QCAZI 251095. Label 1: Same data as holotype, except: 26. v. 2014, hand. Label 2: Dipluridae *Linothele pukachumpi* Paratype

***Linothele quori* Dupérré & Tapia, 2015**

Holotype QCAZI 251088. Label 1: Ecuador, Santo Domingo de Las Tsáchilas, Parr. San José de Alluriquín, La Florida S00.25254, W79.03043, 884 m, 20. xi. 2014, hand collected from female's web, with *Mysmenopsis chiquita* symbiont, E. Tapia, N. Dupérré. Label 2: *Linothele quori* **♂** Holotype

Paratype QCAZI 251089. Label 1: same data as the holotype. Label 2: E. Tapia, N. Dupérré, collected in web with 1 **♂** 1 **♀** *M. chiquita*. *Linothele quori* **♀** Paratype

***Linothele tsachilas* Dupérré & Tapia, 2015**

Holotype QCAZI 251096. Label 1: Ecuador, Santo Domingo de Las Tsáchilas, Parr: San José de Alluriquín, La Florida (00.25254° S 79.03043° W), 884 m, and collected in web 17. xii. 2014, E. Tapia. Label 2: Dipluridae *Linothele tsachilas* Holotype

Paratype QCAZI 251090. Label 1: same data as the holotype except: 30. xi. 2014, hand collected from web. Label 2: *Linothele tsachilas* 1 **♀** Paratype

***Linothele yanachanka* Dupérré & Tapia, 2015**

Holotype QCAZI 251092. Label 1: Ecuador, Casa César Tapia, Cotopaxi Pr. 8. xii. 2014, hand colleted in web, E. Tapia y C. Tapia. Label 2: *Linothele yanachanka* Holotype **♀** (with *M. onorei*)

Paratypes **♂**, **♀** QCAZI 251093 and QCAZI 251094. Label 1: Ecuador, Cotopaxi Province, Parroquia San Francisco de Las Pampas, Casa César Tapia, 1426 m, 25. xii. 2014, hand collected form web with juveniles *M.* *onorei*, symbionts, E. Tapia, I. Tapia y C. Tapia. Label 2: Dipluridae *Linothele yanachanka* Paratype

***Linothele zaia* Dupérré & Tapia, 2015**

Holotype QCAZI 251091. Label 1: Ecuador, Santo Domingo de Las Tsáchilas, Parroquia San José de Alluriquín, La Florida (00.25254° S, 79.03043° W) 884 m, 17. xii. 2014, hand collected form web with *Mysmenopsis fernandoi*, E. Tapia. Label 2: *Linothele zaia* **♀** Holotype

**FAMILY MYSMENIDAE**

***Mysmenopsis alvaroi* Dupérré & Tapia, 2020**

Holotype **♂** QCAZI 260440. Label 1: Ecuador, Cotopaxi Pro. San Ramon 1051 m, -01.14891° -79.12135° 6 Mar 2019, Ex: *Linothele* E. Tapia. Label 2: *Mysmenopsis alvaroi* Det. N. Dupérré 2019, 1 **♂** Holotype

Allotype **♀** QCAZI 260444. Label 1: same data as the holotype. Label 2: ECFN 1162 *Mysmenopsis alvaroi* Det. N. Dupérré 2019, 1 **♀** Allotype

Paratypes **♀** QCAZI 260445 y QCAZI 261446. Label 1: same data as the holotype. Label 2: *Mysmenopsis alvaroi* Det. N. Dupérré 2019, 1 **♀** Paratype

***Mysmenopsis amazonica* Dupérré & Tapia, 2020**

Holotype QCAZI 260428. Label 1: Ecuador, Napo province, Misahuallí, Vía Arajuno (01.090117 -77.543352) 458 m, 19 May 2019, in Lycosidae web, E. E. Tapia. Label 2: *Mysmenopsis amazonica* Det. N. Dupérré 2019, 1 **♂** Holotype

Paratype QCAZI 260429. Label 1: same data as the holotype. Label 2: *Mysmenopsis* *amazonica* Det. N. Dupérré 2019, 1 **♂** Paratype

***Mysmenopsis angamarca* Dupérré & Tapia, 2020**

Holotype **♀** QCAZI 260400. Label 1: Ecuador, Cotopaxi Prov. Angamarca San Pablo -01.13214° -78.92961° 2500 m, 5 May 2016, In *Linothele* web Elicio E. Tapia. Label 2: *Mysmenopsis* *angamarca* Det. N. Dupérré 2019, 1 **♀** Holotype

***Mysmenopsis awa* Dupérré & Tapia, 2020**

Holotype QCAZI 260616. Label 1: Ecuador, Esmeraldas Prov. Alto Tambo, Sector Palo Amarillo 620 m, 00.97681° -78.56061°, 17 Mar 2019, Ex *Linothele* con (ECFN 1206) Elicio E. Tapia ECFN 1218. Label 2: *Mysmenopsis* *awa* Det. N. Dupérré 2019, 1 **♂** Holotype

Allotype **♀** QCAZI 260617. Label 1: same data as the holotype. Label 2: *Mymenopsis awa* Det. N. Dupérré 2019, 1 **♀** Allotype

***Mysmenopsis baerti* Dupérré & Tapia, 2020**

Holotype **♂** QCAZI 260430. Label 1: Ecuador, Zamora Chinchipe Province, Tepuy Guanza (04.14622, -7867509); 1527 m, 5 March 2016, E. E. Tapia. Label 2: *Mysmenopsis baerti* Det. N. Dupérré 2019, 1 **♂** Holotype

Allotype **♀** QCAZI 260431. Label 1: Ecuador, Zamora Chinchipe Province, Tepuy Guanza (04.14633, -7867509) 1527 m, 5 March 2016, E. E. Tapia. Label 2: *Mysmenopsis baerti* Det. N. Dupérré 2019, 1 **♀** Allotype

Paratype **♂** QCAZI 260610. Label 1: same data as the holotype. Label 2: *Mysmenopsis* baerti Det. N. Dupérré 2019, 1 **♂** Paratype

***Mysmenopsis bartolozzii* Dupérré & Tapia, 2020**

Holotype QCAZI 260411 and Allotype QCAZI 260412. Label 1: Ecuador, Pastaza Prov. Otoyacu Reserve -01.37128, -77.85436; 919 msnm, 12 July 2016, E. E. Tapia, In Linothele web. Label 2: *Mysmenopsis bartolozzii* Det. N. Dupérré 2019, 1 **♂** Holotype, 1 **♀** Allotype

Comments: Holotype and Allotype preserved in the same vial

Paratype QCAZI 260413 and QCAZI 260414. Label 1: same data as the holotype. Label 2: *Mysmenopsis bartolozzii* Det. N. Dupérré 2019, 1 **♂**, 1 **♀** Paratype

Comments: two Paratypes preserved in the same vial

***Mysmenopsis chiquita* Dupérré & Tapia, 2015**

Holotype QCAZI 251084 and QCAZI 251085. Label 1: Ecuador, Santo Domingo de Las Tsáchilas, Parroquia San José de Alluriquín, La Florida, 884 m, 28. x. 2014, hand collected in diplurid web, E. Tapia. Label 2: *Mysmenopsis chiquita* 1 **♂** Holotype, 1 **♀** Paratype

Comments: Holotype and Paratype preserved in the same vial

***Mysmenopsis choco* Dupérré & Tapia, 2020**

Holotype **♂** QCAZ 260447. Label 1: Ecuador, Esmeraldas Prov. Alto Tambo Reserva Otokiki, 735 m, 5 oct 2015, (-00.91271 -78.57110), *Linothele* Web, E. E. Tapia. Label 2: *Mysmenopsis choco* Det. N. Dupérré 2019, 1 **♂** Holotype

Allotype QCAZI 260448. Label 1: same data as the holotype. Label 2: *Mysmenopsis choco* Det. N. Dupérré 2019, 1 **♀** Allotype diss

Paratypes 2 **♂**, 1 **♀** QCAZI 260449. Label 1: Ecuador, Esmeraldas Prov. Alto Tambo Res. Otokiki (-00.91325 -78.565679), 638 m, 5 oct 2015, *Linothele* sp. web. E. Tapia, I. Tapia. Label 2: *Mysmenopsis choco* Det. N. Dupérré 2019, 2 **♂**, 1 **♀** Paratype

Comments: Three Paratypes preserved in the same vial with one QCAZ id number

Paratypes QCAZI 260450. Label 1: same data as the holotype. Label 2: *Mysmenopsis choco* Det. N. Dupérré 2019, 2 **♂** Paratype

Comments: Three Paratypes preserved in the same vial with one QCAZ id number

Paratype QCAZI 260599. Label 1: Ecuador, Esmeraldas Pro. Alto Tambo Res. Otokiki, -00.91271 -78.5711, 735 m, E. Tapia–I. Tapia; 5 oct 2015. Label 2: *Mysmenopsis choco* Det. N. Dupérré 2019, 1 **♀** Paratype

Paratype QCAZI 260612. Label 1: same data as the holotype, except: E. E. Tapia–N. Dupérré. Label 2: *Mysmenopsis* *choco* Det. N. Dupérré 2019, 1 **♂**, 2 **♀** Paratypes

Comments: Three Paratypes preserved in the same vial with one QCAZ id number

***Mysmenopsis corazon* Dupérré & Tapia, 2020**

Holotype QCAZI 260410. Label 1: Ecuador, Cotopaxi Prov. Vía Moraspungo–El Corazón Km 9, 836 m, 01.14891° -79.15154° 6 Mar 2019, Ex. *Linothele* sp. Web, E. E. Tapia. Label 2: *Mysmenopsis* *corazon* Det. N. Dupérré 2019, 1 **♂** Holotype

***Mysmenopsis cube* Dupérré & Tapia, 2020**

Holotype QCAZI 260441 and Allotype QCAZI 260442. Label 1: Ecuador, Esmeraldas Pr. Laguna de Cube, 27 Dec. 2016, -00.456336 -79.588134, 350 m, *Linothele* web, E. E. Tapia, A. A. Tapia. Label 2: *Mysmenopsis cube* Det. N. Dupérré 2019, 1 **♂** Holotype, 1 **♀** Allotype

Comments: Holotype and Allotype preserved in the same vial

Paratypes QCAZI 260443. Label 1: Ecuador, Esmeraldas Pr. Laguna de Cube -00.456336 -79.588134, 350 m, 27 Dec. 2016, *Linothele* web, E. E. Tapia, A. A. Tapia. Label 2: *Mysmenopsis cube* Det. N. Dupérré 2019, 3 **♂**, 2 **♀** Paratypes

Comments: Five Paratypes preserved in the same vial, with one QCAZ id number

***Mysmenopsis fernandoi* Dupérré & Tapia, 2015**

Holotype QCAZI 251082 and Paratype QCAZI 251083. Label 1: Ecuador, Santo Domingo de Las Tsáchilas, Parr. San José de Alluriquín, La Florida, 884 m, 28 Oct 2014, S00.25254, W7903043, hand collected from Diplurid web in foothill forest, E. Tapia. Label 2: *Mysmenopsis fernandoi* 1 **♂** Holotype, 1 **♀** Paratype

***Mysmenopsis guanza* Dupérré & Tapia, 2020**

Holotype QCAZI 260407 and Allotype QCAZI 260408. Label 1: Ecuador, Zamora Chinchipe Province, Tepuy Guanza (04.14622 -78.67509) 1527 m, 5 March 2016, E. E. Tapia. Label 2: *Mysmenopsis guanza* Det. N. Dupérré 2019, 1 **♀** Holotype, 1 **♀** Allotype

Paratype QCAZI 260406. Label: same data as the holotype. Label 2: *Mysmenopsis guanza* Det. N. Dupérré 2019, 1 **♀** Paratype

***Mysmenopsis guayaca* Dupérré & Tapia, 2020**

Holotype **♂** QCAZI 260614 and Allotype **♀** QCAZI 260615. Label 1: Ecuador, Guayas, Prov. Río Chilca Playa 287 m, -02.60989° -79.43625° 17 Jul 2016, in Linothele web, second road dirt talus, N. Dupérré, E. E. Tapia, A. A. Tapia. Label 2: *Mysmenopsis* *guayaca* Det. N. Dupérré 2019, 1 **♂** Holotype 1 **♀** Allotype

Comments: Holotype and Allotype preserved in the same vial

Paratypes 4 **♂** QCAZI 260409. Label 1: same data as the holotype. Label 2: *Mysmenopsis guayaca* Det. N. Dupérré 2019, 4 **♂** Paratypes

Comments: Four Paratypes preserved in the same vial, with one QCAZ id number

***Mysmenopsis hunachi* Dupérré & Tapia, 2020**

Holotype QCAZI 260403. Label 1: Ecuador, Cotopaxi Prov. Vía Sigchos–Río Hunachi 2347 m, -0.657704° -78.876737°, 15 Mar 2019, Elicio E. Tapia, Ex *Linothele* negra verdosa. Label 2: *Mysmenopsis* *hunachi* Det. N. Dupérré 2019, 1 **♂** Holotype

***Mysmenopsis junin* Dupérré & Tapia, 2020**

Holotype QCAZI 260607 and Allotype QCAZI 260608. Label 1: Ecuador, Imbabura Pro. Comunidad de Junin -00.28879 -78.55665 1285 msnm, 4 Apr 2014 in *Linothele* sp. web, E. E. Tapia. Label 2: *Mysmenopsis junin* Det. N. Dupérré 2019, 1 **♂** Holotype, 1 **♀** Allotype

Paratypes QCAZI 260432. Label 1: same data as the holotype. Label 2: *Mysmenopsis* *junin* Det. N. Dupérré 2019, 4 **♂**, 3 **♀** Paratypes

Comments: Seven Paratypes preserved in the same vial, with one QCAZ id number

***Mysmenopsis lasrocas* Dupérré & Tapia, 2020**

Holotype QCAZI 260404. Label 1: Ecuador, Santo Domingo de Los Tsáchilas, Bosque Las Rocas; S00.46431° W79.19624°; 661 msnm, 19 Mar 2015, con horst Tara P–53, in *Linothele tsachila* web, N. Dupérré, E. E. Tapia. Label 2: *Mysmenopsis* *lasrocas* Det. Dupérré 2019, 1 **♂** Holotype

Allotype QCAZI 260405. Label 1: same data as the holotype. Label 2: *Mysmenopsis lasrocas* Det. Dupérré 2019, 1 **♀** Allotype

***Mysmenopsis lloa* Dupérré & Tapia, 2020**

Holotype QCAZI 260605. Label 1: Ecuador, Pichincha Pro. Lloa via Mindo, sector Palmira km 16.4, 2719 msnm S00.22151° W78.64677° 31 Jan 2016, N. Dupérré, E. E. Tapia, A. A. Tapia. Label 2: *Mysmenopsis lloa* Det. N. Dupérré 2019, 1 **♀** Holotype

***Mysmenopsis onorei* Dupérré & Tapia, 2015**

Holotype QCAZI 251080. Label 1: Ecuador: Cotopaxi Pr. Casa Cesar Tapia, 1426 m, August 2013, hand collecting, Cesar y Carmen Tapia. Label 2: *Mysmenopsis onorei* 1 **♂** Holotype, 1 **♀** Paratype

Paratype QCAZI 251081. Label 1 and Label 2: same data as the holotype

***Mysmenopsis otokiki* Dupérré & Tapia, 2020**

Holotype QCAZI 260415 and Allotype QCAZI 260416. Label 1: Ecuador, Esmeraldas Prov. Alto Tambo Res. Otokiki 00.91271 -78.5711, 735 m, E. Tapia–I. Tapia; 5 Oct 2015. Label 2: *Mymenopsis* *otokiki* Det. N. Dupérré 2019, 1 **♂** Holotype, 1 **♀** Allotype

Comments: Holotype and Allotype preserved in the same vial.

Paratype QCAZI 260417. Label 1: same data as the holotype. Label 2: *Mymenopsis otokiki* Det. N. Dupérré 2019, 1 **♀** Paratype QCAZ

***Mysmenopsis otonga* Dupérré & Tapia, 2015**

Holotype QCAZI 251086 and Paratype QCAZI 251087. Label 1: Ecuador, Cotopaxi Province, Otonga Biological Reserve, S00.42261 W79.5107, 2225 m, hand collected in Dipluridae webs. Label 2: 04–07 Sept. 2014. *Mysmenopsis otonga* 1 **♂** Holotype, 1 **♀** Paratype

***Mysmenopsis pululahua* Dupérré & Tapia, 2020**

Holotype QCAZI 260436. Label 1: Ecuador, Pichincha, Termas de Pululahua 00.059720° 78.509230° 2128 m, 3 Feb 2019. In *Linothele* web of, Elicio E. Tapia. Label 2: *Mysmenopsis* *pululahua* Det. N. Dupérré 2019, 1 **♂** Holotype

Allotype QCAZI 260439. Label 1: same data as the holotype. Label 2: *Mysmenopsis* *pululahua* Det. N. Dupérré 2019, 1 **♀** Allotype

Paratypes **♀** QCAZI 260437 and QCAZI 260438. Label 1: same data as the holotype. Label 2: *Mysmenopsis pululahua* Det. N. Dupérré 2019, 1 **♀** Paratype

***Mysmenopsis salazarae* Dupérré & Tapia, 2020**

Holotype **♂** QCAZI 260422 and Allotype **♀** QCAZI 260423. Label 1: Ecuador, Pastaza Pro. Puyo, Santa Clara, Vía 9 de Octubre, Río Pucayacu, Otoyacu Natural Reserve -01.37128° -77.85436°, 919 m, 12 Jul 2016, hand collected in *Linothele* web. Leg. Elicio E. Tapia, ECFN 2461. Label 2: *Mysmenopsis salazarae* Det. N. Dupérré 2019, 1 **♂** HOLOTYPE, 1 **♀** Allotype

Comments: Holotype and Allotype preserved in the same vial

Paratype **♀** QCAZI 260424. Label 1: Ecuador, Pastaza Pro. Otoyacu, 919 m, 12 Jul 2016, E. E. Tapia, hand collected in *Linothele* web. Label 2: *Mysmenopsis* *salazarae* Det. N. Dupérré 2019, Paratype 1 **♀**, 1 juv

Comments: Two specimens preserved in the same vial, with one QCAZ id number.

Paratypes 2 **♀** QCAZI 260619. Label 1: same data as the holotype. Label 2: *Mysmenopsis salazarae* 2 **♀** Det. N. Dupérré 2019, PARATYPE

***Mysmenopsis shushufindi* Dupérré & Tapia, 2020**

Holotype **♂** QCAZI 260418. Label 1: Ecuador, Sucumbíos, Lago Agrio via Shushufindi Sector la Guanta -00.6647° -7690675° 350 msnm, 29 Abr. 2016, collected in Lycosidae web, N. Dupérré, E. E. Tapia. Label 2: *Mysmenopsis* *shushufindi* Det. N. Dupérré 2019, 1 **♂** Holotype

Allotype **♀** QCAZI 260419. Label 1: same data as the holotype, except: 20 Abr 2016. Label 2: *Mysmenopsis shushufindi* Det. N. Dupérré 2019, 1 **♀** Allotype

Paratype **♀** QCAZI 260420. Label 1: same data as the holotype. Label 2: *Mysmenopsis shushufindi* Det. N. Dupérré 2019, 1 **♀** Paratype

Paratype **♂** QCAZI 260606. Label 1: same data as the holotype. Label 2: *Mysmenopsis* *shushufindi* Det. N. Dupérré 2019, Paratype 1 **♂**

***Mysmenopsis tepuy* Dupérré & Tapia, 2020**

Holotype **♀** QCAZI 260611. Label 1: Ecuador, Zamora Chinchipe Province, Tepuy Guanza (04.14633 -78.67509) 1527 m, 5 March 2016, E. E. Tapia. Label 2: *Mysmenopsis tepuy* Det. N. Dupérré 2019, 1 **♀** Holotype

***Mysmenopsis tungurahua* Dupérré & Tapia, 2020**

Holotype **♂** QCAZI 260609. Label 1: Ecuador, Tungurahua, Via Baños-Penipe 2121 m, -01.41315° -78.46967° 18 Feb 2019, de *Linothele* web, E. Tapia. Label 2: *Mysmenopsis tungurahua* Det. N. Dupérré 2019, 1 **♂** Holotype

**FAMILY OCHYROCERATIDAE**

***Ochyrocera callaina* Dupérré, 2015**

Holotype **♂** QCAZI 261449 and 2 **♀** Paratypes QCAZI 261450 and QCAZI 261451. Label 1: Ec: Cotopaxi Pr., Otonga Biol. Reserve 00,42261° S, 79,5107° W, sifting litter, 24–30. v. 2014, N. Dupérré, E. Tapia, C. Tapia. Label 2: *Ochyrocera callaina* Holotype **♂**, Paratypes **♀**

Comments: Three specimens in the same vial

***Ochyrocera cashcatotoras* Dupérré, 2015**

Holotype QCAZI 3191. Label 1: Ecuador, Bolivar, Cashcatotoras 3000 m, 01°42'27" N, 02–06 Oct 2000, L. Coloma, F. Maza. Label 2: Holotype

***Ochyrocera italoi* Dupérré, 2015**

Holotype QCAZI 3187. Label 1: Ecuador, Cotopaxi, Otonga 78°57'00" W, 00°19'11" W, 2000 m, 17 Mar 1997, I. G. Tapia, P. Ponce. Label 2: *Ochyrocera italoi* Dupérré. Label 3: Holotype **♂**

***Ochyrocera losrios* Dupérré, 2015**

Holotype QCAZI 3192. Label 1: Ecuador, Pichincha CCRP colección hab. Unesco, 2 Dec 1980, bosque 2 Cer, S. Sandoval. Label 2: *Ochyrocera losrios* Dupérré Holotype

***Ochyrocera minotaure* Dupérré, 2015**

Holotype QCAZI 3189. Label 1: Ecuador, Pichincha, Nono, 18–I–1990, G. Quezada. Label 2: *Ochyrocera minotaure* 1 **♂** Holotype

***Ochyrocera otonga* Dupérré, 2015**

Holotype QCAZI 261440. Label 1: Ecu: Cotopaxi Pr. Otonga Bio. Reserve 0042261' S 79,5107° W, 1888 m, pitfall, 03–16 Aug 2014, N. Dupérré, E. Tapia. Label 2: *Ochyrocera otonga* **♂** Holotype

***Ochyrocera rinocerotos* Dupérré, 2015**

Holotype QCAZI 261447 and Paratype QCAZI 261448. Label 1: Ec: Cotopaxi Pr., Otonga Biol. Res., 04–07 Sept. 2014, sifting litter Berlese, N. Dupérré, E. Tapia, C. Tapia. Label 2: *Ochyrocera rinocerotos* Holotype **♂** Paratype **♀**

Comments: Both specimens in the same vial

***Ochyrocera zabaleta* Dupérré, 2015**

Holotype QCAZI 261438. Label 1: Ec: Santo Domingo de las Tsáchilas, San José de Alluriquin, La Florida, Río Zabaleta 884 m, 00,25254 S, 79,03043 W, 28 Oct 2014, Hand collected, E. Tapia. Label 2: *Ochyrocera zabaleta* 1 **♂** Holotype 1 **♀** Paratype

Comments: Holotype and Paratype preserved in the same vial

Paratype QCAZI 261439. Label 1 and Label 2: same data as the holotype

***Psiloochyrocera tortilis* Dupérré, 2015**

Holotype QCAZI 3186. Label 1: Ecuador, Río Palenque, 03–III–79, leg. Vargas. Label 2: *Psiloochyrocera tortillis* 1 **♂** Label 3: Holotype

***Speocera bioforestae* Dupérré, 2015**

Holotype QCAZI 261441 and Paratype QCAZI 261442. Label 1: Ec: Pichincha Pr. Otongachi, 26 Oct 2012, sifting litter, N. Dupérré, E. Tapia. Label 2: *Speocera bioforestae* Dupérré **♂** Holotype **♀** Paratype

Comments: Holotype and Paratype preserved in the same vial

***Speocera musgo* Dupérré, 2015**

Holotype QCAZI 261444, Paratypes QCAZI 261445 and QCAZI 261446. Label 1: Ec: Cotopaxi Pr., Otonga Biol. Reserve 00,42261° S 79,5107° W, 2225 m, collected in moss in arboles de 0,5 a 3 m, 15 Oct 2014, E. Tapia. Label 2: *Speocera musgo* Dupérré Holotype **♂** Paratypes 2 **♀**

Comments: Holotype and Paratypes in the same vial

***Speocera violacea* Dupérré, 2015**

Holotype QCAZI 261443. Label 1: Ec: Cotopaxi, Casa Cesar Tapia, nov 2013, Sifting litter, C. Tapia. Label 2: *Speocera violacea* **♂** Holotype

**FAMILY OONOPIDAE**

***Bipoonops lansa* Dupérré & Tapia, 2017**

Holotype QCAZI 251049. Label 1: Ec. Cotopaxi Pr. Otonga Biol. Res. 19 Sept–2 Oct 2014 S00,41433, W79,00035 1888 m, Pitfall, E. Tapia, C. Tapia, N. Dupérré. Label 2: *Bipoonops lansa* 1 **♂** Holotype

***Bipoonops pilan* Dupérré & Tapia, 2017**

Holotype QCAZI 251064. Label 1: Ec. Cotopaxi Pr. Otonga Bio. Res. 19 Sept–2 Oct 2014 (00,41433 79,0035) 1888 m, Pitfall, E. Tapia, C. Tapia, N. Dupérré. Label 2: *Bipoonops pilan* 1 **♂** Holotype

***Neotrops platnicki* Grismado & Ramírez, 2013**

Holotype QCAZI 261452. Label 1: Ecuador, Prov. Napo, Cantón Tena, Parroquia Puerto Napo, Estación Biológica Jatun Sacha S01°03'57,5", W77°37'00,2" (WGS84) 410 m, GPS(+/- 16 m), 1–5 Dic 2009. Col. C. Grismado & F. Labarque (PBI Expedition) Selva húmeda. Golpeteo de follaje Label 2: QCAZ *Neotrops platnicki* Grismado & Ramírez (Araneae, Oonopidae) **♂** Holotype

***Niarchos normani* Dupérré & Tapia, 2017**

Holotype QCAZI 251050. Label 1: Ec. Cotopaxi Pr. Otonga Bio. Res. 21 June 2014, Siftins moss, Berlese, E. Tapia, C. Tapia, N. Dupérré. Label 2: *Niarchos normani* 1 **♂** Holotype.

***Reductoonops berun* Dupérré & Tapia, 2017**

Holotype QCAZI 251055 and Paratype QCAZI 251056. Label 1: Ec. Cotopaxi Pr. Otonga Bio. Res. 4–7 ix. 2014 Sifting litter, Berlese, E. Tapia, C. Tapia, N. Dupérré. Label 2: *Reductoonops berun* 1 **♂** Holotype, 1 **♀** Paratype

Comments: Holotype and Paratype preserved in the same vial

Allotype QCAZI 251057. Label 1: same data as the holotype. Label 2: *Reductoonops berun* 1 **♀** Allotype

***Scaphidysderina chirin* Dupérré & Tapia, 2017**

Holotype QCAZI 251078 and Allotype QCAZI 251079. Label 1: Ec. Cotopaxi Pr. Otonga Bio. Res. -00,39506, W -78,98100, 1209 m. Las Damas Pitfall, 16. viii–03. ix. 2014, E. Tapia, C. Tapia, N. Dupérré. Label 2: *Scaphidysderina chirin* Holotype 1 **♂**, 1 **♀** Allotype

Comments: Holotype and Paratype preserved in the same vial

***Scaphidysderina lubanako* Dupérré & Tapia, 2017**

Holotype QCAZI 251070 and Allotype QCAZI 251071. Label 1: Ec. Cotopaxi Pr. Otonga Bio. Res. -00,39506, W -78,98100 1209 m, 28–vi–12 vii. 2014. Las Damas Pitfall, E. Tapia, N. Dupérré. Label 2: *Scaphidysderina lubanako* 1 **♂** Holotype, 1 **♀** Allotype

Comments: Holotype and Paratype preserved in the same vial

***Scaphidysderina tsaran* Dupérré & Tapia, 2017**

Holotype QCAZI 251074 and Allotype QCAZI 251075. Label 1: Ec. Cotopaxi Pr. Otonga Bio. Res. -00,41564, -79,00425, 2105 m, 24 v.–08 vi. 2014 Pitfall, E. Tapia, C. Tapia, N. Dupérré. Label 2: *Scaphidysderina tsaran* 1 **♂** Holotype, 1 **♀** Allotype

Comments: Holotype and Paratype preserved in the same vial

**FAMILY PARATROPIDIDAE**

***Paratropis elicioi* Dupérré, 2015**

Holotype QCAZI 3230. Label 1: Ec: Cotopaxi Pr. Otonga Biol. Reserve S00.41941, W78.99607, 1717 m, pitfall near Rio Esmeraldas, 25. xi–08 xii. 2014, N. Dupérré, E. Tapia. Label 2: *Paratropis elicioi* 1 **♂** Holotype

Paratype QCAZI 3231. Label 1: same data as the holotype, except 03–16 Aug 2014. Label 2: *Paratropis elicioi* **♀** Paratype

***Paratropis otonga* Dupérré & Tapia, 2020**

Holotype **♀** QCAZI 260733. Label 1: Ecuador, Cotopaxi pr. Otonga Biol. Reserve, Límite Sambo y Reserva (00.41395° S, 89.99085° W) 1728 m, 17 June 2015 Bajo troncos en el suelo, E. Tapia, N. Dupérré. Label 2: *Paratropis otonga* Dupérré & Tapia 2019 Det. N. Dupérré 2018 Holotype 1 **♀**

Paratype QCAZI 260734. Label 1: same data as the holotype. Label 2: *Paratropis otonga* Dupérré & Tapia 2019 Det. N. Dupérré 2018 Paratype 1 **♀**

***Paratropis pristirana* Dupérré & Tapia, 2020**

Holotype **♂** QCAZI 260716. Label 1: Ecuador, Cotopaxi Pro. Pristirana Natural Reserve, 1498 m, (-0.423418° -78.958775°) 26 Feb–5 Mar 2019, pitfall, E. E. Tapia & Tapia family. Label 2: *Paratropis pristirana* Dupérré–Tapia 2019 Det. N. Dupérré 2019. Label 3: 1 **♂** HOLOTYPE

Allotype **♀** QCAZI 260718. Label 1: Ecuador, Cotopaxi Pro. PRISTIRANA Natural Reserve -0.42492° -78.95708°, 1416 m, 22 March 2019; E. E. Tapia, FAMILY Tapia–Caisaguano. Label 2: *Paratropis pristirana* Dupérré -Tapia 2019 Det. N. Dupérré 2019, 1 **♀.** Label 3: ALLOTYPE

Paratype **♀** QCAZI 260715. Label 1: Ecuador, Cotopaxi Pro. PRISTIRANA Natural Reserve -0.424742° -78.959769°, 1521 m, 26 Feb–5 Mar 2019, E. Tapia, FAMILY Tapia. Label 2: *Paratropis pristirana* Dupérré, Tapia 2019 Det. N. Dupérré 2019, 1 **♀**. Label 3: PARATYPE

Paratype **♀** QCAZI 260719. Label 1: same data as the Holotype. Label 2: *Paratropis pristirana* Dupérré, Tapia 2019 Det. N. Dupérré 2019, 1 **♀**. Label 3: PARATYPE

Paratype **♀** QCAZI 260720 and QCAZI 260721. Label 1: Ecuador, Cotopaxi Pr. Pristirana Reserve, 1498 m, (-00,423418° -78,958775°), 16–26 Feb 2019 and 26 Feb–9 Mar 2019, pitfall E. E. Tapia & Tapia family. Label 2: *Paratropis pristirana* Dupérré, Tapia 2019 Det. N. Dupérré 2019 **♀** PARATYPE

**FAMILY SPARASSIDAE**

***Anaptomecus paru* Guala, Labarque & Rheims, 2012**

Holotype **♂** QCAZI 251798. Label 1: Ecuador: Prov. Santo Domingo de los Tsáchilas: Cantón Santo Domingo: Parroquia Santo Domingo: Tinalandia lodge, km 85 Road Aloaj (Aloag)–Santo Domingo. S00°19,262', W78°57',095' 758 m, 7 Dic 2009. Col. M. Ramírez, C. Grismado, M. Izquierdo & F. Labarque (PBI Expedition). Colecta manual. Label 2: Sparassidae Anaptomecus paru Guala, Labarque & Rheims **♂** Holotype

**FAMILY SYMPHYTOGNATHIDAE**

***Anapistula equatoriana* Dupérré & Tapia, 2017**

Holotype QCAZI 251072 and Paratype QCAZI 251073. Label 1: Ec. Cotopaxi Pr. Otonga Bio. Res. 4–7 Sept 2014 Sifting litter Berlese, E. Tapia, C. Tapia, N. Dupérré. Label 2: *Anapistula equatoriana* 1 **♂** Holotype, 1 **♀** Paratype

Comments: Holotype and Paratype preserved in the same vial

***Symphytognatha cabezota* Dupérré & Tapia, 2017**

Holotype QCAZI 251067 and Paratype QCAZI 251068. Label 1: Ec. Otonga Bio. Res., Cotopaxi Pr. 13–15 Nov 2014 Sfting litter Berlese, E. Tapia, C. Tapia, N. Dupérré. Label 2: *Symphytognatha cabezota* 1 **♂** Holotype, 1 **♀**, Paratype

Comments: Holotype and Paratype preserved in the same vial

**FAMILY TELEMIDAE**

***Kinku turumanya* Dupérré & Tapia, 2015**

Holotype QCAZI 3234. Label 1: Ec: Cotopaxi, Otonga Bio. Res. 00.42261 S, 79.5107 W, 8–21 June 2014, 2225 m, N. Dupérré, E. Tapia & C. Tapia. Label 2: *Kinku turunmanya* **♂** Holotype **♀** Paratype

Paratype QCAZI 3235. Label 1: same data as the holotype, except sifting moss. Label 2: *Kinku turunmanya* **♂** Holotype **♀** Paratype

**FAMILY THERIDIOSOMATIDAE**

***Chthonos kuyllur* Dupérré & Tapia, 2017**

Holotype QCAZI 251062. Label 1: Ec: Cotopaxi Pr. San Francisco de las Pampas, Casa César Tapia (-00,42413; -78,95719) 1426 m, 8 Aug–2013. Hand coll, C. Tapia, C. Caisaguano. Label 2: *Chthonos kuyllur* **♂** Holotype

Paratypes QCAZI 251059, QCAZI 251060 and QCAZI 251063. Label 1: Ec. Cotopaxi Pr. Otonga Bio. Res. 24–30 May 2014. Hand coll., E. Tapia, C. Tapia, N. Dupérré. Label 2: *Chthonos kuyllur* Paratype

***Naatlo mayzana* Dupérré & Tapia, 2017**

Holotype QCAZI 251066. Label 1: Ec. Cotopaxi Pr. Otonga Bio. Res. 5–7 Sep 2014 general coll., E. Tapia, C. Tapia, N. Dupérré. Label 2: *Naatlo mayzana* Holotype

***Ogulnius laranka* Dupérré & Tapia, 2017**

Holotype QCAZI 251053. Label 1: Ec. Cotopaxi Pr. Otonga Bio. Res. 4–7 Sept 2014 Sifting L. Her Berlese, E. Tapia, C. Tapia, N. Dupérré. Label 2: *Ogulnius laranka* 1 **♂** Holotype

Allotype QCAZI 251054. Label 1: same data as the holotype. Label 2: *Ogulnius laranka* **♀** Allotype

***Ogulnius paku* Dupérré & Tapia, 2017**

Holotype QCAZI 251051 and Paratype QCAZI 251052. Label 1: Ec. Cotopaxi Pr. Otonga Bio. Res. 5–7 Sept 2014 General coll., E. Tapia, C. Tapia, N. Dupérré. Label 2: *Ogulnius paku* 1 **♂** Holotype, 1 **♀** Paratype

Comments: Holotype and Paratype preserved in the same vial

***Theridiosoma ankas* Dupérré & Tapia, 2017**

Holotype QCAZI 251069. Label 1: Ec. Cotopaxi Pr. Otonga Bio. Res. 24–30 May 2014 S -00,42261, W -79,5107 2225 m, Night coll., cloud forest, E. Tapia, C. Tapia, N. Dupérré. Label 2: *Theridiosoma ankas* Holotype 1 **♂**

***Theridiosoma esmeraldas* Dupérré & Tapia, 2017**

Holotype QCAZI 251065. Label 1: Ec. Cotopaxi Pr. Otonga Bio. Res. -00,41941 S, -78,99607 W; 1717 m, Sifting moss, low evergreen montane forest, E. Tapia, C. Tapia, N. Dupérré. Label 2: *Theridiosoma esmeraldas* Holotype **♀**

***Theridiosoma kullki* Dupérré & Tapia, 2017**

Holotype QCAZI 251058. Label 1: Ec. Cotopaxi Pr. San Francisco de las Pampas, Casa César Tapia (-00,42413; -78,95719) 1426 m, 13. ix. 2013. Hand coll., premontane evergreen forest, C. Tapia & C. Caisaguano. Label 2: *Theridiosoma kullki* 1 **♂** Holotype

Paratype QCAZI 251061. Label 1: Ec. Pichincha Pr. Otongachi Biol. Res. -00,321205; -78,95163, 10. Xi. 2014. Sifting litler, premontane evergreen forest, E. Tapia, N. Dupérré. Label 2: *Theridiosoma kullki* 1 **♀** Paratype

***Theridiosoma sacha* Dupérré & Tapia, 2017**

Holotype QCAZI 251076 and Paratype QCAZI 251077. Label 1: Ec. Cotopaxi Pr. Otonga Bio. Res. 5–7 Sept. 2014 Hand coll. E. Tapia, C. Tapia, N. Dupérré. Label 2: *Theridiosoma sacha* 1 **♂** Holotype, 1 **♀** Paratype

Comments: Holotype and Paratype preserved in the same vial

**ORDER OPILIONES**

**FAMILY NEOGOVEIDAE**

***Metagovea ligiae* Giupponi & Kury, 2015**

Holotype QCAZI 251721. Label 1: Ecuador, Punto 10, Napo, Pacto Sumaco, S00.66577°; W077.59813° alt 1526 m, 15–16 Feb 2014, A. Kury, A. Giupponi leg. Label 2: *Metagovea ligiae* Holotype **♂** Paratype **♀**

Paratype QCAZI 251720. Label 1: same data as the holotype. Label 2: *Metagovea ligiae* Holotype **♂** Paratype **♀**

**FAMILY CRANAIDAE**

***Zannicranaus monoclonius* Kury, 2012**

Holotype QCAZI 2985 and Allotype QCAZI 2986. Label 1: Ecuador, Chimborazo, Sibambe, axila de mata barranco S02,22059°, W078,89622° alt: 2423 m, 29 Mar 2011, A. Giupponi & A. Kury leg. Label 2: Zannicranaus monoclonius Kury 2012, 1 **♂** Holótipo 1 **♀** Alótipo

***Zannicranaus morlaucus* Kury, 2012**

Holotype QCAZI 2987. Label 1: Ecuador, Azuay, Molleturo 3210 m, 02°48'227 S, 079°20'426 W; 27–XII–001 I. G. Tapia, G. Onore. Label 2: Zannicranaus morlaucus Kury 2012, 1 **♂** Holotype

**ORDER PSEUDOSCORPIONES**

**FAMILY WITHIIDAE**

***Cystowithius smithersi* Harvey, 2004**

Paratypes QCAZI 2659 and QCAZI 2660. Label 1: Ecuador, Carchi Province, Volcán Chiles, c. 15 km N of Tulcán 0°48'35,0" N, 77°57'15.0" W, 3600 m, August 1997; P. Smithers, A. Bond, M. Burne; in leaf sheath of *Espeletia pycnophylla*. Label 2: Cystowithius smithersi Harvey Paratypes 1 **♂** 1 **♀** 1 TN

**ORDER RICINULEI**

**FAMILY RICINOIDIDAE**

***Cryptocellus chiruisla* Botero & Flórez, 2017**

Holotype QCAZI 3440. Label 1: Ecuador, Orellana, Chiruisla km 0,2 primary forest, 218 m, elev. 00°36'50" S, 75°52'34" W, 8–13 Dic 2005, J. Vieira, Winkler trap. Label 2: Paratype QCAZI 3441. Label 1: same data as the holotype. Label 2: Paratypes 1 female and 1 tritonymph

Comments: Specimens have not been deposited in the museum yet, but they were assigned QCAZ id numbers as they will be deposited when logistics permit it

**ORDER SARCOPTIFORMES**

**FAMILY LOHMANNIIDAE**

***Lohmannia vulcania* Schatz, 1993**

Paratype QCAZI 2661. Label 1: Galapagos, Isabela–v. s. Negra, 1987–02–12, Schatz. Label 2: GA 87–653

***Torpacarus remotus* Schatz, 1994**

Paratype QCAZI 2662. Label 1: Galapagos, Bartolomé, Littoral, 1986–12–26, Schatz. Label 2: GAL G040

**ORDER SCHIZOMIDA**

**FAMILY HUBBARDIIDAE**

***Surazomus kitu* Villarreal, Silva & Giupponi, 2016**

Holotype QCAZI 251707. Label 1: Ecuador, Pichincha (Los Ríos), C. C. Río Palenque 00°54' S, 79°00' W, 220 m, 7 Jan 1981, S. Sandoval. Ex Bosque Secundario. Label 2: *Surazomus kitu* sp. n.

***Surazomus palenque* Villarreal, Silva & Giupponi, 2016**

Holotype QCAZI 251723. Label 1: Ecuador, Pichincha (Los Ríos), C. C. Río Palenque 00°54' S, 79°00' W, 220 m, 27 Dec 1980, S. Sandoval. Ex: Palma. Label 2: Holotype

Paratype QCAZI 251718 and Paratype QCAZI 251719. Label 1: same data as the holotype, except: 29 Dec 1980 and 21 Dec 1980. Ex Bosque Secundario. Label 2: Paratype

Paratype QCAZI 251724. Label 1: same data as the holotype. Label 2: Paratype

**ORDER SCORPIONES**

**FAMILY CHACTIDAE**

***Teuthraustes kuryi* Ythier & Lourenco, 2017**

Holotype QCAZI 251708. Label 1: ECUADOR–02–ESMERALDAS, caminho lamacento em zona de derrubada, N00,88883° W078,53732°, alt: 873 m, 17 Mar 2011, A. Chagas, A. Giupponi, A. Kury & M. Vega leg. Label 2: *Teuthraustes kuryi* **♀** Holotype

**CLASS CHILOPODA**

**ORDER GEOPHILOMORPHA**

**FAMILY BALLOPHILIDAE**

***Ityphilus grismadoi* Pereira, 2018**

Holotype **♀** QCAZI 251797. Label 1: Ecuador, Provincia de Orellana: Cantón Francisco de Orellana: Parroquia Puerto Francisco de Orellana: Rio Tiputini, Estación científica Yasuní, 0°40'27" S, 76°23'50" W; ca. 295 m, a. s. l., 1–5 December 2009. Col. M. Ramírez (PBI Expedition), rain forest, in leaf litter, Berlese extraction. Label 2: *Ityphilus grismadoi* sp. nov. Female HOLOTYPE (with 39 leg–bearing segments, body lenght 9 mm)

**FAMILY** **SCHENDYLIDAE**

***Pectiniunguis aequatorialis* Pereira, 2018**

Holotype **♂** QCAZI 251796. Label 1: Ecuador, Napo province: Cayambe–Coca Ecological Reserve, road to Papallacta, Oyacachi S00°16'20,0", W078°05'37,2", elevation 3823 m, a. s. l. (error 8 m) GPS, 1 December 2009. M. Izquierdo, N. Platnick, N. Dupérré, A. Bonaldo & E. Tapia (PBI expedition), Paramo landscape (in leaf litter) *Pectiniunguis aequatorialis* sp. nov. L. A. Pereira det. Male. Label 2: Holotype (with 45 leg–bearing segments, body length 19 mm)

**CLASS COPEPODA**

**ORDER SIPHONOSTOMATOIDA**

**FAMILY CALIGIDAE**

***Pupulina mantensis* Cruz, Caña, Suárez & Santana, 2018**

Holotype **♂** QCAZI 3450. Label 1: Host: *Aetobatus narinari*. Holotype **♂** *Pupulina mantensis*. 6/2/2015. Los Esteros.

Allotype **♀** QCAZI 3452. Label 1: same data as the Holotype, except: Alotipo **♀**

Paratype **♀** QCAZI 3451. Label 1: same data as the Holotype. except: Paratype **♀**

**CLASS DIPLOPODA**

**ORDER POLYDESMIDA**

**FAMILY PLATYRHACIDAE**

***Barydesmus* *nangaritza* Recuero & Sánchez, 2018**

Holotype QCAZI 251005. Label 1: Ecuador, Zamora Chinchipe, Nangaritza, Las Orquideas 1040 m, 4°14' S, 78°39' W, 8–XII–2016, A. Sánchez. Label 2: *Barydesmus nangaritza* Recuero & Sanchez–Vialas. Holotype

**CLASS INSECTA**

**ORDER COLEOPTERA**

**FAMILY CANTHARIDAE**

***Maronius papallactae* Constantin, 2007**

Paratype QCAZI 1963 and QCAZI 2172. Label 1: Ecuador, prov. Napo Papallacta 9 km east mountain forest 00°22' S, 78°04' W, 2730 m, 22. XI. 2006, R. Constantin. Label 2: PARATYPE *Maronius papallactae* Constantin, 2008

Paratype QCAZI 1964. Label 1: same data as the holotype, except: 23. XI. 2006. Label 2: PARATYPE *Maronius papallactae* Constantin, 2008

Paratype QCAZI 2173: Label 1: Ecuador, prov. Napo, Papallacta, 5 km east mountain forest 0°22' S; 78°04' W, 2730 m, 21. XI. 2006, R. Constantin. Label 2: PARATYPE *Maronius papallactae* Constantin, 2008

***Plectonotum crassicorne* Constantin, 2008**

Paratype QCAZI 2040. Label 1: Ecuador prov. Azuay Gualaceo 20 km east paramos, 03°00' S, 78°39' W, 3395 m, 19. XI. 2006, R. Constantin. Label 2: PARATYPE, *Plectonotum crassicorne* Constantin n. sp. R. Constantin det. 2008

***Plectonotum glaber* Constantin, 2008**

Paratypes QCAZI 2046 and QCAZI 2047. Label 1: Ecuador prov. Napo Papallacta 6 km E edge mountain forest, 00°22' S, 78°04' W, 2750 m, 3. XII. 2007a. R. Constantin. Label 2: PARATYPE, *Plectonotum glaber* Constantin n. sp. R. Constantin det. 2008

***Plectonotum latithorax* Constantin, 2008**

Paratype QCAZI 2039. Label 1: Ecuador prov. Azuay, Gualaceo 20 km east paramos, 03°00' S, 78°39' W, 3396 m, 19. XI. 2006, R. Constantin. Label 2: PARATYPE, *Plectonotum latithorax* Constantin n. sp. R. Constantin design. 2008

***Plectonotum macaraense* Constantin, 2010**

Paratypes QCAZI 2370 and QCAZI 2371. Label 1: Ecuador, prov. Loja, Macará 30 km East 5 km E Utuana paso 4°20' S, 79°41' W, 2622 m, 14. V. 2010e, R. Constantin. Label 2: PARATYPE, *Plectonotum macaraense* Constantin n. sp. R. Constantin det. 2010

***Plectonotum moreti* Constantin, 2008**

Paratype QCAZI 2044. Label 1: Ecuador prov. Loja Saraguro 3 km S wet meadow, woody slope 03°39' S, 79°15' W, 2835 m, 27. XI. 2007a. R. Constantin. Label 2: PARATYPE *Plectonotum moreti* Constantin n. sp. R. Constantin des. 2008

***Plectonotum nigricorne* Constantin, 2008**

Paratype QCAZI 2045. Label 1: Ecuador prov. Azuay, Sigsig 7 km south paramos, 03°05' S, 78°47' W, 2850 m, 15. XI. 2006, R. Constantin. Label 2: PARATYPE, *Plectonotum nigricorne* Constantin n. sp. R. Constantin det. 2008

***Plectonotum onorei* Constantin, 2008**

Paratypes QCAZI 2041 and QCAZI 2042. Label 1: Ecuador prov. Napo Cosanga 8 km south mirador de la Virgen 00°37' S, 77°50' W, 2200 m, 5. XII. 2007b, R. Constantin. Label 2: PARATYPE, *Plectonotum onorei* Constantin n. sp. R. Constantin des. 2008

***Plectonotum puncticollis* Constantin, 2008**

Paratype QCAZI 2043. Label 1: Ecuador prov. Morona Sant. Lago Atillo 4 km east páramos bush, Asteraceae, 02°11' S, 78°28' W, 3112 m, 29. XI. 2007, R. Constantin. Label 2: PARATYPE, *Plectonotum puncticollis* Constantin n. sp. R. Constantin des. 2008

***Plectonotum zanjarajunoense* Constantin, 2010**

Paratype QCAZI 2372. Label 1: Ecuador, prov. Pastaza, 25 km NE of Puyo, Zanjarajuno, río Pukayacu 1°21' S, 77°51' W, 980 m, 18. V. 2010a, R. Constantin. Label 2: PARATYPE, *Plectonotum zanjarajunoense* Constantin n. sp. R. Constantin des. 2010

***Silis barragani* Constantin, 2010**

Holotype QCAZI 2375. Label 1: Ecuador, Prov. Cañar, Zhud 2 km north woody slope 2°26' S, 78°59' W, 2958 m, 16. V. 2010a, R. Constantin. Label 2: HOLOTYPE. Label 3: *Silis barragani* Constantin n. sp. R. Constantin des. 2008

***Silis elongatipennis* Constantin, 2009**

Paratypes QCAZI 2170 and QCAZI 2171. Label 1: Ecuador, prov. Morona Sant. Lago Atillo 6 km East páramos bush, Asteraceae 2°12' S; 78°28' W, 3078 m, 1. XII. 2007c. R. Constantin. Label 2: PARATYPE *Silis elongatipennis* Constantin 2009

***Silis gilletti* Constantin, 2009**

Paratype QCAZI 2181. Label 1: Ecuador, Imbabura, Los Cedros, 1350 m, 78°46760' W; 00°18500' N, 20–30 SEP 2005, R. Cárdenas. Ex: E3. Label 2: PARATYPE *Silis gilletti* Constantin 2009

Paratype QCAZI 2182 and QCAZI 2183. Label 1: Ecuador, Pichincha, E. C. Río Guajalito, 1800 m, 78°48'10" W; 00°13'53" S, 10 JUN 2005, J. García. Label 2: PARATYPE *Silis gilletti* Constantin 2009

***Silis otongae* Constantin, 2009**

Holotype QCAZI 2176. Label 1: Ecuador, Cotopaxi, San Fco. de las Pampas 78°55' 00°52'32" S, 4–8 Jul 2002, F. Baldessin. Label 2: PARATYPE, *Silis otongae* Constantin 2009

Paratype QCAZI 2177. Label 1: Ecuador, Cotopaxi, Otonga 79°00' W; 00°25' S, 21 JUL 1997, I. Tapia, P. Ponce. Label 2: PARATYPE, *Silis otongae* Constantin 2009

Paratype QCAZI 2178. Label 1: Ecuador, Pichincha, Nanegalito, 78°41'00" W; 00°08'00" N, 27 MAY 2005, D. Serrano. Label 2: PARATYPE, *Silis otongae* Constantin 2009

Paratype QCAZI 2179. Label 1: Ecuador, Cotopaxi, 1500 m, Las Pampas 78°57'04" W; 00°25'16" S (Malaise Trap), 10 Sep 1997 G. Onore. Label 2: PARATYPE, *Silis otongae* Constantin 2009

Paratype QCAZI 2180. Label 1: Ecuador, Pichincha, Nanegalito, 14 JAN. 1996, A. Barragán. Label 2: PARATYPE, *Silis otongae* Constantin 2009

**FAMILY CARABIDAE**

***Balligratus brevis* Moret & Ortuño, 2017**

Paratype QCAZI 251725. Label 1: Ecuador, 1. V. 85; Prov. Chimborazo, Tungurahua sud, Río Puela tronc pouri, Pierre Moret legit 2750 m. Label 2: PARATYPE, *Balligratus brevis* Moret & Ortuño 2017

***Balligratus humerangulus* Moret & Ortuño, 2017**

Paratype QCAZI 224724. Label 1: Ecuador, Pichincha, Nanegalito, m 2220, 27–VII–06. Labell 2: N00°00,207' W078°35,450' vaglio foresta. Labell 3: leg. C. Belló, G. Osella & M. Pagliano. Label 4: PARATYPE. Label 5: *Balligratus humerangulus* Moret & Ortuño 2017, P. Moret det. 2016

Paratypes QCAZI 251726 and QCAZI 251727. Label 1: Ecuador, Cotopaxi, Otonga, 1900 m, 07 Ago 1998, I. Tapia. Label 2: PARATYPE. Label 3: *Balligratus humerangulus* Moret & Ortuño 2017, P. Moret det. 2016

***Bembidion ricei* Maddison & Toledano, 2012**

Holotype QCAZI 2706. Label 1: Ecuador, Napo, Río Chalpi Grande, 2800 m, 0,3645° S 78,0852° W, 26. x. 2010, drm 10,159, W. P. & D. R. Maddison, M. Reyes. Label 2: HOLOTYPE, *Bembidion ricei* Maddison & Toledano

***Blennidus amaluzanus* Moret, 2005**

Paratype QCAZI 3202. Label 1: Ecuador, South Cordillera Llagunillas, East of Jimbura, 14–V–1998, 2600–3300 m, leg. A. Jasinski. Label 2: *Blennidus* (Sierrobius) *amaluzanus* Moret. Label 3: PARATYPE *Blennidus* (Sierrobius) *amaluzanus* Moret; P. Moret det. 2004

***Calleida desenderi* Casale, 2011**

Holotype **♂** QCAZI 2188. Label 1: Ecuador, Napo, Reventador, San Rafael, 1400 m, 10 JAN. 1998, F. Maza, Ex: ligh trap. Label 2: HOLOTYPUS, *Calleida desenderi* n. sp. A. Casale det. 2010

***Chlaenius walterrossii* Giachino & Allegro, 2018**

Paratype **♀** QCAZI 259340. Label 1: Ecuador, Orellana, Parque National Yasuni, Estacion Cientifica Yasuni, 13–15. IX. 2013, W. Rossi et al. Label 2: PARATYPUS *Chlaenius walterrossii* n. sp. P. M. Giachino, G. Allegro det 2018

***Diploharpus curtulus* Moret, 2008**

Paratypes QCAZI 2165 and QCAZI 2166. Label 1: Ecuador, Cotopaxi, Otonga, 1900 m, 07 Ago 1998, I. Tapia. Label 2: *Diploharpus curtulus* Moret PARATYPE

Paratype QCAZI 2167. Label 1: Ecuador, Cotopaxi, San Fco. de las Pampas Otonga, 2000 m, 25 FEB 1998, G. Onore. Label 2: *Diploharpus curtulus* Moret PARATYPE. Label 3: *Diploharpus curtulus* Moret. P. Moret det. 09

***Dyscolus aquator* Moret & Murienne, 2020**

Holotype QCAZI 259419. Label 1: Ecuador, Prov. Pichincha, Tandayapa, Bellavista Lodge WP81–2250 m, 2. XI. 2015, S 0°00'56.6"/W 78°40'49.1" Riparian, P. Moret leg. Label 2: *Dyscolus aquator* Moret & Murienne 2020 HOLOTYPE. Label 3: *Dyscolus aquator* Moret; P. Moret det. 2019

***Dyscolus arauzae* Moret & Murienne, 2020**

Holotype QCAZI 260394. Label 1: Ecuador, Prov. Pichincha, Cayambe, Suroeste WP 73, N0.006962/W78.020278 4405 m, 30–X–2015, P. Moret, M. Aráuz leg. Label 2: *Dyscolus arauzae*, Moret HOLOTYPE. Label 3: *Dyscolus arauzae*, Moret 2020, P. Moret det. 2020

***Dyscolus barragani* Moret & Murienne, 2020**

Holotype QCAZI 260398. Label 1: Ecuador, Prov. Chimborazo, Ayapungu–Cerro Púlpito, Estación 416, 4180 m, S 02°18'29.0"/W 78°34'38.8" P. Moret, A. Barragán, 23. III. 15. Label 2: *Dyscolus barragani*, Moret HOLOTYPE. Label 3: *Dyscolus barragani*, Moret 2020, P. Moret det. 2020

***Dyscolus crespoae* Moret & Murienne, 2020**

Holotype QCAZI 259398. Label 1: Ecuador, Prov. Zamora; PNP Estación El Colibrí; 2110 m, S 03°59'16.1"/W 79°05'39.0" P. Moret, C. Ruiz 18. III. 2015. Label 2: By night 19h30–21h on the ground. Label 3: *Dyscolus crespoae* Moret HOLOTYPE. Label 4: *Dyscolus crespoae* Moret, P. Moret det. 2019

***Dyscolus donosi* Moret & Murienne, 2020**

Holotype QCAZI 259396. Label 1: Ecuador, Prov. Zamora PNP Estación El Colibrí 2110 m, S 03°59'16.1"/W 79°05'39.0" P. Moret, C. Ruiz 18. III. 2015. Label 2: trunk 1 m, high. Label 3: *Dyscolus donosi* Moret HOLOTYPE. Label 4: *Dyscolus donosoi* Moret, P. Moret det. 2019

***Dyscolus eleonorae* Moret & Murienne, 2020**

Holotype QCAZI 249405. Label 1: Ecuador, loc Dos Rios c/o San Francisco de Las Pampas (2000 m), Feb 1993, L. Bartolozzi legit (num Mag 1406). Label 2: Dyscolus elenorae Moret HOLOTYPE. Label 3: *Dyscolus elenorae* Moret; P. Moret det. 2019

Paratype QCAZI 10247. Label 1: Ecuador, Pichincha, E. C. Río Guajalito, 1800 m, 78°48'10" W, 00°13'53" S, 23 APR 2005, A. Rodríguez. Label 2: *Dyscolus elenorae* Moret PARATYPE

Paratype QCAZI 74255. Label 1: Ecuador, Cotopaxi, Otonga, 1975 m, 79°00'00" W, 00°25'00" S, 11 JUL 2007, A. C. Proaño. Label 2: *Dyscolus elenorae* Moret PARATYPE

Paratype QCAZI 256855. Label 1: Ecuador, Pichincha, Est. Río Guajalito 1800 m, 78°48'10" W, 00°13'53" S, 17 ABR 2010, A. León. Label 2: *Dyscolus elenorae* Moret PARATYPE

Paratype QCAZI 256857. Label 1: Ecuador, Cotopaxi, Otonga, 2000 m, -0,41667 -79,0000, 30 MAY 2010, J. Torres. Label 2: Ex: Ficus Label 3: *Dyscolus elenorae* Moret PARATYPE

Paratype QCAZI 259406. Label 1: Ecuador, S. Domingo, XI–81, G. Onore. Label 2: *Dyscolus elenorae* Moret PARATYPE

Paratype QCAZI 259407. Label 1: Ecuador, Pichincha, Mindo, 7 Ago 1994, Ohio University. Label 2: *Dyscolus elenorae* Moret PARATYPE 07/08/1994

Paratype QCAZI 259408. Label 1: Ecuador, Cotopaxi, Los Libres 2000 m, 5 Nov 1994 Santiago Espinosa. Label 2: *Dyscolus elenorae* Moret PARATYPE 05/11/1994

Paratype QCAZI 259409. Ecuador, Cotopaxi, Las Pampas, Otonga, 1 Jan 1997, G. Onore. Label 2: *Dyscolus elenorae* Moret PARATYPE 01/01/1997

Paratype QCAZI 259410. Label 1: Ecuador, Cotopaxi, Otonga 1600 m, 78°57'00" W, 00°19'11" S, 2 Jul 2000, A. Pérez. Label 2: *Dyscolus elenorae* Moret PARATYPE 02/07/2000

Paratype QCAZI 259411. Label 1: Ecuador, Cotopaxi, Otonga 1800 m, 20 OCT 2000, I. G. Tapia. Label 2: *Dyscolus elenorae* Moret PARATYPE 19/10/2000

***Dyscolus famelicus* Moret & Murienne, 2020**

Holotype QCAZI 260395. Label 1: Ecuador, Prov. Napo, east of Papallacta–Guango Lodge, 24. X. 2015, WP 40–2708 m, S 0°22'42.6" W 78°04'26.6" Bromelia fogging/P. Moret leg. Label 2: *Dyscolus famelicus* Moret, Holotype. Label 3: *Dyscolus famelicus* Moret, P. Moret det. 2016

***Dyscolus giselae* Moret & Murienne, 2020**

Holotype QCAZI 259412. Label 1: 3-5. VII. 01, P. Moret, Ecuador, Cotopaxi, Otonga 1950 m, Beige de ruisseau. Label 2: *Dyscolus giselae* Moret HOLOTYPE. Label 3: *Dyscolus giselae* Moret; P. Moret det. 2019

Paratype QCAZI 259413. Label 1: Ecuador, Cotopaxi, Las Pampas, Otonga, 1 Jan 1997, G. Onore. Label 2: *Dyscolus giselae* Moret PARATYPE

Paratype QCAZI 259414. Label 1: Ecuador, Cotopaxi, Otonga 3000 m, 1 May 1997, J. Gil. Label 2: ex: debajo de piedras en el río. Label 2: *Dyscolus giselae* Moret PARATYPE

Paratype QCAZI 259415. Label 1: Ecuador, Cotopaxi, Otonga, 1900 m, 7 AUG. 1998, I. Tapia. Label 2: *Dyscolus giselae* Moret PARATYPE

***Dyscolus gobbii* Moret & Murienne, 2020**

Holotype QCAZI 259404. Label 1: Ecuador, Pichincha, 27. II. 17; Guamaní WP 210; 4230 m, S 0°19'18.3"/W 78°11'54.8" P. Moret, M. Gobbi leg. Label 2: *Dyscolus gobbii* Moret HOLOTYPE. Label 3: *Dyscolus gobbii* Moret P. Moret det. 2019

***Dyscolus incommunis* Moret & Murienne, 2020**

Holotype QCAZI 259416. Label 1: Ecuador, Prov. Pichincha, Tandayapa, Bellavista Lodge WP81–2250 m, S 0°00'56.6"/W 78°40'49.1"; Bromelia fogging/P. Moret leg. Label 2: Bromelia fallen on the ground. Label 3: *Dyscolus incommodus* Moret HOLOTYPE. Label 4: *Dyscolus incommodus* Moret P. Moret det. 2019

***Dyscolus marini* Moret & Murienne, 2020**

Holotype QCAZI 259399. Label 1: Ecuador, Loja, 17. III. 2015, Cajanuma–Refugio PNP, S 04°06'58.4"/W 79°10'18.6" 2850 m, P. Moret, C. Ruiz. Label 2: Bromelia. Label 3: *Dyscolus marini* Moret HOLOTYPE. Label 4: *Dyscolus marini* Moret, P. Moret det. 2019

Paratype QCAZI 74257. Label 1: Ecuador, Loja, Loja P. N. Podocarpus, Cajanuma 2750 m, 04°05' S 79°12' W, 28–30 DEC–2001 G. Buitrón J. Label 2: *Dyscolus marini* Moret PARATYPE

Paratype QCAZI 252423. Label 1: Ecuador, Loja, 17. III. 2015, Cajanuma–Estación 397; S 04°07'02.4"/W 79°10'06,3" 2900 m, P. Moret, C. Ruiz. Label 2: Bromelia Fogging 15h–16h. Label 3: PARATYPE. Label 4: *Dyscolus marini* Moret, PARATYPE, P. Moret det. 2019

***Dyscolus piscator* Moret & Murienne, 2020**

Holotype QCAZI 260393. Label 1: Ecuador, Napo, 11. III. 2017, Guamaní–Paso de la Virgen S 0°21'00.6"/W 78°11'52.4" 283–3890 m, P. Moret, M. Gobbi. Label 2: *Dyscolus* *piscator*, Moret, Holotype. Label 3: *Dyscolus* *piscator*, Moret 2020, P. Moret det. 2020

***Dyscolus placitus* Moret & Murienne, 2020**

Paratype QCAZI 256725. Label 1: Ecuador, Napo, East of Guamani, Paso de la Virgen, 3890 m, S 0°21'00.6" W 78°11'52,4", 11/03/2017, P. Moret & M. Gobbi leg. Label 2: *Dyscolus placitus* Moret, PARATYPE. Label 3: *Dyscolus placitus* Moret 2020, P. Moret det. 2016

***Dyscolus ravidus* Moret & Murienne, 2020**

Holotype QCAZI 259403. Label 1: Ecuador, Loja, 17. III. 2015; Cajanuma–Refugio PNP, S 04°06'58.4"/W 79°10'18.6" 2850 m, P. Moret, C. Ruiz. Label 2: By night 19h30–21h Bromelia. Label 3: *Dyscolus ravidus* Moret HOLOTYPE. Label 4: *Dyscolus ravidus* Moret, P. Moret det. 2019

Paratype QCAZI 252422. Label 1 and Label 2: same data as the holotype. Label 3: PARATYPE. Label 4: *Dyscolus ravidus*, Moret PARATYPE P. Moret det. 2019

Paratype QCAZI 259400. Label 1: Ecuador, Loja, Valladolid, límite del Parque Jocotoco y Podocarpus, 6 JAN. 2001, I. G. Tapia. Label 2: *Dyscolus ravidus* Moret PARATYPE

Paratype QCAZI 259401. Label 1: Ecuador, Loja, Par. Nacional Podocarpus 2800 m, 12 Feb 1994; G. Onore. Label 2: *Dyscolus ravidus* Moret PARATYPE

***Dyscolus rivinus* Moret & Murienne, 2020**

Holotype QCAZI 259417. Label 1: Ecuador, Cotopaxi, Otonga 2000 m, 7 JUL 1998, I. Tapia. Label 2: *Dyscolus rivinus* Moret, HOLOTYPE. Label 3: *Dyscolus rivinus* Moret; P. Moret det. 2019

Paratype QCAZI 259418. Label 1: same data as the holotype. Label 2: *Dyscolus rivinus* Moret, PARATYPE

***Dyscolus rugitarsis* Moret & Murienne, 2020**

Holotype QCAZI 259393. Label 1: Ecuador, Loja 4. VIII. 2016, P. N. Yacuri WP 167–3240 m, 4,711851° S/79,440355° W, Leaf litter at night, 19h–20h30, P. Moret, S. Aguirre, E. Moreno. Label 2: *Dyscolus rugitarsis* Moret HOLOTYPE. Label 3: *Dyscolus rugitarsis* Moret, P. Moret det. 2019

Paratype QCAZI 252417, QCAZI 259394 and QCAZI 259395. Label 1: Ecuador–Prov. Loja, Cordillera Lagunillas 3 S of Jimbura 3240 m, S 04,71198, W 79,44045, 11. VIII. 2013, P. Moret. Label 2: Upper montane forest; Leaf litter at night. Label 3: *Dyscolus rugitarsis* Moret PARATYPE

***Dyscolus ruizi* Moret & Murienne, 2020**

Holotype QCAZI 259402. Label 1: Ecuador, Loja, 17. III. 2015; Cajanuma–Estación 397, S 04°07'02.4"/W 79°10'06.3" 2900 m, P. Moret, C. Ruiz. Label 2: *Dyscolus ruizi* Moret HOLOTYPE. Label 3: *Dyscolus ruizi* Moret, P. Moret det. 2019

Paratype QCAZI 252424. Label 1: Ecuador, Loja, Cajanuma Sendero -4,113, -79,174, 2920 m, 11/07/2013 Marin & Ruiz. Label 2: PARATYPE. Label 3: PARATYPE, P. Moret det. 2019

***Dyscolus silvestris* Moret & Murienne, 2020**

Holotype QCAZI 260396. Label 1: Ecuador, Prov. Napo, Termas de Papallacta 3300 m, sous bois, 01–7. 2001. P. Moret. Label 2: *Dyscolus silvestris* Moret HOLOTYPE. Label 3: *Dyscolus silvestris* Moret 2020, P. Moret det. 2020

Paratype QCAZI 260397. Label 1: Ecuador, Provincia Napo, Papallacta Termas Jamanco, 25–X–2015, WP41–3410 m, S 0°22'24.6"; W 78°10'05.5" Leaf litter at night/ P. Moret leg. Label 2: *Dyscolus silvestris* Moret 2020, PARATYPE

***Dyscolus sulcipedis* Moret & Murienne, 2020**

Holotype QCAZI 259397. Label 1: Ecuador, Loja, 4. VIII. 2016, PN Yacuri, WP 167–3240 m, 4.711861° S/79.440355° W, Leaf litter at night 19h–20h30; Moret, S. Aguirre, E. Moreno. Label 2: *Dyscolus sulcipedis* Moret HOLOTYPE. Label 3: *Dyscolus sulcipedis* Moret det 2019

***Dyscolus velox* Moret, 2005**

Paratype QCAZI 3199. Label 1: Ecuador, 3300–3600 m, 4–IV–1997, Laguna San Marcos; Leg. K. Los. Label 2: *Dyscolus velox* Moret PARATYPE. Label 3: *Dyscolus* *velox* Moret P. Moret det. 2016

Paratypes QCAZI 3200 and QCAZI 3201. Label 1: Ecuador, 3300–3600 m, 4–IV–1997, Laguna San Marcos; Leg. A. Jasinski. Label 2: *Dyscolus velox* Moret PARATYPE

***Hyboptera tiputini* Erwin & Henry, 2017**

Paratypes MEPN 38207 and MEPN 38208. Label 1: Ecuador, Orellana, Yasuni, Onkone Gare 216.3 m, 00°39'25,7" S, 76°27'10.8" W. Fumigación. Jun–1996 and Feb–1999 T. Erwin et al. lot. 1580 and lot. 2087. Label 2: Canopy Fogging Project Smithsonian Intitution, Fogging Station # 10, 22 June 1996 and # 8, 5 February 1999 T. L. Erwin el al. Colls. Label 3: PARATYPE, *Hyboptera tiputini* Erwin & Henry, Des. Terry L. Erwin, 2016

Paratype MEPN 39321. Label 1: ECUADOR: Orellana Pr. Tiputini Biodiversity Sta. Erwin–Harpia Plot t–2, 0.6332° S, 76.1443° W, 197 m, LOT # 1916. Label 2: Canopy Fogging Project Smithsonian Intitution, Fogging Station # 7, 23 October 1998, T. L. Erwin el al. Colls. Label 3: PARATYPE, *Hyboptera tiputini* Erwin & Henry, Des. Terry L. Erwin, 2017

Paratype MEPN 39322. Label 1: ECUADOR: Orellana Pr. Tiputini Biodiversity Sta. Erwin–Harpia Plot t–4, 0.6316° S, 76.1443° W, 208 m, LOT # 2033. Label 2: Canopy Fogging Project Smithsonian Institution, Fogging Station # 4, 8 February 1999, T. L. Erwin el al. Colls. Label 3: PARATYPE, *Hyboptera tiputini* Erwin & Henry, Des. Terry L. Erwin, 2017

Paratype MEPN 39323. Label 1: ECUADOR: Orellana Pr. Onkone Gare Station Erwin–Piraña Plot T–5, 0.6566° S, 76.4490° W, 220–250 m, LOT # 1565. Label 2: Canopy Fogging Project Smithsonian Institution, Fogging Station # 5, 22 June 1996, T. L. Erwin et al. Colls. Label 3: PARATYPE, *Hyboptera tiputini* Erwin & Henry, Des. Terry L. Erwin, 2017

***Hyboptera vestiverdis* Erwin & Henry, 2017**

Paratypes MEPN 39318, MEPN 39319 MEPN 39320. Label 1: ECUADOR: Orellana Pr. Onkone Gare Station Erwin–Piraña Plot T–4, T–6 and T–5, 0.6570° S, 76.4498° W, 220–250 m, LOT # 582, LOT# 1223 and LOT # 1199. Label 2: Canopy Fogging Project Smithsonian Intitution, Fogging Station # 3, 16 January 1994, 6 October 1995 and Fogging Station # 5, 6 October 1995. T. L. Erwin et al. Colls. Label 3: PARATYPE, *Hyboptera vestiverdis* Henry & Erwin, Des. Terry L. Erwin, 2017

***Loxandrus semperfidelis* Will, 2008**

Paratype **♀** QCAZI 2186. Label 1: 0°40'36" S; 76°24'2" W. Ecuador: Napo. Yasuni Scientific Station; 13. IV. 1998, Col. K. Will, 210 m. Headlamping. Label 2: UC. Label 3: Paratype, **♀** *Loxandrus semperfidelis* K. Will 2006

Patatype **♂** QCAZI 2187. Label 1: 0°40'36" S; 76°24'2" W. Ecuador: Napo Prov. Yasuni Scientific Station; 13: IV: 1998, 210 m, Col. K. Will, Headlamp. Label 2: *Loxandrus* un–jnterv. Rearing lot/chemistry lot. Label 3: U. C. Berkeley EMEC 1003649. Label 4: Paratype, *Loxandrus* **♂** *semperfidelis* K. Will 2006

***Moriosomus loebli* Allegro, Giachino & Picciau, 2018**

Paratype QCAZI 259339. Label 1: Ecuador, Cotopaxi, Otonga, 1800 m, río Esmeraldas, 3–5. VII. 01, P. Moret. Label 2: PARATYPUS *Moriosomus loebli* n. sp. Allegro, Giachino & Picciau det. 2018

***Tetracha onorei* Naviaux, 2007**

Allotype QCAZI 2038. Label 1: Ecuador, M. Santiago Río Cangaime, 219 m, 02°42'48" S, 77°29'56" W, 16 JAN. 2001 M. Vallejo R. Label 2: *Tetracha spixii*, (Brullé, 1837) Det. F. Cassola 2002. Label 3: ALLOTYPE, *Tetracha* (s. str.) *onorei* Naviaux, 2007

***Trechisibus barragani* Deuve & Moret, 2017**

Holotype QCAZI 224721. Label 1: Ecuador, Loja, 4. VIII. 2016, PN Yacuri WP 168–3445 m, 4,736713° S/79,427303° W, On open ground, P. Moret, S. Aguirre, E. Moreno. Label 2: HOLOTYPE. Label 3: *Trechisibus barragani* n. sp. Holotype, Th. Deuve & P. Moret det. 2017

***Trechisibus emiliae* Deuve & Moret, 2017**

Holotype QCAZI 224720. Label 1: Ecuador, Loja, 2. VIII. 2016, Cajanuma, Waypoint 166, 4116169° S/79,172046° W, Mossy bark and epiphytes fogging 2840 m, P. Moret, E. Moreno. Label 2: *Trechisibus emiliae* n. sp. Holotype, Th. Deuve & P. Moret det. 2017

***Trechisibus pubescens* Deuve & Moret, 2017**

Holotype QCAZI 224722. Label 1: Ecuador, Prov. Loja, Cordillera Lagunilla 14 S of Jimbura, 3510 m, S 04,74334, W 79,42442, 11. VIII. 2013, P. Moret. Label 2: *Trechisibus pubescens* n. sp. Holotype, Th. Deuve & P. Moret det. 2017

Paratype QCAZI 224723. Label 1: Ecuador, Loja, 4. VIII. 2016, PN Yacuri WP 169, 3150 m, 4,743257° S/79,424412° W, On open ground, P. Moret, S. Aguirre, E. Moreno. Label 2: PARATYPE. Label 3: *Trechisibus pubescens* n. sp. Th. Deuve & P. Moret det. 2017

**FAMILY CHRYSOMELIDAE**

***Beltia awapita* Flowers, 2018**

Holotype QCAZI 251844. Label 1: Ecuador, Esmeraldas, Playa de Oro, R. Santiago 00°53' N, 78°48' W, 200 m, 28 JUL–4 AGO 98, T. Enríquez. Label 2: *Beltia awapita* ns nombre Det. R. W. Flowers 2017. Label 3: HOLOTYPE

***Beltia ledesmae* Flowers, 2018**

Holotype QCAZI 251846. Label 1: Ecuador, Los Ríos, Quevedo, Est. Exp. Tropical Pichilingue Col. Internacional de Cacao, 24–mayo–2012 R. W. Flowers. Label 2: *Beltia ledesmae* ns. Nombre Det. R. W. Flowers 2018 HOLOTYPE

Allotype QCAZI 251847. Label 1: Ecuador, Los Ríos, Quevedo, Est. Exp. Tropical Pichilingue Sector Los Cauchos, Trampa Malaise, XII–2009/I–2010, R. W. Flowers. Label 2: *Beltia ledesmae* ns. Nombre Det. R. W. Flowers 2018 ALOTYPE

Paratypes QCAZI 251848, QCAZI 254282, QCAZI 254284 and QCAZI 254285. Label 1: same data as the holotype. Label 2: *Beltia ledesmae* ns. nombre Det. R. W. Flowers 2018 PARATYPE

Paratype QCAZI 251849. Label 1: same data as the holotype, except: 1–mayo–2014. Label 2: *Beltia ledesmae* ns. Nombre Det. R. W. Flowers 2018 PARATYPE

Paratype QCAZI 254281. Label 1: Ecuador, Pichincha, Pitzará, III–1992, Legt. G. Onore. Label 2: *Beltia ledesmae* ns. Nombre Det. R. W. Flowers 2018 PARATYPE

Paratype QCAZI 254283. Label 1: Ecuador, Los Ríos, Quevedo, Est. Exp. Tropical Pichilingue bosquecito, 2 Apr–2010 R. W. Flowers. Label 2: *Beltia ledesmae* ns. Nombre Det. R. W. Flowers 2018 PARATYPE

Paratype QCAZI 254286. Label 1: Ecuador, Los Ríos, E. C. Río Palenque 250 m, 79°33'00" W, 00°35'00" S, 20 FEB 1986, Santamaría. Label 2: *Beltia ledesmae* ns. Nombre Det. R. W. Flowers 2018 PARATYPE

Paratype QCAZI 254287. Label 1: same data as the holotype, except: 21–IV–2012. Label 2: *Beltia ledesmae* ns. Nombre Det. R. W. Flowers 2018 PARATYPE

***Beltia napoensis* Flowers, 2018**

Paratype QCAZI 254288. Label 1: Ecuador, Napo, Tarapoa Vía Cuyabeno, 9–X–1988, Legit P. Coral. Label 2: *Beltia napoensis* ns. Nombre Det. R. W. Flowers 2018 PARATYPE

Paratype QCAZI 254289. Label 1: Ecuador, Sucumbíos, SC. Station Yasuni 245 m, 30 AUG. 1995, X. Salazar. Label 2: *Beltia napoensis* ns. Nombre Det. R. W. Flowers 2018. PARATYPE

Paratype QCAZI 254290. Label 1: Ecuador, Sucumbíos, Cuyabeno, 830 m, 13–25 Jul 1993 T. Santander. Label 2: *Beltia napoensis* ns. Nombre Det. R. W. Flowers 2018 PARATYPE

Paratype QCAZI 254291. Label 1: Ecuador, Napo, Cuyabeno, Oct 18/85, Legit E. Carriazo. Label 2: *Beltia napoensis* ns. Nombre Det. R. W. Flowers 2018 PARATYPE

Paratype QCAZI 254292. Label 1: Ecuador, Sucumbíos, R. FP. Cuyabeno, 28/7/91 L. Schel. Label 2: *Beltia napoensis* ns. Nombre Det. R. W. Flowers 2018 PARATYPE

Paratype QCAZI 254293. Label 1: Ecuador, Oriente 00°24' S, 76°36' W, Limoncocha, Peter L. Kazan, 3 Ago 1970. Label 2: *Beltia napoensis* ns. Nombre Det. R. W. Flowers 2018 PARATYPE

Paratype QCAZI 254294. Label 1: Ecuador, Napo, Vía Hollin–Loreto Km 0, 1100 m, 6/12/97, M. Peñaherrera. Label 2: *Beltia napoensis* ns. Nombre Det. R. W. Flowers 2018 PARATYPE

Paratype QCAZI 254295. Label 1: Ecuador, Orellana, E. C. Yasuní km 40, 250 m, 76°28' W, 00°39' S, 17 AGO 1997, E. Baus. Label 2: *Beltia napoensis* ns. Nombre Det. R. W. Flowers 2018 PARATYPE

Paratype QCAZI 254296. Label 1: Ecuador, Orellana, E. C. Yasuní 250 m, 76°24'19" W, 00°40'32" S, 7 Oct 1997, E. Baus. Label 2: *Beltia napoensis* ns. Nombre Det. R. W. Flowers 2018. PARATYPE

Paratype QCAZI 254297. Label 1: Ecuador, Sucumbíos, Cuyabeno, 220 m, Lag. Grande 76°10' W, 00°01' N, 9–19 SEP 1996, X. Cisneros. Label 2: *Beltia napoensis* ns. Nombre Det. R. W. Flowers 2018. PARATYPE

Paratype QCAZI 254298. Label 1: Ecuador, Napo, Limon Coca 0°24' S, 76°46' W, Sep–Oct 1964, H. R. Hermann Jr. Label 2: *Beltia napoensis* ns. Nombre Det. R. W. Flowers 2018 PARATYPE

Paratype QCAZI 254299. Label 1: Ecuador, Sucumbíos, San Rafael Falls 1100 m, 5/6–VIII–98, W. Opitz. Label 2: *Beltia napoensis* ns. Nombre Det. R. W. Flowers 2018 PARATYPE

***Beltia talaga* Flowers, 2018**

Holotype QCAZI 251845. Label 1: Ecuador, Napo, Talag 600 m, 77°54' W, 01°03' S, 12 Jun 99, I. Oña. Label 2: *Beltia talaga* ns. Nombre Det. R. W. Flowers 2018 HOLOTYPE

***Elytromena constantini* Daccordi, 2008**

Paratypes QCAZI 2174 and QCAZI 2175. Label 1: Ecuador, prov. Azuay Gualaceo 20 km east páramos, 3°00' S; 78°39' W; 19. XI. 2006 and 28. XI. 2007b. R. Constantin. Label 2: Paratypus *Elytromelana constantini* n. sp. Det. M. Daccordi 2008

**FAMILY CURCULIONIDAE**

***Akrobothrus ecuadoriensis* Dole & Cognato, 2007**

Holotype MEPN 36942. Label 1: ECUADOR: Napo Prov. Res. Ethnica Waorani, 1 km S. Onkone Gare Camp. Trans. Ent. 26 January 1994, 220 m, 00°39'10'' S, 076°26' W. T. L. Erwin, et al. Insecticidal fogging, Terre Firme forest, Trans. 5, Sta. 4, Erwin–Lot # 653. Label 2: HOLOTYPE, *Akrobothrus ecuadoriensis* Dole and Cognato 2007

Allotype MEPN 37939. Label 1: same data as the Holotype, except: 2 October 1996, Lot # 1710. Label 2: ALLOTYPE, *Akrobothrus ecuadoriensis* Dole and Cognato 2007

***Camptocerus lucwildi* Smith & Cognato, 2017**

Paratype QCAZI 251014. Label 1: Ecuador, Los Ríos Prov. Canton Valencia, Reserva Murocumba -00,06389833; -79,1493167; 740 m, 24. iii. 2017, A. I. Cognato. Label 2: PARATYPE *Camptocerus lucwildi* Smith and Cognato

***Coptoburus ochromactonus*, Smith & Cognato, 2014**

Holotype QCAZI 2988. Label 1: Ecuador, Guayas Prov. El Empalme m, 514, 21. II. 2013. Y. Castro, ex. Cultivated balsa. Label 2: HOLOTYPE *Coptoborus ochromactonus* Smith & Cognato 2014

Paratype QCAZI 2989. Label 1: same data as the holotype. Label 2: PARATYPE *Coptoborus ochromactonus* Smith & Cognato 2014

Paratypes QCAZI 2990 and QCAZI 2991. Label 1: Ecuador, Cotopaxi Prov. Canton La Maná, Plantación Convenio Ochoa, 2. i. 2012, R. W. Flowers, M. Martínez ex. Dosel blasa. Label 2: PARATYPE *Coptoborus ochromactonus* Smith & Cognato 2014

Paratypes QCAZI 2992–QCAZI 2996. Label 1: Ecuador, S. D. de los Tsáchilas, Santo Domingo de los Colorados, Palmar del Bimbe, 398 m, L. Ortiz, ex boring into stem and xylem of Ochroma pyramidale. Label 2: PARATYPE *Coptoborus ochromactonus* Smith & Cognato 2014

Paratypes QCAZI 2997–QCAZI 3000. Label 1: Ecuador, Los Ríos, 23 km SW Quevedo, 2013, A. Stiwel. Label 2: PARATYPE *Coptoborus ochromactonus* Smith & Cognato 2014

***Coptonotus uteq* Smith & Cognato, 2016**

Paratype QCAZI 3446–QCAZI 3448. Label 1: Ecuador, Los Ríos, Cantón Valencia, Reserva Murucumba S 00°88544' 79 08 902' 731 m, 16. V. 2015. Cognato, Smith, Osborn, Martínez et al. Label 2: PARATYPE *Coptonotus uteq* Smith & Cognato 2016

Paratype QCAZI 3449. Label 1: Ecuador, Pastaza, Oglán 600 m, -1,3251, -77,68808, 05 Oct 2012, A. Pérez. Label 2: PARATYPE *Coptonotus uteq* Smith & Cognato 2016

***Howdeniola margheritae* Belló & Osella, 2008**

Paratype QCAZI 3173. Label 1: Ecuador, 00°25' S, 79°00' W, Cotopaxi, 1. III. 2003, M. Mora, Otonga, 2000 m. Label 2: *Howdeniola margheritae* sp. n. Paratypes det. Belló & Osella 2008

***Howdeniola onorei* Belló & Osella, 2008**

Paratypes QCAZI 3171 and QCAZI 3172. Label 1: Ecuador, Pichincha, S. José de Minas, 7–VIII–2006. Label 2: Cerro Blanco 3150 m. Label 3: N00°12.624" W078°21,050" vaglio pre–paramos. Label 4: leg. C. Belló, C. Osella & M. Pagliano. Label 5: Collezione Cesare Bello. Label 6: Paratypus Label 7: *Howdeniola margheritae* sp. n. Paratype det. Belló & Osella 2008

***Pandeleteius campbelli* Howden, 1976**

Paratypes QCAZI 2663. Label 1: Colom, Magd. 7000', San Lorenzo, 41 Km S, Sta. Marta, V–1–1973, Howden & Campbell. Label 2: Paratypus *Pandeletius campbelli* A. T. Howden

Paratype QCAZI 2664. Label 1: same label as the last, except: V–7–1973. Label 2: Paratypus *Pandeletius campbelli* A. T. Howden

**FAMILY ELATERIDAE**

***Paradrapetes serratus* Aranda, 1999**

Holotype QCAZI 2665. Label 1: Ecuador, Cotopaxi, Las Pampas, 1500 m, 3 APR 1996, G. Onore. Label 2: *Paradrapetes serratus* DET: Aranda. Label 3: HOLOTYPE

**FAMILY ELMIDAE**

***Cylloepus bartolozzii* Monte & Mascagni, 2012**

Paratypes QCAZI 2688, QCAZI 2689, QCAZI 2690. Label 1: PARATYPUS. Label 2: Ecuador: Napo, Yasuní Nat. Park, 300 m, 9–17. VIII. 2000, trap light, A. Sforzi, L. Bartolozzi leg. Label 3: CYLLOEPUS BARTOLOZZII n. sp. C. Monte & A. Mascagni Det. 2011

Paratypes QCAZI 2691–QCAZI 2694. Label 1: PARATYPUS. Label 2: Ecuador: Napo, Yasuní Nat. Park, 300 m, 9. VIII. 2000, trap light, A. Sforzi, L. Bartolozzi leg. Label 3: CYLLOEPUS BARTOLOZZII n. sp. C. Monte & A. Mascagni Det. 2011

Paratypes QCAZI 2695–QCAZI 2698. Label 1: PARATYPUS. Label 2: Ecuador: Napo, Yasuní National Park, PUCE Scientific Station, 300 m, 1. IV. 1997, G. Onore leg. Label 3: CYLLOEPUS BARTOLOZZII n. sp. C. Monte & A. Mascagni Det. 2011

***Cylloepus cesari* Monte & Mascagni, 2012**

Paratype QCAZI 2687. Label 1: PARATYPUS. Label 2: Ecuador: Cotopaxi, S. Francisco de Las Pampas, Bosque Integral Otonga, unnamed small stream in primary forest, 1500–1600 m, 26. VI. 2009. Label 3: F. Cianferoni, G. Mazza, C. Monte, M. Pazmiño, C. Tapia, F. Terzani legit. Label 4: CYLLOEPUS CESARI n. sp. C. Monte & A. Mascagni Det.

***Cylloepus fabianorum* Monte & Mascagni, 2012**

Paratype QCAZI 2700. Label 1: PARATYPUS. Label 2: Ecuador: Pichincha, Alluriquín, Otonga (Río Las Damas), 1400 m, 28. I. 2001, E. Tapia leg. Label 3: CYLLOEPUS FABIANORUM n. sp. C. Monte & A. Mascagni Det.

***Cylloepus francescae* Monte & Mascagni, 2012**

Paratype QCAZI 2699. Label 1: PARATYPUS. Label 2: Ecuador: Cotopaxi, S. Francisco de Las Pampas, Bosque Integral Otonga, unnamed small stream in primary forest, 1500–1600 m, 26. VI. 2009. Label 3: F. Cianferoni, G. Mazza, C. Monte, M. Pazmiño, C. Tapia, F. Terzani legit. Label 4: CYLLOEPUS FRANCESCAE n. sp. C. Monte & A. Mascagni Det. 2012

***Cylloepus mazzai* Monte & Mascagni, 2012**

Paratypes QCAZI 2702 and QCAZI 2705. Label 1: PARATYPUS. Label 2: Ecuador: Napo, Yasuní National Park, 300 m, 29. III. 1997, G. Onore leg. Label 3: CYLLOEPUS MAZZAI n. sp. C. Monte & A. Mascagni Det. 2012

Paratypes QCAZI 2703 and QCAZI 2704. Label 1: PARATYPUS. Label 2: Ecuador: Napo, Yasuní National Park, PUCE Scientific Station, 300 m, 1. IV. 1997, G. Onore leg. Label 3: CYLLOEPUS MAZZAI n. sp. C. Monte & A. Mascagni Det. 2012

***Cylloepus terzanii* Monte & Mascagni, 2012**

Paratype QCAZI 2674–QCAZI 2686. Label 1: PARATYPUS. Label 2: Ecuador: Cotopaxi, S. Francisco de Las Pampas, Bosque Integral Otonga, Río Esmeraldas, 1500–1600 m, 26. VI. 2009. Label 3: F. Cianferoni, G. Mazza, C. Monte, M. Pazmiño, C. Tapia, F. Terzani leg. Label 4: CYLLOEPUS TERZANII n. sp. C. Monte & A. Mascagni Det. 2011

***Macrelmis elicioi* Monte & Mascagni, 2012**

Paratype QCAZI 2701. Label 1: PARATYPUS. Label 2: Ecuador: Pichincha, Alluriquín, Otonga (Río Las Damas), 1400 m, 28. I. 2001, E. Tapia leg. Label 3: MACRELMIS ELICIOI n. sp. C. Monte & A. Mascagni Det. 2011

**FAMILY HYBOSORIDAE**

***Germarostes otonga* Ballerio & Gill, 2008**

Holotype **♂** QCAZI 2002. Label 1: Ecuador, Cotopaxi, Otonga, 2000 m, 00°25' LS, 79°00' LW, 25 APR 1999, T. Enríquez. Label 2: NTP Trap Human dung. Label 3: *Germarostes otonga* Ballerio & Gill 2008 Holotypus

Paratypes 6 **♂**, 8 **♀** QCAZI 1992–QCAZI 1997, QCAZI 2000 and QCAZI 2003–QCAZI 2008. Label 1: same data as the Holotype, and 25 MAR 19, 16 FEB 2000, 22 SEP 1999, 27 APR 1999, 29 APR 1999, 19 FEB 1999, 23 JUN 1999, 21 AGO 1999, 25 ABR 1999, 27 JUN 1999. T. Enríquez. Label 2: NTP Trap Human dung and NTP Trap with fish. Label 3: *Germarostes otonga* Ballerio & Gill 2008 Paratypus

Paratypes 1 **♂**, 1 **♀** QCAZI 1998 and QCAZI 1999. Label 1: Ecuador, Cotopaxi, Otonga, 2000 m, 00°25' LS, 79°00' LW, 18 MAY 1999, L. Torres & M. Thubert. Label 2: *Germarostes otonga* Ballerio & Gill 2008 Paratypus

Paratype **♀** QCAZI 2001. Label 1: Ecuador, Cotopaxi, Otonga (Monte bajo), 1800 m, 00°19'11" LS, 78°57'00" LW, I. G. Tapia, P. Ponce, 28 JUN 1997. Label 2: *Germarostes otonga* Ballerio & Gill 2008 Paratypus

**FAMILY LEIODIDAE**

***Adelopsis azuay* Salgado, 2013**

Holotype **♂** QCAZI 2672. Label 1: Ecuador, Azuay, 50 km NW Cuenca, 2470 m, 2. Jan 1992, C. Carlton, R. Leschen, # 94 ex: berlesale. Label 2: *Adelopsis azuay* n. sp. HOLOTYPE **♂**, Salgado det. (2013)

Paratype **♀** QCAZI 2671. Label 1: same data as the holotype. Label 2: *Adelopsis azuay* n. sp. PARATYPE, Salgado det. (2013)

***Adelopsis carolinae* Salgado, 2008**

Paratypes QCAZI 2148–QCAZI 2156. Label 1: Ecuador prov. Cotopaxi Cantón Sigchos, Las Pampas, Bosque Integral Otonga. Label 2: W79°00'204" S00°25'166", m 1975, 11. VII. 2007. A. C. Proaño & A. Barragán. Label 3: *Adelopsis carolinae* n. sp. PARATYPUS, Salgado det. (2008)

***Adelopspeleon acuminatum* Salgado, 2012**

Paratypes 4 **♂**, 1 **♀** QCAZI 2553–QCAZI 2555, QCAZI 2668 and QCAZI 2669. Label 1: Ecuador, Esmeraldas, La Y, Laguna de Cube 79°36' W, 00°23' N; 26 Aug 2005, I. Tapia, Ex: Cueva. Label: *Adelopspeleon acuminatum*, PARATYPE, Salgado det. 2012

***Dissochaetus angustilis* Salgado, 2010**

Paratypes 4 **♂**, 3 **♀** QCAZI 2624–QCAZI 2630. Label 1: Ecuador, Prov. de Pichincha, Unión de Toachi, reserva de Otongachi, 810 m, 10. III–5. IV. 2009, Salgado leg. Label 2: *Dissochaetus angustilis* PARATYPE Salgado det. (2010)

***Eucatops tungurahuaensis* Salgado, 2011**

Holotype **♀** QCAZI 2608. Label 1: Ecuador, Tungurahua, Río Machay, 1519 m, 17802564E; 9845094N, 10 MAR 2006, C. Carpio, ex: Tampa Pitfall cebo. Label 2: *Eucatops* (Eucatops) *tungurahuensis* n. sp. Holotype M, Salgado det. (2011)

***Ptomaphagus cubensis* Salgado, 2012**

Holotype **♂** QCAZI 2609. Label 1: Ecuador, Esmeraldas, La Y, Laguna de Cube 79°36' W, 00°23' N; 26 Aug. 2005, I. Tapia, Ex: Cueva. Label 2: *Ptomaphagus* (Adelops) *cubensis* n. sp. HOLOTYPE Salgado, 2012

Paratypes 41 **♂**, 27 **♀** QCAZI 2556–QCAZI 2607, QCAZI 2610–QCAZI 2623, QCAZI 2670 and QCAZI 2673. Label 1: same data as the Holotype. Label 2: *Ptomaphagus* (Adelops) *cubensis* n. sp. PARATYPE Salgado, 2012

**FAMILY LEPICERIDAE**

***Lepicerus pichilingue* Flowers, Shepard & Troya, 2010**

Holotype QCAZI 2189. Label 1: Ecuador: Los Ríos, Estación Experimental Tropical Pichilingue, 4 km SW Quevedo, Empalme Hwy. Lote La Teca, 25–IV–2008, leaf litter in plantain–cacao R. Troya. Label 2: HOLOTYPE *Lepicerus pichilingue* Flowers, Shepard & Troya

Paratype QCAZI 2190. Label 1: same data as the holotype. Label 2: PARATYPE *Lepicerus pichilingue* Flowers, Shepard & Troya

**FAMILY LUCANIDAE**

***Syndesus luki* Onore, Bartolozzi & Zilioli, 2011**

Paratypes QCAZI 2631–QCAZI 2633. Label 1: Ecuador, Pichincha, Tandayapa, 1650 m, 0,00594 -78,67455; 13 MAR 2010, G. Onore. Label 2: *Syndesus luki* Paratype G. Onore 2011

**FAMILY MELYRIDAE**

***Astylus moreti* Constantin, 2011**

Paratype QCAZI 2369. Label 1: Ecuador, Azuay, Cajas, XI–88, Legit: G. Onore. Label 2: PARATYPE. Label 3: *Astylus moreti* Constantin n. sp. R. Constantin det. 2011

***Melyrodes lojaensis* Constantin, 2008**

Paratype QCAZI 2017. Label 1: Ecuador, prov. Loja, Vilcabamba 30 km south forest, 8 km S Yangana 04°23' LS, 79°09' LW; 2122 m, 20. XI. 2007 C. R. Constantin. Label 2: Paratype *Melyrodes lojaensis* Constantin, 2008

**FAMILY NITIDULIDAE**

***Pocadius maquipucunensis* Leschen & Carlton, 1994**

Paratypes QCAZI 2666 and QCAZI 2667. Label 1: ECUADOR, Pichincha, Maquipucuna For. Res. 50 km NW Quito, 2600 m, 21 Dec. 1991, C. Carlton, R. Leschen ex: Lycopodium. Label 2: PARATYPE *Pocadius maquipucunensis* R. Leschen & C. Carlton.

**FAMILY PHENGODIDAE**

***Pseudophengodes onorei* Wittmer, 1996**

Paratype QCAZI 617 and QCAZI 618. Label 1: Ecuador, Imbabura, Chachimbiro, IX–1991, P. Ponce. Label 2: PARATYPUS. Label 3: *Pseudophengodes onorei* Wittm. Det. W. Wittmer

**FAMILY SCARABAEIDAE**

***Amithao cotopaxicus* Ratcliffe, 2017**

Paratypes QCAZI 224521, QCAZI 224531 and QCAZI 224529. Label 1: Ecuador, Cotopaxi, Las Pampas 1325 m, 18 Apr 1996, 21 Oct 1995, 20 Oct 1995, L. de la Torre. Label 2: *AMITHAO COTOPAXICUS* RATCLIFFE PARATYPE

Paratype QCAZI 224530. Label 1: Ecuador Cotopaxi, San Francisco de las Pampas 1500 m, 8–XII–1993, G. Onore. Label 2: *AMITHAO COTOPAXICUS* RATCLIFFE PARATYPE

Paratype QCAZI 254367. Label 1: Ecuador, Río Tocachi, Toachi, 19–IX–81, leg. G. Onore. Label 2: *AMITHAO COTOPAXICUS* RATCLIFFE PARATYPE

***Chrysina dzidorhum* (Arnaud, 1994)**

Paratype QCAZI 722. Label 1: Ecuador (CAN) Cochancai, 01. 02/91, P. Arnaud leg. Label 2: *Plusiotis dzidorhum* P. Arnaud det 94, PARATYPE

Paratype QCAZI 723. Label 1: Ecuador (CAN) Cochancai, 01. 03/92, P. Arnaud leg. Label 2: *Plusiotis dzidorhum* P. Arnaud det 94, PARATYPE

***Cyclocephala guaguarum* Dechambre & Endrödi, 1984**

Paratypes QCAZI 231234–QCAZI 231236. Label 1: Ecuador, S. Domingo, IV–1982; leg. G. Onore. Label 2: *Cyclocephala guaguarum* n. sp. PARATYPE, R. P. Dechambre det.

***Cyclocephala niguasa* Dechambre & Endrödi, 1984**

Paratypes QCAZI 235151, QCAZI 235155 and QCAZI 235156. Label 1: Ecuador, Los Ríos, Río Palenque; 22–V–1977. Label 2: *Cyclocephala niguasa* n. sp. PARATYPE

Paratypes QCAZI 235152 and 235153. Label 1: Ecuador, Sto. Domingo de los colorados; IX–82; G. Onore. Label 2: *Cyclocephala niguasa* n. sp. PARATYPE

***Eurysternus contractus* Génier, 2009**

Paratype QCAZI 2160. Label 1: Ecuador, Zamora Chinchipe, Vía Namirez-Zamora km 1, 1000 m, 26 August 1997, C. Carpio. Label 2: Paratype *Eurysternus contractus* sp. nov F. Génier

***Eurysternus lanuginosus* Génier, 2009**

Paratype QCAZI 2159. Label 1: Ecuador, Sucumbios, Lag. Zancudococha, 230 m, 15 Nov 1995, L. de la Torre. Label 2: Paratype *Eurysternus lanuginosus* sp. nov. F. Génier

***Gymnetis drogoni* Ratcliffe, 2018**

Paratype QCAZI 224522. Label 1: Ecuador, Manabí, Bosque Seco Lalo Loor, 42 m, 1–6 Ago 2009; F. Checa, W 80°09'05" S 00°04'57" Label 2: *GYMNETIS DROGONI* RATCLIFFE PARATYPE

***Gymnetis viserioni* Ratcliffe, 2018**

Paratype QCAZI 254366. Label 1: Ecuador, Cotopaxi, Las Pampas, IX–81; G. Onore. Label 2: *GYMNETIS VISERIONI* RATCLIFFE PARATYPE

***Odontolytes tectipennis* (Stebnicka & Skelley, 2005)**

Holotype MEPN 38134. Label 1: ECUADOR: NAPO, Res. Ethnica Waorani, 1 km S. Onkone Gare Camp. Trans. Ent. 20 jun 1996, 220 m, 00°39'10' S, 076°26' W. T. L. Erwin, et al. collectors. Label 2: Insecticidal fogging of mostly bare green leaves, some with covering of lichenous or bryophytic plants in terre firme forest. At Trans 2. Sta. 10 Project MAXUS Lot 1540. Label 3: HOLOTYPE, *Auperia tectipennis* Stebnicka & Skelley

Paratype MEPN 38135. Label 1: same data as the holotype, except 8–9 Feb 1995. Label 2: same data as the holotype. Label 3: PARATYPE, *Auperia tectipennis* Stebnicka & Skelley

Paratype MEPN 36951. Label 1: ECUADOR: Prov. Orellana, Tiputini Biodiversity Station 00°37'55 S, 076°08'39" W, 220–250 m, 4 July 1998 T. L. Erwin, et al. collectors. Label 2: Insecticidal fogging of mostly bare green leaves, some with covering of lichenous or bryophytic plants; Lot 1873 Transect # 8 Sta. 4. Label 3: PARATYPE, *Auperia tectipennis* Stebnicka & Skelley

***Odontolytes waoraniae* (Stebnicka & Skelley, 2005)**

Holotype MEPN 38252. Label 1: ECUADOR: NAPO, Res. Ethnica Waorani, 1 km S. Onkone Gare Camp. Trans. Ent. 5 feb 1996, 220 m, 00°39'10'' S, 076°26' W. T. L. Erwin, et al. collectors. Label 2: Insecticidal fogging of mostly bare green leaves, some with covering of lichenous or bryophytic plants in terre firme forest. At Trans 4. Sta. 9 Project MAXUS Lot 1439. Label 3: HOLOTYPE, *Auperia waoraniae* Stebnicka & Skelley

Paratypes MEPN 38246–38251, 38253, 38254, 38256, 38257 and 36950. Label 1: same data as the holotype, except 21 jun 1996, 3 jul 1995 and 8 feb 1995. Label 2: same data as the holotype except: Lot 1873 Transect # 8 Sta. 4, Lot 1459, 1099, 1439, 1097, 1559 and 951. Label 3: PARATYPE, *Auperia waoraniae* Stebnicka & Skelley

Paratype MEPN 38255. Label 1: ECUADOR: Prov. Orellana, Tiputini Biodiversity Station 00°37'55 S, 076°08'39" W 220–250 m, 5 Feb 1999 T. L. Erwin, et al. collectors. Label 2: Insecticidal fogging of mostly bare green leaves, some with covering of lichenous or bryophytic plants; Lot 2094 Trans. T/10 Sta. 5. Label 3: PARATYPE, *Auperia waoraniae* Stebnicka & Skelley

***Onorius inexpectatus* Frolov & Vaz de Mello, 2015**

Holotype QCAZI 3182. Label 1: Ecuador, Cotopaxi, Otonga, 1900 m, 3 FEB 1998, G. Onore. Label 2: Holotype *Onorius inexpectatus* Frolov & Vaz de Mello

Paratype QCAZI 3183. Label 1: Ecuador, Cotopaxi, Otonga; AUG. 1996, F. Rios. Label 2: PARATYPUS *Onorius inexpectatus* Frolov & V de Mello

Paratype QCAZI 3184. Label 1: Ecuador, Cotopaxi, Sigchos, Asache, 31 DEC 1995, E. Tapia. Label 2: PARATYPUS *Onorius inexpectatus* Frolov & V de Mello

Paratype QCAZI 3185. Label 1: same data as the holotype. Label 2: PARATYPUS *Onorius inexpectatus* Frolov & V de Mello

***Palaeophileurus silvestris* Neita & Ratcliffe, 2017**

Paratype QCAZI 224554. Label 1: Ecuador, Napo, II–85, S. Rafael, Legist: G. Onore. Label 2: *Palaeophileurus silvestris* Neita & Ratcliffe 2017 PARATYPE

***Phanaeus achilles lydiae* Arnaud, 2000**

Paratypes 2 **♂** 2 **♀** QCAZI 629–QCAZI 632. Label 1: Ecuador (GUA) Cerecita P. 02/87, P. Arnaud leg. Label 2: *Phanaeus achilles lydiae* P. Arnaud DET. PARATYPE 96

***Spodochlamys nazareti* Arnaud, 1995**

Paratypes QCAZI 2551 and QCAZI 2552. Label 1: Ecuador, Napo, Coordillera de los Guacamayos 1500 m, DEC 1993. Label 2: PARATYPE *Spodochlamys nazareti*

**FAMILY STAPHYLINIDAE**

***Gnathymenus rossii* Assing, 2013**

Paratype **♀** QCAZI 3006. Label 1: Ecuador, Cotopaxi, Bosque Integral Otonga, 10. VII. 2006, leg. W. Rossi. Label 2: Paratypus **♀** *Gnathymenus rossii* sp. n. det. V. Assing 2013

***Leptonia onorei* Pace, 2008**

Paratypes QCAZI 1838–QCAZI 1842, QCAZI 1869–QCAZI 1899, QCAZI 1906, QCAZI 1914–QCAZI 1918, QCAZI 1929–QCAZI 1933. Label 1: Ecuador, Napo, Vía Jondachi–Loreto km 59, ex cave, 700 m, 13. VIII. 2006. G. Onore leg. Label 2: *Leptonia onorei* n. sp. det. R. Pace 2008. Label 3: PARATYPUS *Leptonia onorei* mihi det. R. Pace 2008

Comments: several specimens mounted in the same pin

**ORDER DIPTERA**

**FAMILY ANTHOMYZIDAE**

***Mumetopia messor* Rohácek & Barber, 2008**

Paratype **♀** QCAZI 2135. Label 1: ECU: Carchi, Páramo El Angel, 17.3 km NW El Angel, litter under dead Espeletia, 31 Oct 1999, R. Anderson. Label 2: PARATYPE, *Mumetopia messor*, J. Rohácek & K. N. Barber det. 2008

Paratype **♀** QCAZI 2136. Label 1: ECU: Carchi, Páramo El Angel, 18.2 km NW El Angel, aspirated under dead Puya stems, 3 Nov 1999, S. A. Marshall. Label 2: PARATYPE, *Mumetopia messor*, J. Rohácek & K. N. Barber det. 2008

**FAMILY AULACIGASTRIDAE**

***Aulacigaster albifacies* Rung & Mathis, 2011**

Paratypes MEPN 37263 and MEPN 37264. Label 1: ECUADOR. Prt. Orellana: Rio Tiputini (0°38.2' S, 76°8.9' W) 12–26 Aug 1999, W. N. Mathis, A. Baptista, M. Kotrba. Label 2: Ceiba Lab. Label 3: *Aulacigaster albifacies*. Label 4: PARATYPE, *Aulacigaster albifacies* Rung & Mathis

***Aulacigaster formosa* Rung & Mathis, 2011**

Paratypes MEPN 37265 and MEPN 37266 **♂**. Label 1: ECUADOR. Prt. Orellana: Rio Tiputini (0°38.2' S, 76°8.9' W) 12–26 Aug 1999, W. N. Mathis, A. Baptista, M. Kotrba. Label 2: PARATYPE, *Aulacigaster formosa* Rung & Mathis

***Aulacigaster trifasciata* Rung & Mathis, 2011**

Paratypes MEPN 37270–MEPN 37272, MEPN 37279, MEPN 37280, MEPN 37282 **♀** MEPN 37284, MEPN 37354, MEPN 37355. Label 1: ECUADOR. Prt. Orellana: Rio Tiputini (0°38.2' S, 76°8.9' W) 12–26 Aug. 1999, W. N. Mathis, A. Baptista, M. Kotrba. Label 2: PARATYPE, *Aulacigaster trifasciata* Rung & Mathis

***Aulacigaster unifasciata* Rung & Mathis, 2011**

Paratype MEPN 37268. Label 1: ECUADOR. Orellana: Res. Etnica Waorani (0°39.4' S, 76°27.2' W; 216 m, lot # 577) 15 Jun 1994, T. L. Erwin. Label 2: PARATYPE, *Aulacigaster unifasciata* Rung & Mathis

***Aulacigaster vespertina* Rung & Mathis, 2011**

Paratype MEPN 37273 and MEPN 37274. Label 1: ECUADOR. Prt. Orellana: Rio Tiputini (0°38.2' S, 76°8.9' W) 12–26 Aug. 1999, W. N. Mathis, A. Baptista, M. Kotrba. Label 2: PARATYPE, *Aulacigaster vespertina* Rung & Mathis

**FAMILY CERATOPOGONIDAE**

***Forciponyia aidae* Hochman, Marino & Spinelli, 2017**

Holotype **♂** QCAZI 251042. Label 1: Ecuador, Imbabura, Chachimbiro 0°27'05,7" N, 78°13'44,9" W 2320 msnm, 10/11/–I–14. S. Hochman, Light trap F. (Metaf). Label 2: *Forciponyia* (Metaforcip.) *aidae* Hochman & Marino, Holotype **♂**

Allotype **♀** QCAZI 251041. Label 1: same data as the holotype. Label 2: *Forciponyia* (Metaforcip.) *aidae* Hochman & Marino, Allotype **♀**

***Forciponyia ivani* Hochman, Marino & Spinelli, 2017**

Holotype **♂** QCAZI 251043. Label 1: Ecuador, Imbabura, Chachimbiro 0°27'05,7" N, 78°13'44,9" W 2320 msnm, 10/11/–I–14. S. Hochman, Light trap F. (Lepidohelea) ivani. Label 2: *Forciponyia* (Lepidohelea) *ivani* Hochman & Marino, Holotype **♂**

Allotype **♀** QCAZI 251044. Label 1: same data as the Holotype. Label 2: *Forciponyia* (Lepidohelea) *ivani* Hochman, Marino & Spinelli 2017. Allotype **♀**

Paratypes 1 **♂**, 1 **♀** QCAZI 251045 and QCAZI 251046. Label 1: same data as the Holotype, except: *F.* (Lepidohelea) *ivani* H. & M. Paratype **♂** and **♀**. Label 2: *Forciponyia* (Lepidohelea) *ivani* Hochman, Marino & Spinelli 2017

Comments: Specimens of this family are preserved in glass slides

**FAMILY CLUSIDAE**

***Craspedochaeta argoniae* Lonsdale & Marshall, 2006**

Paratype **♂** QCAZI 2140. Label 1: ECU: Napo Province 7 km S Baeza, 2000 m, 25. II. 1979, H. & A. Howden, carrion trap. Label 2: PARATYPE, *Craspedochaeta argoniae* Lonsdale & Marshall

***Craspedochaeta pollostos* Lonsdale & Marshall, 2006**

Paratypes 2 **♂**, 1 **♀** QCAZI 2137, QCAZI 2138 and QCAZI 2139. Label 1: ECU: Pich. Pr. 250 m, 47 km S Sto. Domingo, Río Palenque Station, 17–25 II. 1979, S. A. Marshall. Label 2: PARATYPE, *Craspedochaeta pollostos* Lonsdale & Marshall

***Hendelia heliconiae* Lonsdale & Marshall, 2011**

Paratype 1 **♀** QCAZI 2141. Label 1: ECU: Pichincha, Nanegalito 7 km SE trout farm San José, 500 m, 30 Oct 1999, S. A. Marshall. Label 2: PARATYPE, *Hendelia heliconiae* Lonsdale & Marshall

Paratypes 2 **♀** QCAZI 2142 and QCAZI 2143. Label 1: ECU: Pichincha Maquipucuna Biol. Res. 1200 m, on leaves on small dung bait, 27 Oct 1999, S. A. Marshall. Label 2: PARATYPE, *Hendelia heliconiae* Lonsdale & Marshall

Paratypes 2 **♀** QCAZI 2144 and QCAZI 2145. Label 1: ECU: Pichincha Maquipucuna Biol. Res. river trail, 1200 m, 27 Oct 1999, S. A. Marshall. Label 2: PARATYPE, *Hendelia heliconiae* Lonsdale & Marshall

Paratype 2 **♀** QCAZI 2146 and QCAZI 2147. Label 1: ECU: Pichincha Maquipucuna Biol. Res. river trail, 1200 m, sweeping, 29 Oct 1999, S. A. Marshall. Label 2: PARATYPE, *Hendelia heliconiae* Lonsdale & Marshall

***Sobarocephala archisobarocephala* Lonsdale & Marshall, 2012**

Holotype **♂** QCAZI 2109. Label 1: ECU: Napo Prov. Puerto Misahualli, II–1983, 350 m, M. J. Sharkey. Label 2: HOLOTYPE *Sobarocephala archisobarocephala* Lonsdale & Marshall

***Sobarocephala bucki* Lonsdale & Marshall, 2012**

Holotype **♂** QCAZI 2111. Label 1: ECU: Napo, Jatun Sacha Res. 6 km E Misahualli, 450 m, 01°4' LS, 77°37' LW on foliage, 30 Apr–8 May 2002, M. Buck, debu00179887. Label 2: HOLOTYPE *Sobarocephala bucki* Lonsdale & Marshall

***Sobarocephala bulbosus* Lonsdale & Marshall, 2012**

Paratype **♀** QCAZI 2123. Label 1: ECU: Pichincha, Maquipucuna Biol. Res. 0°7'34" N, 78°37'57" W, on foliage, 25–28 Ago 2002, M. Buck. Label 2: PARATYPE *Sobarocephala bulbosus* Lonsdale & Marshall

***Sobarocephala dichotomos* Lonsdale & Marshall, 2012**

Paratype **♀** QCAZI 2119. Label 1: ECU: Pich. Pr. 47 km S Sto. Domingo Río Palenque Station, 17–25. II. 1979, S. A. Marshall. Label 2: PARATYPE *Sobarocephala dichotomos* Lonsdale & Marshall

***Sobarocephala echinata* Lonsdale & Marshall, 2012**

Paratype **♀** QCAZI 2124. Label 1: ECU: Pich. Pr. 47 km S Sto. Domingo Rio Palenque Station, 17–25. ii. 1979 S. X. Marshall. Label 2: PARATYPE, *Sobarocephala echinata* Lonsdale & Marshall

***Sobarocephala epeira* Lonsdale & Marshall, 2012**

Paratype **♂** QCAZI 2116. Label 1: ECU: Napo Jatun Sacha Res. 6 km E Misahualli varzea, sweep 01°4' LS, 77°37' LW, 3 May 2002, M. Buck debu 00201434. Label 2: PARATYPE, *Sobarocephala epeira* Lonsdale & Marshall

***Sobarocephala fuscina* Lonsdale & Marshall, 2012**

Paratype **♀** QCAZI 2125. Label 1: ECU: Napo Jatun Sacha Res. 6 km E Misahualli, 450 m, 01°4' LS, 77°37' LW varzea, 30 Apr–8 May 2002, S. A. Marshall, debu00196251. Label 2: PARATYPE *Sobarocephala fuscina* Lonsdale & Marshall

***Sobarocephala hispidifunda* Lonsdale & Marshall, 2012**

Paratype **♀** QCAZI 2118. Label 1: ECU: Napo prov. Puerto Misahualli, 350 m, II–1983, M. J. Sharkey. Label 2: PARATYPE *Sobarocephala hipidifuna* Lonsdale & Marshall

***Sobarocephala leptolineata* Lonsdale & Marshall, 2012**

Paratype **♀** QCAZI 2129. Label 1: ECU Napo, Tiputini Biodiv. St. vic. Yasuni Natl. Pk., 19 Feb 1998, D. C. Darling. Label 2: PARATYPE *Sobarocephala leptolineata* Lonsdale & Marshall

***Sobarocephala lita* Lonsdale & Marshall, 2012**

Paratype **♂** QCAZI 2126. Label 1: ECU: Pich. Pr. 47 km S Sto. Domingo Río Palenque Station, 17–25. II. 1979, S. A. Marshall. Label 2: PARATYPE *Sobarocephala lita* Lonsdale & Marshall

***Sobarocephala maquipucuna* Lonsdale & Marshall, 2012**

Holotype **♂** QCAZI 2112. Label 1: ECU: Pichincha, Maquipucuna Biol. Res. 0°7'34" N, 78°37'57" W on foliage, 26–28 Apr 2002, M. Buck. Label 2: HOLOTYPE *Sobarocephala maquipucuna* Lonsdale & Marshall

***Sobarocephala paieroi* Lonsdale & Marshall, 2012**

Holotype **♀** QCAZI 2115. Label 1: ECU: Napo Jatun Sacha Res. 6 km E Misahualli 01°4' LS, 77°37' LW, 30 Apr 2002, S. M. Paiero. Label 2: HOLOTYPE *Sobarocephala paieroi* Lonsdale & Marshall

***Sobarocephala patina* Lonsdale & Marshall, 2012**

Holotype **♂** QCAZI 2110. Label 1: ECU: Napo prov. 5 km N El Chaco, 15. II. 1983, M. J. Sharkey, malaise trap & wet net. Label 2: HOLOTYPE *Sobarocephala patina* Lonsdale & Marshall

***Sobarocephala pectinaria* Lonsdale & Marshall, 2012**

Paratype **♀** QCAZI 2120. Label 1: ECU Pich. Pr. 47 km S Sto. Domingo Rio Palenque Station, 17–25. ii. 1979, S. A. Marshall. Label 2: PARATYPE, *Sobarocephala pectinaria* Lonsdale & Marshall

***Sobarocephala sinuata* Lonsdale & Marshall, 201**2

Paratype **♀** QCAZI 2117. Label 1: ECU: Pichincha, Bellavista Reserve, 2200 m, 30 Oct 1999, S. A. Marshall, debu 00111911. Label 2: PARATYPE *Sobarocephala sinuata* Lonsdale & Marshall

***Sobarocephala spatulata* Lonsdale & Marshall, 2012**

Paratype **♀** QCAZI 2121 and **♀** QCAZI 2122. Label 1: ECU: Napo Jatun Sacha Res. 6 km E Misahualli 01°4' LS, 77°37' LW varzea dung pans, 2–7 May 2002, M. Buck, debu 00195868 and debu 00201283. Label 2: PARATYPE, *Sobarocephala spatulata* Lonsdale & Marshall

***Sobarocephala subtriangulina* Lonsdale & Marshall, 2012**

Holotype **♂** QCAZI 2114. Label 1: ECU: Napo Prov. Tena, ii. 1983, M. J. Sharkey. Label 2: HOLOTYPE *Sobarocephala subtriangulina* Lonsdale & Marshall

***Sobarocephala thrinax* Lonsdale & Marshall, 2012**

Paratype **♀** QCAZI 2127 and **♂** QCAZI 2128. Label 1: Bol: La Paz, Arroyo Tuhiri W Mapiri, 15°17'27" LS, 68°15'29" LW, 10 Apr 2001, S. A. Marshall, debu 00190236 and debu 00190464. Label 2: PARATYPE, *Sobarocephala thrinax* Lonsdale & Marshall

***Sobarocephala tinctoalata* Lonsdale & Marshall, 2012**

Holotype **♂** QCAZI 2113. Label 1: ECU: Napo Prov. Tena, ii. 1983, M. J. Sharkey. Label 2: HOLOTYPE, *Sobarocephala* *tinctoalata* Lonsdale & Marshall

**FAMILY CURTONOTIDAE**

***Curtonotum bivittatum* Klymko & Marshall, 2011**

Holotype **♂** QCAZI 2397. Label 1: Ecu: Napo, Jatun Sacha Res. 6 km E Misahuallí, 450 m, 1°4' S, 77°37' W. Sol Trail, 30 Apr–8 May 2002, S. A. Marshall. Label 2: HOLOTYPE, *Curtonotum biviittatum* Klymko & Marshall 2011

**FAMILY DROSOPHILIDAE**

***Drosophila anthurium* Llangarí & Rafael, 2020**

Holotype **♂** QCAZI 3099. Label 1: Ecuador, S. Tsáchilas, E. C. Río Guajalito 1800 m, 0°13'48 S, 78°49'16" W; Mar 2011, L. M. Llangarí. Label 2: *D*. *anthurium* Holotype Det. L. Llangarí 2013. Ex *Anthurium* spp.

Paratypes 9 **♂** QCAZI 3100–QCAZI 3108. Label 1: same data as the holotype, except: Jul 2011, Ago 2011, Jun 2011, Mar 2011. Label 2: *D.* *anthurium* Paratype Det. L. Llangarí 2013. Ex *Anthurium* spp.

***Drosophila ayauma* Peñafiel & Rafael, 2019**

Holotype **♂** QCAZI 3336. Label 1: Ecuador, Loja, Cajanuma, 2800 m, 4°6'58.9'' S; 79°10'11.9'' W; 19 Nov 2015; A. D. Peñafiel. Label 2: *D. ayauma* Holotype Det: A. Peñafiel 2015. Ex: banana y levadura

Paratypes 7 **♂** QCAZI 3337–QCAZI 3342. Label 1: same data as the Holotype. Label 2: *D. ayauma* Paratype Det: A. Peñafiel 2015. Ex: banana y levadura

Paratype QCAZI 3343. Label 1: Napo, Río Guango 00°32'14'' S, 77°57'13.4'' W; 19 Sep 2015; A. B. Manzano. Label 2: *D. ayauma* Paratype Det: A. Peñafiel 2015. Ex: banana y levadura

***Drosophila cajanuma* Peñafiel & Rafael, 2019**

Holotype **♂** QCAZI 3399. Label 1: Ecuador, Loja, Cajanuma, 4°6'58.9'' S; 79°10'11.9'' W, 2800 m, 19 Nov 2015, A. D. Peñafiel. Label 2: *D.* *cajanuma* Holotype **♂** Det: A. Peñafiel 2015; Ex: banana y levadura

Paratypes 10 **♂** QCAZI 3400, QCAZI 251806–QCAZI 251814. Label 1: same data as the holotype, except: Dic 2015, D. Encalada. Label 2: *D.* *cajanuma* Paratype Det: A. Peñafiel 2015; Ex: banana y levadura

Paratype **♂** QCAZI 251815. Label 1: Ecuador, Napo, Río Guango 00°32'14'' S, 77°57'13.4'' W, 2548 m, Sep 2015 A. B. Manzano. Label 2: *D.* *cajanuma* Paratype Det: A. Peñafiel 2015; Ex: banana y levadura

***Drosophila carchensis* Peñafiel & Rafael, 2018**

Holotype **♂** QCAZI 251017. Label 1: Ecuador, Carchi, Reserva Ecológica El Angel, 3762 m, 00°47’22.1” N, 077°54'03,2" W, A. Peñafiel, Jul 2016. Label 2: *D. carchensis* Holotype Det. A. Peñafiel ex banana y levadura

Allotype **♀** QCAZI 251018. Label 1: same data as the Holotype. Label 2: *D. carchensis* Alotipo Det. A. Peñafiel ex banana y levadura

Paratypes 3 **♂**, 8 **♀** QCAZI 251019 y QCAZI 251020, QCAZI 251026–QCAZI 251033, QCAZI 251036. Label 1: same data as the holotype. Label 2: *D. carchensis* Alotipo Det. A. Peñafiel ex banana y levadura

Paratypes 5 **♂** 1 **♀** QCAZI 251021–QCAZI 251025, QCAZI 251034 and QCAZI 251035. Label 1: Ecuador, Carchi, Juncal, 3370 m, 00°48’19.8’’ N; 078’00’10.8’’ W, Julio 2016, A. Peñafiel. Label 2: *D*. *carchensis* Alotipo Det. A. Peñafiel ex banana y levadura

***Drosophila cartucho* Llangarí & Rafael, 2020**

Holotype **♂** QCAZI 3119. Label 1: Ecuador, S. Tsáchilas, E. C. Río Guajalito 1800 m, 0°13'48” S 78°49'16" W; Jul 2011, L. M. Llangarí. Label 2: *D*. *cartucho* Holotype L. Llangarí 2013. Ex *Anthurium* spp.

Paratypes 9 **♂** QCAZI 3120–QCAZI 3128. Label 1: same data as the holotype, except Mar 2011. Label 2: *D*. *cartucho* Paratype L. Llangarí 2013. Ex *Anthurium* spp.

***Drosophila carvalhoi* Cabezas, Llangarí & Rafael, 2015**

Holotype **♂** QCAZI 2975. Label 1: Ecuador, S. Tsáchilas, E. C. Río Guajalito 1800 m, 0°13'48 S, 78°49'16" W; Ago 2011, L. M. Llangarí. Label 2: *D. carvalhoi* Holotype L. M. Llangarí 2013. Ex *Anthurium* spp.

Allotype **♀** QCAZI 2981. Label 1: same data as the holotype, except Jul 2011. Label 2: *D. carvalhoi* Alotipo L. M. Llangarí 2013. Ex *Anthurium* spp.

Paratypes 6 **♂**, 2 **♀** QCAZI 2976–QCAZI 2980, QCAZI 2982–QCAZI 2984. Label 1: same as the holotype, except Jul 2011. Label 2: *D. carvalhoi* Paratype L. M. Llangarí 2013. Ex *Anthurium* spp.

***Drosophila cashapamba* Céspedes & Rafael, 2012**

Holotype **♂** QCAZI 2349. Label 1: Ecuador, Pichincha, Cashapamba 78°25'51" W, 0°19'59,3 S; 2417 m, JUN 2010, M. L. Figuero. Label 2: *D. cashapamba* Holotype Det. V. Rafel. Ex banana y levadura

Allotype **♀** QCAZI 2348. Label 1: same data as the holotype. Label 2*: D. cashapamba* Alotipo Det. V. Rafel. Ex banana y levadura

Paratypes 12 **♂**, 7 **♀** QCAZI 2350–QCAZI 2368. Label 1: same data as the holotype. Label 2: *D*. *cashapamba* Paratype Det. V. Rafael. Ex banana y levadura

***Drosophila caxarumi* Peñafiel & Rafael, 2018**

Holotype **♂** QCAZI 3306. Label 1: Ecuador, Loja, Cajanuma 2675 m, 4°6'53.7'' S; 79°10'54.6'' W, Nov 2015, A. D. Peñafiel. Label 2: *D. caxarumi* Holotype Det: A. Peñafiel/2015. Ex: banana y levadura

Paratypes **♂** QCAZI 3307 and 3308. Label 1: Napo, Río Guango 00°32'14'' S, 77°57'13.4'' W, Sep 2015. A. B. Manzano. Label 2: *D. caxarumi* Paratype Det: A. Peñafiel/2015. Ex: banana y levadura

***Drosophila chichu* Peñafiel & Rafael, 2019**

Holotype **♂** QCAZI 3356. Label 1: Ecuador, Loja, Prov. Cajanuma, 2800 m, a. s. l. 4°6'58.9'' S; 79°10'11.9'' W; Nov 2015; A. D. Peñafiel. Label 2: *D. chichu* Holotype Det: A. Peñafiel 2015

Paratype **♂** QCAZI 3357. Label 1: same data as the holotype. Label 2: *D. chichu* Paratype Det: A. Peñafiel 2015

***Drosophila chorlavi* Céspedes & Rafael, 2012**

Holotype **♂** QCAZI 2316. Label 1: Ecuador, Imbabura, Ibarra 78°10'42" W, 0°21'30 S; 2200 m, JUN 2004; M. L. Figuero. Label 2: *D. chorlavi* Holotype Det. V. Rafael

Allotype **♀** QCAZI 2315. Label 1: same data as the holotype. Label 2: *D. chorlavi* Holotype Det. V. Rafael

Paratype 10 **♂**, 10 **♀** QCAZI 2317–QCAZI 2336. Label 1: same data as the holotype. Label 2: *D. chorlavi* Holotype Det. V. Rafael

***Drosophila condorhuachana* Céspedes & Rafael, 2012**

Holotype **♂** QCAZI 2337. Label 1: Ecuador, Pichincha, Cruz Loma 78°31'24" W, 0°11'21,8 S; JUL 2008; D. Céspedes. Label 2: Holotype *D. condorhuachana* D. Céspedes det

Paratypes **♂** QCAZI 2338–QCAZI 2347. Label 1: same data as the holotype. Label 2: Paratype *D. condorhuachana* D. Céspedes det

***Drosophila cosanga* Ramos & Rafael, 2017**

Holotype **♂** QCAZI 3013. Label 1: Ecuador, Napo, Cdlla. Guacamayos 2200 m, 0°37'8,7 S, 77°50'22,12" W, Feb 2012, E. L. Ramos. Label 2: *D*. *cosanga* Holotype E. L. Ramos 2014. Ex banana y levadura

***Drosophila cruzloma* Llangarí & Rafael, 2018**

Holotype **♂** QCAZI 3153. Label 1: Ecuador, Pichincha, Teleférico 3100 m, 0°1'19 S, 78°31'25" W; Mar 2014, M. L. Figuero. Label 2: *D. cruzloma* Holotype Det. L. Llangarí 2014. Ex banana y levadura

Allotype **♀** QCAZI 3157. Label 1: same data as the holotype. Label 2: *D. cruzloma* Alotipo Det. L. Llangarí 2014. Ex banana y levadura

Paratypes 3 **♂**, 4 **♀** QCAZI 3154–QCAZI 3156, QCAZI 3158–QCAZI 3161. Label 1: same data as the holotype. Label 2: *D. cruzloma* Paratype Det. L. Llangarí 2014. Ex banana y levadura

***Drosophila cuasmali* Peñafiel & Rafael, 2018**

Holotype **♂** QCAZI 251037. Label 1: Ecuador, Carchi, Reserva Ecológica El Angel, 3762 m, 00°47’22.1" N, 077°54'03,2" W, A. Peñafiel, Jul 2016. Label 2: *D. cuasmali* Holotype Det. A. Peñafiel 2018 ex banana y levadura

Paratype **♂** QCAZI 251038. Label 1: same data as the holotype. Label 2: *D. cuasmali* Paratype Det. A. Peñafiel 2018 ex banana y levadura

Paratype **♂** QCAZI 251039. Label 1: Ecuador, Napo, Río Guango, 2550 m, 00°32'14" S, 77°57'13,4" W, Oct 2017, A. B. Manzano. Label 2: *D. cuasmali* Paratype Det. A. Peñafiel 2018 ex banana y levadura

***Drosophila cumanda* Llangarí & Rafael, 2018**

Holotype **♂** QCAZI 3162. Label 1: Ecuador, Napo, Cumandá 1864 m, 0°27'13" S, 77°52'46" W; Mar 2014, M. L. Figuero. Label 2: *D.* *cumanda* Holotype Det. M. L. Llangarí 2014

Allotype **♀** QCAZI 3166. Label 1: same data as the holotype. Label 2: *D.* *cumanda* Alotipo Det. M. L. Llangarí 2014

Paratypes 3 **♂**, 3 **♀** QCAZI 3163–QCAZI 3165, QCAZI 3167–QCAZI 3169. Label 1: same data as the holotype. Label 2: *D.* *cumanda* Paratype Det. M. L. Llangarí 2014

***Drosophila cuyuja* Ramos & Rafael, 2015**

Holotype **♂** QCAZI 3003. Label 1: Ecuador, Napo, Papallacta 3362 m, 0°22'52,6" S, 78°09'44,4" W; Sep 2012 E. L. Ramos. Label 2: *D. cuyuja* Holotype Det. E. L. Ramos 2013

***Drosophila deloscolorados* Llangarí & Rafael, 2020**

Holotype **♂** QCAZI 3089. Label 1: Ecuador, S. Tsáchilas, E. C. Río Guajalito 1800 m, 0°13'48" S, 78°49'16" W; Mar 2011, L. M. Llangarí. Label 2: *D*. *deloscolorados* Holotype Det. L. Llangarí 2013. Ex *Anthurium* spp.

Paratypes 9 **♂** QCAZI QCAZI 3090–QCAZI 3098. Label 1: same data as the holotype, except Jun 2011 and Jul 2011. Label 2: *D*. *deloscolorados* Paratype Det. L. Llangarí 2013. Ex *Anthurium* spp.

***Drosophila guacamayos* Ramos & Rafael, 2017**

Holotype **♂** QCAZI 3018. Label 1: Ecuador, Napo, Cdlla. Guacamayos 2200 m, 0°37'8,7" S, 77°50'22,12" W; Feb 2012, E. L. Ramos. Label 2: *D. guacamayos* Holotype Det. E. L. Ramos 2014. Ex banana y levadura

Paratypes 7 **♂** QCAZI 3019–QCAZI 3025. Label 1: same data as the holotype. Label 2: *D. guacamayos* Paratype Det. E. L. Ramos 2014. Ex banana y levadura

***Drosophila guajalito* Llangarí & Rafael, 2020**

Holotype **♂** QCAZI 3109. Label 1: Ecuador, S. Tsáchilas, E. C. Río Guajalito 1800 m, 0°13'48" S, 78°49'16" W; Jul 2011, L. M. Llangarí. Label 2: *D. guajalito* Holotype Det. L. Llangarí 2013. Ex *Anthurium* spp.

Paratypes 9 **♂** QCAZI 3110–QCAZI 3118. Label 1: same data as the Holotype, with Sep 2011, Mar 2011, Jun 2011. Label 2: *D. guajalito* Paratype Det. L. Llangarí 2013. Ex *Anthurium* spp.

***Drosophila inti* Cabezas, Llangarí & Rafael, 2015**

Holotype **♂** QCAZI 2964. Label 1: Ecuador, Pichincha, Intillacta 1890 m, 0°03'01,6" N, 78°43'23,7" W; Feb 2011, M. B. Cabezas. Label 2: *D. inti* Holotype Det. M. B. Cabezas 2013. Ex banana y levadura

Paratypes 10 **♂** QCAZI 2965–QCAZI 2974. Label 1: same data as the Holotype. Label 2: *D. inti* Paratype Det. M. B. Cabezas 2013. Ex banana y levadura

***Drosophila intillacta* Cabezas & Rafael, 2013**

Holotype **♂** QCAZI 2771. Label 1: Ecuador, Pichincha, Intillacta 1890 m, 0°03'01,6" N, 78°43'23,7" W; Feb 2011, M. B. Cabezas. Label 2: *D.* *intillacta* Holotype Det. M. B. Cabezas 2013

Paratype **♂** QCAZI 2770. Label 1: same data as the holotype. Label 2: *D.* *intillacta* Paratype Det. M. B. Cabezas 2013

Paratypes 4 **♂** QCAZI 2772–QCAZI 2775. Label 1: Ecuador, Napo, Cordillera de los Guacamayos 1700 m, 00°39'33,8" S 077°47'22,4" W; Nov 2012 E. Ramos. Label 2: *D.* *intillacta* Paratype Det. Cabezas 2013

***Drosophila kasha* Peñafiel & Rafael, 2019**

Holotype **♂** QCAZI 3436. Label 1: Ecuador, Loja, Cajanuma, 4°6'58.9'' S, 79°10'11.9'' W, 2800 m, Nov 2015 A. D. Peñafiel. Label 2: *D. kasha* Holotype Det: A. Peñafiel 2015. Ex: banana y levadura

Paratypes 12 **♂** QCAZI 3437 –QCAZI 3439, QCAZI 251828–QCAZI 251836. Label 1: Napo, Río Guango 00°32'14'' S, 77°57'13.4'' W, 2548 m, Sep 2015 A. B. Manzano. Label 2: *D. kasha* Paratype Det: A. Peñafiel 2015. Ex: banana y levadura

Paratypes 2 **♂** QCAZI 251826 and QCAZI 251827. Label 1: same data as the holotype. Label 2: *D. kasha* Paratype Det: A. Peñafiel 2015. Ex: banana y levadura

***Drosophila kingmani* Peñafiel & Rafael, 2018**

Holotype **♂** QCAZI 3389. Label 1: Ecuador, Zamora Chinchipe, San Francisco, 2190 m, 3°59'16,7" S, 79°5'35" W, Nov 2015, A. D. Peñafiel col. Label 2: *D. kingmani* Holotype Det: A. Peñafiel/2015. Ex: banana y levadura

***Drosophila kurillakta* Peñafiel & Rafael, 2019**

Holotype **♂** QCAZI 3355. Label 1: Ecuador, Zamora Chinchipe Prov. San Francisco, 2190 m, a. s. l. 03°59'16,7" S, 79°05'35" W, Apr 2015, A. D. Peñafiel. Label 2: *D. kurillakta* Holotype Det: A. Peñafiel 2015. Ex: banana y levadura

***Drosophila machalilla* Acurio, Rafael, Céspedes & Ruiz, 2013**

Holotype **♂** QCAZI 2519. Label 1: Ecuador, Manabí, Playa San José 43 m, 80°49'14,6" W, 01°13'46,4" S; 10 DIC 2010; A. Acurio. Label 2: *Drosophila* *machalilla* Holotype A. Acurio. Det. Enero 2011

Allotype **♀** QCAZI 2534. Label 1: same data as the holotype. Label 2: *Drosophila* *machalilla* Alotipo A. Acurio. Det. Enero 2011

Paratypes 9 **♂**, 6 **♀** QCAZI 2520–QCAZI 2533, QCAZI 2535. Label 1: same data as the Holotype. Label 2: *Drosophila* *machalilla* Paratype A. Acurio. Det. Enero 2011

***Drosophila malacatus* Peñafiel & Rafael, 2018**

Holotype **♂** QCAZI 3382. Label 1: Ecuador, Loja, Cajanuma, 2800 m, 4°6'58.9'' S, 79°10'11.9'' W, 19 Nov 2015, A. Peñafiel col. Label 2: *D. malacatus* Holotype Det: A. Peñafiel/2015

***Drosophila millmasapa* Peñafiel & Rafael, 2018**

Holotype **♂** QCAZI 3390. Label 1: Ecuador, Zamora Chinchipe, San Francisco, 2190 m, 3°59'16,7" S, 79°5'35" W, 19 Nov 2015, A. D. Peñafiel. Label 2: *D. millmasapa* Holotype Det: A. Peñafiel 2015. Ex: banana y levadura

Paratype **♂** QCAZI 3191: Label 1: same data as the Holotype. Label 2: *D. millmasapa* Paratype Det: A. Peñafiel 2015. Ex: banana y levadura

***Drosophila misi* Peñafiel & Rafael, 2018**

Holotype **♂** QCAZI 3329. Label 1: Ecuador, Loja, Cajanuma, 2800 m, 4°6'58.9'' S, 79°10'11.9'' W; Nov 2015, A. D. Peñafiel. Label 2: *D. misi* Holotype Det: A. Peñafiel/2015. Ex: banana y levadura

Paratype **♂** QCAZI 3294. Label 1: Ecuador, Loja, Cajanuma 2675 m, 4°6'53.7'' S, 79°10'54.6'' W, Nov 2015, A. Peñafiel. Label 2: *D. misi* Paratype Det: A. Peñafiel/2015. Ex: banana y levadura

Paratypes 6 **♂** QCAZI 3330–QCAZI 3335. Label 1: same data as the holotype. Label 2: *D. misi* Paratype Det: A. Peñafiel/2015. Ex: banana y levadura

***Drosophila napoensis* Ramos & Rafael, 2015**

Holotype **♂** QCAZI 3004. Label 1: Ecuador, Napo, Papallacta 3362 m, 0°22'52,6" S, 78°09'44,4" W; Sep 2012 E. L. Ramos. Label 2: *D. napoensis* Holotype Det. E. L. Ramos 2013. Ex banana y levadura

Paratype **♂** QCAZI 3005. Label 1: same data as the holotype. Label 2: *D. napoensis* Paratype. Det. E. L. Ramos 2013. Ex banana y levadura

***Drosophila neoamaguana* Ramos & Rafael, 2017**

Holotype **♂** QCAZI 3041. Label 1: Ecuador, Napo, Cdla. Guacamayos 2200 m, 0°37'8,7" S, 77°50'22,12" W; May 2012; E. L. Ramos. Label 2: *D*. *neoamaguana* Holotype Det. E. L. Ramos 2014. Ex banana y levadura

Allotype **♀** QCAZI 3042. Label 1: same data as the holotype. Label 2: *D*. *neoamaguana* Alotipo Det. E. L. Ramos 2014. Ex banana y levadura

Paratypes 35 **♂** QCAZI 3043–QCAZI 3077. Label 1: same data as the holotype, except E. L. Ramos and Sep 2012, Jun 2014. Label 2: *D*. *neoamaguana* Paratype Det. E. L. Ramos 2014. Ex banana y levadura

***Drosophila neoasiri* Figuero & Rafael, 2013**

Holotype **♂** QCAZI 2715. Label 1: Ecuador, Pichincha, Páramo de Papallacta 4014 m, 78°12'32,1" W, 0°20'09,4" S; Ene 2006, V. Rafael. Label 2: *D. neoasiri* Holotype Det. M. L. Figuero 2012. Ex banana y levadura

Paratypes 8 **♂** QCAZI 2707–QCAZI 2714. Label 1: same data as the holotype. Label 2: *D. neoasiri* Holotype Det. M. L. Figuero 2012. Ex banana y levadura

***Drosophila neocapnoptera* Figuero & Rafael, 2013**

Holotype **♂** QCAZI 2725. Label 1: Ecuador, Napo, Cordillera Guacamayos 2200 m, 0°37'08,7" S, 77°50'21,2" W; Feb 2012; M. L. Figuero, D. Céspedes. Label 2: *D. neocapnoptera* Holotype Det. M. L. Figuero 2012. Ex banana y levadura

Allotype **♀** QCAZI 2719. Label 1: same data as the Holotype. Label 2: *D. neocapnoptera* Alotipo Det. M. L. Figuero 2012. Ex banana y levadura

Paratypes 5 **♂**, 3 **♀** QCAZI 2716–QCAZI 2718, QCAZI 2720–QCAZI 2724. Label 1: same data as the Holotype. Label 2: *D. neocapnoptera* Alotipo Det. M. L. Figuero 2012. Ex banana y levadura

***Drosophila neoprosaltans* Ramos & Rafael, 2017**

Holotype **♂** QCAZI 3026. Label 1: Ecuador, Napo, Cdlla. Guacamayos 2200 m, 0°37'8,7" S, 77°50'22,12" W; Feb 2012, E. L. Ramos. Label 2: *D*. *neoprosaltans* Holotype Det. E. L. Ramos 2014 ex banana y levadura

Allotype **♀** QCAZI 3027. Label 1: same data as the Holotype. Label 2: *D*. *neoprosaltans* Alotipo Det. E. L. Ramos 2014 ex banana y levadura

Paratypes 3 **♂**, 10 **♀** QCAZI 3028–QCAZI 3040. Label 1: same data as the Holotype. Label 2: *D*. *neoprosaltans* Paratype Det. E. L. Ramos 2014 ex banana y levadura

***Drosophila neoyanayuyu* Ramos & Rafael, 2017**

Holotype **♂** QCAZI 3010. Label 1: Ecuador, Napo, Papallacta 3362 m, 0°22'52,6" S, 78°09'44,4" W; Sep 2012 E. L. Ramos. Label 2: *D. neoyanayuyu* Holotype. Det. E. L. Ramos 2014. Ex banana y levadura

Allotype **♀** QCAZI 3009. Label 1: same data as the holotype. Label 2: *D. neoyanayuyu* Alotipo. Det. E. L. Ramos 2014. Ex banana y levadura

Paratypes 1 **♂**, 1 **♀** QCAZI 3011 and QCAZI 3012. Label 1: same data as the holotype. Label 2: *D. neoyanayuyu* Paratype. Det. E. L. Ramos 2014. Ex banana y levadura

***Drosophila nigua* Cabezas, Llangarí & Rafael, 2015**

Holotype **♂** QCAZI 2949. Label 1: Ecuador, Pichincha, Intillacta 1890 m, 0°03'01,6" N, 78°43'23,7" W; Feb 2011, M. B. Cabezas. Label 2: *D. nigua* Holotype Det. M. B. Cabezas 2013. Ex banana y levadura

Paratypes 5 **♂** QCAZI 2950–QCAZI 2952, QCAZI 2954 and QCAZI 2955. Label 1: same data as the holotype. Label 2: *D. nigua* Paratype Det. M. B. Cabezas 2013. Ex banana y levadura

Paratype **♂** QCAZI 2953. Label 1: Ecuador, S. Tsáchilas, E. C. Río Guajalito 1800 m, 0°13'48" S, 78°49'16" W; Jul 2011, M. B. Cabezas. Label 2: *D. nigua* Paratype Det. M. B. Cabezas 2013. Ex banana y levadura

***Drosophila nina* Cabezas & Rafael, 2015**

Holotype **♂** QCAZI 2782. Label 1: Ecuador, Pichincha, Intillacta 1890 m, 0°03'01,6" N, 78°43'23,7" W; Feb 2011, M. B. Cabezas. Label 2: *D. nina* Holotype Det. M. B. Cabezas 2012. Ex banana y levadura

Allotype **♀** QCAZI 2791. Label 1: same data as the holotype. Label 2: *D. nina* Alotipo Det. M. B. Cabezas 2012. Ex banana y levadura

Paratypes 8 **♂**, 9 **♀** QCAZI 2783–QCAZI 2790, QCAZI 2792–QCAZI 2800. Label 1: same data as the holotype. Label 2: *D. nina* Paratype Det. M. B. Cabezas 2012. Ex banana y levadura

***Drosophila papallacta* Figuero & Rafael, 2013**

Holotype **♂** QCAZI 2235. Label 1: Ecuador, Pichincha, Páramo de Papallacta 4014 m, 78°12'32,1" W, 0°20'09,4" S; ABR 2009, M. L. Figuero. Label 2: *D. papallacta* Holotype Det. M. L. Figuero 2009. Ex banana y levadura

Allotype **♀** QCAZI 2246. Label 1: same data as the holotype. Label 2: *D. papallacta* Alotipo Det. M. L. Figuero 2009. Ex banana y levadura

Paratypes 10 **♂**, 10 **♀** QCAZI 2236–QCAZI 2245, QCAZI 2247–QCAZI 2256. Label 1: same data as the holotype and JUL 2009, SEP 2009, FEB 2009. Label 2: *D. papallacta* Paratype Det. M. L. Figuero 2009. Ex banana y levadura

***Drosophila papaver* Tamayo & Rafael, 2016**

Holotype **♂** QCAZI 3176. Label 1: Ecuador, Pichincha, B. P. Yanacocha 3771 m, 0°7'16,1" S, 78°35'8,7" W; May 2014, M. I. Tamayo. Label 2: *D. papaver* Holotype Det. M. I. Tamayo 2014

Allotype **♀** QCAZI 3175. Label 1: same data as the holotype. Label 2: *D. papaver* Alotipo Det. M. I. Tamayo 2014

Paratypes 1 **♂**, 4 **♀** QCAZI 3177–QCAZI 3181. Label 1: same data as the Holotype. Label 2: *D. papaver* Paratype Det. M. I. Tamayo 2014

***Drosophila pappobolusae* Figuero, León, Rafael & Céspedes, 2012**

Holotype **♂** QCAZI 2497. Label 1: Ecuador, Pichincha, Quito, Parque Rumipamba 78°30'04,8" W, 00°10'49,9" S; 2903 m, MAY 2009, R. R. León. Label 2: *D.* *pappobolusae* Holotype Det. R. R. León 2009. Ex: *Pappobolus imbaburensis*

Allotype **♀** QCAZI 2508. Label 1: same data as the Holotype, except FEB 2009. Label 2: *D.* *pappobolusae* Alotipo Det. R. R. León 2009. Ex: *Pappobolus imbaburensis*

Paratypes 1 **♂**, 4 **♀** QCAZI 2498–QCAZI 2507, QCAZI 2509–QCAZI 2518. Label 1: same data as the holotype and JUL 2009 and DIC 2009. Label 2: *D.* *pappobolusae* Paratype Det. R. R. León 2009. Ex: *Pappobolus imbaburensis*

***Drosophila pichka* Peñafiel & Rafael, 2018**

Holotype **♂** QCAZI 3384. Label 1: Ecuador, Loja, Cajanuma, 2800 m, 4°6'58.9'' S; 79°10'11.9'' W, Nov 2015, A. D. Peñafiel. Label 2: *D. pichka* Holotype Det: A. Peñafiel 2015. Ex: banana y levadura

Paratype **♂** QCAZI 251243. Label 1: Ecuador, Napo, Río Guango, 2548 m, 00°32'14" S, 77°57'13,4" W, Sep 2016, A. B. Manzano. Label 2: *D. pichka* Paratype Det: A. Peñafiel/2015. Ex: banana y levadura

***Drosophila* *podocarpus* Peñafiel & Rafael, 2019**

Holotype **♂** QCAZI 3397. Label 1: Ecuador, Loja, Cajanuma, 4°6'58.9'' S; 79°10'11.9'' W, 2800 m, 19 Nov 2015 A. D. Peñafiel. Label 2: *D. podocarpus* Holotype Det: A. Peñafiel 2015; Ex: banana y levadura

Paratype **♂** QCAZI 3398. Label 1: same data as the holotype. Label 2: *D. podocarpus* Paratype; Det: A. Peñafiel 2015

***Drosophila pseudokorefae* Ramos & Rafael, 2018**

Holotype **♂** QCAZI 3016. Label 1: Ecuador, Napo, Papallacta 3362 m, 0°22'52,6" S, 78°09'44,4" W; FEB–08 SEP 2012; E. L. Ramos. Label 2: *D.* *pseudokorefae* Holotype Det. E. L. Ramos 2014

Paratype **♂** QCAZI 3017. Label 1: same data as the holotype. Label 2: *D. pseudokorefae* Paratype Det. M. L. Figuero 2009

***Drosophila pseudomorelia* Ramos & Rafael, 2018**

Holotype **♂** QCAZI 3014. Label 1: Ecuador, Napo, Papallacta 3362 m, 0°22'52,6" S, 78°09'44,4" W; FEB–11 NOV 2012; E. L. Ramos. Label 2: *D. pseudomorelia* Holotype Det. E. L. Ramos 2014 ex banana y levadura

Paratype **♂** QCAZI 3015. Label 1: same data as the Holotype. Label 2: *D. pseudomorelia* Holotype Det. E. L. Ramos 2014 ex banana y levadura

***Drosophila quijos* Ramos & Rafael, 2015**

Holotype **♂** QCAZI 3001. Label 1: Ecuador, Napo, Cord. Guacamayos 2200 m, 0°37'8,7" S, 77°50'22,12" W; Sep 2012, E. L. Ramos. Label 2: *D. quijos* Holotype Det. E. L. Ramos 2013. Ex banana y levadura

Paratype **♂** QCAZI 3002. Label 1: same data as the holotype except May 2012. L. Figuero. Label 2: *D. quijos* Holotype Det. E. L. Ramos 2013. Ex banana y levadura

***Drosophila quinarensis* Peñafiel & Rafael, 2018**

Holotype **♂** QCAZI 3286. Label 1: Ecuador, Loja, Cajanuma, 2800 m, 4°6'58.9'' S; 79°10'11.9'' W, Nov 2015, A. D. Peñafiel. Label 2: *D. quinarensis* Holotype Det: A. Peñafiel/2015. Ex: banana y levadura

Allotype **♀** QCAZI 3287. Label 1: same data as the holotype. Label 2: *D. quinarensis* Alotipo Det: A. Peñafiel/2015. Ex: banana y levadura

Paratypes 6 **♂**, 1 **♀** QCAZI 3288–QCAZI 3297. Label 1: same data as the holotype. Label 2: *D. quinarensis* Paratype Det: A. Peñafiel/2015. Ex: banana y levadura

Paratypes 3 **♀** QCAZI 3298–QCAZI 3300 Label 1: Ecuador, Loja, Cajanuma, 2675 m, 4°6'53.7'' S; 79°10'54,6'' W, 19 Nov 2015, A. Peñafiel. Label 2: *D. quinarensis* Paratype Det: A. Peñafiel/2015. Ex: banana y levadura

***Drosophila rucux* Céspedes & Rafael, 2012**

Holotype **♂** QCAZI 2305. Label 1: Ecuador, Pichincha, Cruz Loma 78°31'25,1" W, 0°11'19" S; 3325 m, ABR 2008, Céspedes D. Label 2: *D. rucux* Holotype Det. D. Céspedes 2008

Allotype **♀** QCAZI 2293. Label 1: same data as the Holotype. Label 2: *D. rucux* Alotipo Det. D. Céspedes 2008

Paratypes 10 **♂**, 10 **♀** QCAZI 2294–QCAZI 2304, QCAZI 2306–QCAZI 2314. Label 1: same data as the Holotype. Label 2: *D. rucux* Paratype Det. D. Céspedes 2008

***Drosophila rusaryu* Peñafiel & Rafael, 2018**

Holotype **♂** QCAZI 3383. Label 1: Ecuador, Loja, Cajanuma, 2675 m, 4°6'53,7" S, 79°10'54,6" W, 19 Nov 2015, A. D. Peñafiel. Label 2: *D. rusaryu* Holotype Det: A. Peñafiel 2015. Ex: banana y levadura

Paratype **♂** QCAZI 251242. Label 1: Ecuador, Loja, Cajanuma, 2725 m, 4°7'4,6'' S; 79°10'38,6'' W; Dic 2016, D. Encalada. Label 2: *D. rusaryu* Det: A. Peñafiel 2015. Ex: banana y levadura

***Drosophila sachapuyu* Peñafiel & Rafael, 2018**

Holotype **♂** QCAZI 3309. Label 1: Ecuador, Loja, Cajanuma, 2675 m, 4°6'53.7'' S; 79°10'54.6'' W; Apr 2015, A. D. Peñafiel. Label 2: *D. sachapuyu* Holotype Det: A. Peñafiel/2015. Ex: banana y levadura

Allotype **♀** QCAZI 3310. Label 1: same data as the Holotype. Label 2: *D. sachapuyu* Alotipo Det: A. Peñafiel/2015. Ex: banana y levadura

Paratypes 9 **♂**, 9 **♀** QCAZI 3311–QCAZI 3328. Label 1: same data as the Holotype. Label 2: *D. sachapuyu* Paratype Det: A. Peñafiel/2015. Ex: banana y levadura

***Drosophila sagittifolii* Llangarí & Rafael, 2017**

Holotype **♂** QCAZI 2757. Label 1: Ecuador, S. Tsáchilas, E. C. Río Guajalito 1800 m, 0°13'48" S, 78°49'16" W; Jun 2011, L. M. Llangarí. Label 2: *D.* *sagittifolii* Holotype Det. L. M. Llangarí 2013. Ex sagittifolium

Allotype **♀** QCAZI 2767. Label 1: same data as the Holotype. Label 2: *D.* *sagittifolii* Alotipo Det. L. M. Llangarí 2013. Ex sagittifolium

Paratype 10 **♂**, 10 **♀** QCAZI 2748–QCAZI 2756, QCAZI 2758–QCAZI 2766, QCAZI 2768, QCAZI 2769. Label 1: same data as the Holotype. Label 2: *D.* *sagittifolii* Paratype Det. L. M. Llangarí 2013. Ex sagittifolium

***Drosophila saraguru* Peñafiel & Rafael, 2019**

Holotype **♂** QCAZI 3358. Label 1: Ecuador, Loja, Prov. Cajanuma, 2675 m, a. s. l. 4°6'53,7'' S; 79°10'54,6'' W; Nov 2015; A. Peñafiel. Label 2: *D. saraguru* Holotype Det: A. Peñafiel 2015. Ex: banana y levadura

Paratype **♂** QCAZI 3359. Label 1: same data as the holotype. Label 2: *D. saraguru* Paratype Det: A. Peñafiel 2015. Ex: banana y levadura

***Drosophila shunku* Peñafiel & Rafael, 2018**

Holotype **♂** QCAZI 3360. Label 1: Ecuador, Loja, Cajanuma, 2675 m, 4°6'53.7'' S; 79°10'54.6'' W, 19 Nov 2015, A. Peñafiel. Label 2: *D. shunku* Holotype Det: A. Peñafiel 2015. Ex: banana y levadura

Allotype **♀** QCAZI 3361. Label 1: same data as the holotype. Label 2: *D. shunku* Alotipo Det: A. Peñafiel 2015. Ex: banana y levadura

Paratypes 9 **♂**, 9 **♀** QCAZI 3362–QCAZI 3379. Label 1: same data as the holotype. Label 2: *D. shunku* Paratype Det: A. Peñafiel 2015. Ex: banana y levadura

***Drosophila shunkuku* Peñafiel & Rafael, 2018**

Holotype **♂** QCAZI 3380. Label 1: Ecuador, Loja, Cajanuma 2800 m, 4°6'58.9'' S; 79°10'11.9'' W, 19 Nov 2015, A. D. Peñafiel. Label 2: *D. shunkuku* Holotype Det: A. Peñafiel 2015. Ex: banana y levadura

Allotype **♀** QCAZI 3381. Label 1: same data as the holotype. Label 2: *D. shunkuku* Alotipo Det: A. Peñafiel 2015. Ex: banana y levadura

Paratype **♂** QCAZI 251241. Label 1: Ecuador, Loja, Cajanuma 2900 m, 4°7'00,9'' S; 79°10'7.2'' W, Dic 2016, D. Encalada. Label 2: *D. shunkuku* Paratype Det: A. Peñafiel 2015. Ex: banana y levadura

***Drosophila sisapamba* Figuero, León, Rafael & Céspedes, 2012**

Holotype **♂** QCAZI 2543. Label 1: Ecuador, Pichincha, Quito, Parque Rumipamba 78°30'040.1" W, 00°10'52,5" S; 2903 m, FEB 2009, R. R. León. Label 2: *D. sisapamba* Det. R. R. Leon 2009 Holotype. Ex: flores

Paratypes 2 **♂** QCAZI 2544 and QCAZI 2545. Label 1: same data as the holotype. Label 2: *D. sisapamba* Det. R. R. León 2009 Paratype. Ex: flores

***Drosophila taki* Peñafiel & Rafael, 2018**

Holotype **♂** QCAZI 3385. Label 1: Ecuador, Loja, Cajanuma, 2800 m, 4°6'58.9'' S; 79°10'11.9'' W, Nov 2015, A. D Peñafiel. Label 2: *D. taki* Holotype Det: A. Peñafiel 2015. Ex: banana y levadura

Paratypes **♂** QCAZI 3386–QCAZI 3388. Label 1: same label as the holotype. Label 2: *D. taki* Paratype Det: A. Peñafiel 2015. Ex: banana y levadura

Paratypes **♂** QCAZI 251244–QCAZI 251247. Label 1: Ecuador, Napo, Río Guango, 2548 m, 00°32'14" S, 77°57'13,4" W, Sep 2016, A. B. Manzano. Label 2: *D. taki* Paratype Det: A. Peñafiel 2015. Ex: banana y levadura

***Drosophila tinalandia* Llangarí & Rafael, 2018**

Holotype **♂** QCAZI 3131. Label 1: Ecuador, S. Tsáchilas, Tinalandia 660 m, 0°18'34" S, 79°03'0" W; Jul 2013, V. Rafael. Label 2: *D. tinalandia* Holotype Det. L. Llangarí 2014. Ex *Xanthosoma* spp.

Allotype **♀** QCAZI 3130. Label 1: same data as the holotype. Label 2: *D. tinalandia* Alotipo Det. L. Llangarí 2014. Ex *Xanthosoma* spp.

Paratype 1 **♂**, 1 **♀** QCAZI 3129 and QCAZI 3132. Label 1: same data as the holotype. Label 2: *D. tinalandia* Paratype Det. L. Llangarí 2014. Ex *Xanthosoma* spp.

***Drosophila tsachila* Llangarí & Rafael, 2020**

Holotype **♂** QCAZI 3079. Label 1: Ecuador, S. Tsáchilas, E. C. Río Guajalito 1800 m, 0°13'48" S, 78°49'16" W; Jul 2011, L. M. Llangarí. Label 2: *D. tsachila* Holotype. Det. L. Llangarí 2013. Ex *Anthurium* spp.

Paratypes **♂** QCAZI 3080–QCAZI 3088. Label 1: same data as the holotype and Mar 2011, Sep 2011, Ago 2011. Label 2: *D. tsachila* Paratype. Det. L. Llangarí 2013. Ex *Anthurium* spp.

***Drosophila valenteae* Llangarí & Rafael, 2018**

Holotype **♂** QCAZI 3142. Label 1: Ecuador Pichincha, Cashapamba 2417 m, 0°19'59" S, 78°25'51" W; Jul 2013, V. Rafael. Label 2: *D.* *valenteae* Holotype Det. M. L. Llangarí 2014 Ex: banana y levadura

Allotype **♀** QCAZI 3152. Label 1: same data as the holotype. Label 2: *D.* *valenteae* Alotipo Det. M. L. Llangarí 2014 Ex: banana y levadura

Paratypes 9 **♂**, 9 **♀** QCAZI 3133–QCAZI 3141, QCAZI 3143–QCAZI 3151. Label 1: same data as the holotype. Label 2: *D.* *valenteae* Paratype Det. M. L. Llangarí 2014. Ex: banana y levadura

***Drosophila verbesinae* Figuero, León, Rafael & Céspedes, 2012**

Holotype **♂** QCAZI 2476. Label 1: Ecuador, Pichincha, Quito, Parque Rumipamba 78°30'04,8" W, 00°10'49,0" S; 2914 m, JUL 2009, R. R. León. Label 2: *D. verbesinae* Holotype Det. R. R. León 2009. Ex *Verbesina sodiroi*

Allotype **♀** QCAZI 2487. Label 1: same data as the holotype, except May 2009. Label 2: *D. verbesinae* Alotipo Det. R. R. León 2009. Ex *Verbesina sodiroi*

Paratypes 10 **♂**, 9 **♀** QCAZI 2477–QCAZI 2486, QCAZI 2488–QCAZI 2496. Label 1: same data as the holotype and FEB 2009, MAY 2009. Label 2: *D. verbesinae* Paratype Det. R. R. León 2009. Ex *Verbesina sodiroi*

***Drosophila wachi* Peñafiel & Rafael, 2019**

Holotype **♂** QCAZI 3392. Label 1: Ecuador, Loja, Cajanuma, 2800 m, 4°6'58.9'' S; 79°10'11.9'' W, Nov 2015, A. D. Peñafiel. Label 2: *D. wachi* Holotype Det: A. Peñafiel 2015; Ex: banana y levadura

Allotype **♀** QCAZI 3295. Label 1: Ecuador, Napo, Río Guango 00°32'14'' S, 77°57'13.4'' W, 2548 m, Sep 2016 A. D. Peñafiel. Label 2: *D. wachi* Alotipo Det: A. Peñafiel 2015; Ex: banana y levadura

Paratypes 2 **♂** QCAZI 3301 and QCAZI 3302. Label 1: Ecuador, Loja, Cajanuma, 2725 m, 4°7'4.6'' S; 79°10'38.6'' W, Dic 2015 D. Encalada. Label 2: *D. wachi* Paratype Det: A. Peñafiel 2015; Ex: banana y levadura

Paratypes 3 **♂** QCAZI 3393–QCAZI 3395. Label 1: Ecuador, Loja, Cajanuma, 2800 m, 4°6'58.9'' S; 79°10'11.9'' W, 19 Nov 2015, A. D. Peñafiel. Label 2: *D. wachi* Paratype Det: A. Peñafiel 2015; Ex: banana y levadura

Paratypes 3 **♂**, 9 **♀** QCAZI 3303–QCAZI 3305, QCAZI 3421, QCAZI 3422, QCAZI 251799–QCAZI 251805. Label 1: same label as the allotype. Label 2: *D. wachi* Paratype Det: A. Peñafiel 2015; Ex: banana y levadura

Paratype **♂** QCAZI 3396. Label 1: Ecuador, Napo, Río Guango 00°32'14'' S, 77°57'13.4'' W, 2548 m, 19 Sep 2015, A. B. Manzano. Label 2: *D. wachi* Paratype Det: A. Peñafiel 2015; Ex: banana y levadura

***Drosophila warmi* Peñafiel & Rafael, 2019**

Holotype **♀** QCAZI 3344. Label 1: Ecuador, Zamora Chinchipe Prov. Bombuscaro, 1000 m, 04°06'59,8" S, 78°58'04,9" W; Apr 2015; A. D. Peñafiel. Label 2: *D. warmi* Holotype Det: A. Peñafiel/2015. Ex: banana y levadura

Allotype **♂** QCAZI 3345. Label 1: same data as the holotype. Label 2: *D. warmi* Alotipo

Paratype 2 **♂**, 8 **♀** QCAZI 3346–QCAZI 3354. Label 1: same data as the Holotype. Label 2: *D. warmi* Paratype Det: A. Peñafiel/2015. Ex: banana y levadura

***Drosophila wayta* Figuero, León, Rafael & Céspedes, 2012**

Holotype **♂** QCAZI 2546. Label 1: Ecuador, Pichincha, Quito, Parque Rumipamba 78°30'04,8" W, 00°10'49,9" S; 2921 m, JUL 2009; R. R. León. Label 2: *D. wayta* Holotype Det. R. R. León 2009

Paratypes 4 **♂** QCAZI 2547–QCAZI 2550. Label 1: same data as the holotype and MAY 2009, FEB 2009. Label 2: *D. wayta* Paratype Det. R. R. León 2009

***Drosophila yambe* Cabezas, Llangarí & Rafael, 2015**

Holotype **♂** QCAZI 2956. Label 1: Ecuador, Pichincha, Intillacta 1890 m, 0°03'01,6" N, 78°43'23,7" W; Feb 2013, M. B. Cabezas. Label 2: *D. yambe* Holotype Det. M. B. Cabezas 2013. Ex banana y levadura

Paratypes 5 **♂** QCAZI 2957–QCAZI 2961. Label 1: same data as the holotype. Label 2: *D. yambe* Paratype Det. M. B. Cabezas 2013. Ex banana y levadura

Paratypes 2 **♂** QCAZI 2962 and QCAZI 2963. Label 1: Ecuador, S. Tsáchilas, E. C. Río Guajalito 1800 m, 0°13'48" S, 78°49'16" W; Jul 2011; M. B. Cabezas. Label 2: *D. yambe* Paratype Det. M. B. Cabezas 2013. Ex banana y levadura

***Drosophila yanacocha* Tamayo & Rafael, 2016**

Holotype **♂** QCAZI 3174. Label 1: Ecuador, Pichincha, B. P. Yanacocha 3587 m, 0°7'5,6" S, 78°35'10" W; Ago 2014 M. Tamayo. Label 2: *D. yanacocha* Holotype Det. M. I. Tamayo 2014

***Drosophila yanaurcus* Figuero, Rafael & Céspedes, 2012**

Holotype **♂** QCAZI 2191. Label 1: Ecuador, Pichincha, Páramo de Papallacta 4005 m, 78°12'42,8" W, 0°19'24,8" S; FEB 2009, M. L. Figuero. Label 2: *D. yanaurcus* Holotype Det. M. L. Figuero 2008

Allotype **♀** QCAZI 2202. Label 1: same data as the holotype except ABR. 2009. Label 2: *D. yanaurcus* Alotipo Det. M. L. Figuero 2008

Paratypes 5 **♂**, 2 **♀** QCAZI 2196–QCAZI 2198, QCAZI 2200, QCAZI 2201, QCAZI 2203, QCAZI 2207. Label 1: same data as the Holotype. Label 2: *D. yanaurcus* Paratype Det. M. L. Figuero 2008

Paratypes 4 **♂**, 5 **♀** QCAZI 2192–QCAZI 2195, QCAZI 2204, QCAZI 2205, QCAZI 2208, QCAZI 2210 and QCAZI 2211. Label 1: Ecuador, Pichincha, Páramo de Papallacta 4014 m, 78°12'32,1" W, 0°20'09,4" S; 4014 m, FEB 2009, ABR 2009, JUL 2009; M. L. Figuero. Label 2: *D. yanaurcus* Paratype Det. M. L. Figuero 2008

Paratypes 1 **♂**, 3 **♀** QCAZI 2199, QCAZI 2206, QCAZI 2209, QCAZI 2212. Label 1: Ecuador, Pichincha, Peñas Blancas 3731 m, 78°13'28,1" W, 0°19'13" S; FEB 2009 M. L. Figuero. Label 2: *D. yanaurcus* Paratype Det. M. L. Figuero 2008

***Drosophila yanayuyu* Céspedes & Rafael, 2012**

Holotype **♂** QCAZI 2283. Label 1: Ecuador, Pichincha, Cruz Loma 78°31'25,1" W, 0°11'19" S; 3325 m, ABR 2008, Céspedes D. Label 2: *D. yanayuyu* Holotype Det. D. Céspedes–2008. Ex banana y levadura

Allotype **♀** QCAZI 2284. Label 1: same data as the holotype. Label 2: *D. yanayuyu* Alotipo Det. D. Céspedes–2008. Ex banana y levadura

Paratypes 3 **♀** QCAZI 2285–QCAZI 2287. Label 1: same data as the holotype. Label 2: *D. yanayuyu* Paratype Det. D. Céspedes–2008. Ex banana y levadura

***Drosophila yurag* Figuero & Rafael, 2011**

Holotype **♂** QCAZI 2257. Label 1: Ecuador, Pichincha, Páramo de Papallacta 4014 m, 78°12'32,1" W, 0°20'09,4" S, Feb 2009, M. L. Figuero. Label 2: *D. yurag* Holotype Det. M. L. Figuero 2009

Allotype **♀** QCAZI 2268. Label 1: same data as the Holotype. Label 2: *D. yurag* Alotipo Det. M. L. Figuero 2009

Paratypes 10 **♂**, 10 **♀** QCAZI 2258–QCAZI 2267, QCAZI 2269–QCAZI 2278. Label 1: same data as the Holotype and SEP 2009, JUL 2009. Label 2: *D. yurag* Paratype Det. M. L. Figuero 2009

***Drosophila yuragshina* Figuero & Rafael, 2011**

Holotype **♂** QCAZI 2281. Label 1: Ecuador, Pichincha, Páramo de Papallacta 4014 m, 78°12'32,1" W, 0°20'09,4" S; ABR 2009, M. L. Figuero. Label 2: *D. yuragshina* Holotype Det. M. L. Figuero 2009 ex. Banana y levadura

Paratypes 3 **♂** QCAZI 2279, QCAZI 2280 and QCAZI 2282. Label 1: same data as the Holotype and FEB 2009. Label 2: *D. yuragshina* Paratype Det. M. L. Figuero 2009 ex. Banana y levadura

***Drosophila yuragyacum* Figuero, Rafael & Céspedes, 2012**

Holotype **♂** QCAZI 2292. Label 1: Ecuador, Pichincha, Cruz Loma 78°31'38,6" W, 0°11'15" S; 3550 m, JUL 2008; Céspedes D. Label 2: *D. yuragyacum* Holotype Det. D. Céspedes 2008

Allotype **♀** QCAZI 2288. Label 1: same data as the Holotype. Label 2: *D. yuragyacum* Alotipo Det. D. Céspedes 2008

Paratype **♂** QCAZI 2291. Label 1: same data as the Holotype. Label 2: *D. yuragyacum* Paratype Det. D. Céspedes 2008

***Drosophila zamorana* Peñafiel & Rafael, 2018**

Holotype **♂** QCAZI 3266. Label 1: Ecuador, Zamora Chinchipe, San Francisco, 2190 m, 3°59'16,7" S, 79°5'35" W, Abr. 2015, A. D. Peñafiel. Label 2: *D. zamorana* Holotype Det: A. Peñafiel/2015. Ex: banana y levadura

Allotype **♀** QCAZI 3267. Label 1: same data as the Holotype. Label 2: *D. zamorana* Alotipo Det: A. Peñafiel/2015. Ex: banana y levadura

Paratypes 9 **♂**, 9 **♀** QCAZI 3268–QCAZI 3285. Label 1: same data as the Holotype. Label 2: *D. zamorana* Paratype Det: A. Peñafiel/2015. Ex: banana y levadura

***Hirtodrosophila lojana* Peñafiel & Rafael, 2019**

Holotype **♂** QCAZI 3423. Label 1: Ecuador, Loja, Cajanuma, 4°6'58.9'' S; 79°10'11.9'' W, 2800 m, Nov 2015, A. D. Peñafiel. Label 2: *Hirtodrosophila* *lojana* Holotype Det: A. D. Peñafiel 2015; Ex: banana y levadura

Allotype **♀** QCAZI 3296. Label 1: Ecuador, Loja, Cajanuma, 4°6'53.7'' S; 79°10'54.6'' W, 2800 m, ABR 2015, A. D. Peñafiel. Label 2: Alotipo

Paratypes 6 **♂** QCAZI 3424–QCAZI 3429. Label 1: same data as the Holotype. Label 2: *Hirtodrosophila* *lojana* Paratype Det: A. D. Peñafiel 2015; Ex: banana y levadura

Paratypes 2 **♂** 3 **♀** QCAZI 3430–QCAZI 3434. Label 1: same data as the Allotype. Label 2: *Hirtodrosophila* *lojana* Paratype Det: A. D. Peñafiel 2015; Ex: banana y levadura

Paratypes 10 **♂** QCAZI 251816–QCAZI 251825. Label 1: Ecuador, Napo, Río Guango 00°32'14'' S, 77°57'13.4'' W, 2548 m, Sep 2015, A. B. Manzano. Label 2: *Hirtodrosophila* *lojana* Paratype Det: A. D. Peñafiel 2015; Ex: banana y levadura.

***Hirtodrosophila villonacu* Peñafiel & Rafael, 2019**

Holotype **♂** QCAZI 3435. Label 1: Ecuador, Loja, Cajanuma, 4°6'58.9'' S; 79°10'11.9'' W, 2800 m, Nov 2015, A. D. Peñafiel. Label 2: *Hirtodrosophila* *villonacu* Holotype Det: A. Peñafiel 2015; Ex: banana y levadura

**FAMILY HYBOTYDAE**

***Elaphropeza thoracica* Raffone, 2010**

Paratypes QCAZI 2644 and QCAZI 2645. Label 1: Ecuador, Pichincha, La Unión del Toachi, Otongachi Natural Reserva, 5–6 Jul 2006, W. Rossi. Label 2: PARATYPES Diptera Hybotyidae *Elaphropeza thoracica* Raffone 2010

**FAMILY MICROPEZIDAE**

***Cardiacephala aeruginosa* Ferro & Marshall, 2018**

Holotype **♀** QCAZI 260013. Label 1: ECU, Prov. Orellano, Yasuní Natl. Pk. Yasuní Research Stn. 0°40'50" S, 76°24'2" W, 250 m, 28 Apr–8 May 2009, S. A. Marshall. Label 2: HOLOTYPE *Cardiacephala aeruginosa* Ferro & Marshall 2017

***Cardiacephala aspera* Ferro & Marshall, 2018**

Holotype **♀** QCAZI 260011. Label 1: ECU, Prov. Orellano, Yasuní Natl. Pk. Yasuní Research Stn. 0°40'50" S, 76°24'2" W 250 m, 28 Apr–5 May 2009, S. A. Marshall. Label 2: HOLOTYPE **♀** *Cardiacephala aspera* Ferro & Marshall 2017

***Cardiacephala vitrata* Ferro & Marshall, 2018**

Holotype **♀** QCAZI 260012. Label 1: Ecuador, Puerto Orellana, Tiputini Biodiversity Station 0°38.2' S, 76°08.9' W, leg Kotrba, viii. 1999. Label 2: HOLOTYPE *Cardiacephala vitrata* Ferro & Marshall 2017

***Paragrallomya ecuadorensis* Ferro & Marshall, 2020**

Holotype **♀** QCAZI 260010. Label 1: ECU, Napo, Jatun Sacha Res. 6 km E Misahuallí 450 m, 1°4' S, 77°37' W varzea, 30 Abr–8 May 2009, S. A. Marshall. Label 2: HOLOTYPE H. *Paragrallomya ecuadorensis* Ferro & Marshall 2019

**FAMILY NERIIDAE**

***Longina anguliceps* Buck & Marshall, 2004**

Paratype QCAZI 2132. Label 1: Ecuador, Cotopaxi, Otonga, 2000 m, 00°25' S 79°00' W, 23 AUG. 1999, T. Enríquez. Label 2: PARATYPE, *Longina anguliceps*, Buck & Marshall 2004

***Longina semialba* Buck & Marshall, 2004**

Paratype QCAZI 2133. Label 1: Ecuador, Napo Las Palmas, 2850 m, 18–21 AUG. 1996, A. Paucar. Label 2: PARATYPE, *Longina semialba*, Buck & Marshall 2004

Paratype QCAZI 2134. Label 1: ECU: Prov. Napo, Cosanga, 4.2 km S, pipeline trail, 2150 m, 7 Nov 1999, S. A. Marshall. Label 2: PARATYPE, *Longina semialba*, Buck & Marshall 2004

**FAMILY PSYCHODIDAE**

***Sycorax wampukrum* Bravo & Salazar, 2009**

Holotype **♂** QCAZI 2022. Label 1: Ecuador, Morona Santiago, Quebrada del Río Napinaza 2.92665°S y 78,40701° W, 1010 msnm, Salazar–V. D. col. Label 2: Holotype, Sycorax wampukrum Bravo y Salazar

Paratypes 14 **♂**, 1 **♀** QCAZI 2023–QCAZI 2037. Label 1: same data as the Holotype. Label 2: Paratype, Sycorax wampukrum Bravo y Salazar

Comments: specimens preserved in slides

**FAMILY SPHAEROCERIDAE**

***Antrops anovariegatus* Kits & Marshall, 2013**

Holotype **♂** QCAZI 260070. Label 1: ECU, Napo, Lago Papallacta nr 3400 m, forest above lake pans/dung, 4–8 Nov 1999, S. A. Marshall. Label 2: HOLOTYPE *Antrops anovariegatus* Kits & Marshall 2012

Paratypes 2 **♀** QCAZI 260071 and QCAZI 260072. Label 1: same data as the Holotype. Label 2: PARATYPE *Antrops anovariegatus* Kits & Marshall 2012

***Antrops aurantifemur* Kits & Marshall, 2013**

Holotype **♂** QCAZI 259914. Label 1: Ecu, Napo, Lago Papallacta nr 3400 m, forest above lake pans/dung, 4–8 Nov 1999, S. A. Marshall. Label 2: HOLOTYPE *Antrops aurantifemur* Kits & Marshall 2012

Paratypes 9 **♂**, 10 **♀** QCAZI 259915–QCAZI 259933. Label 1: same data as the Holotype. Label 2: PARATYPE *Antrops aurantifemur* Kits & Marshall 2012

***Antrops baeza* Kits & Marshall, 2013**

Holotype **♂** QCAZI 260065. Label 1: ECU: Napo, 15 km NW Baeza, 2. iii. 1976, 2200 m, Dq Tp. Speck. Label 2: HOLOTYPE *Antrops baeza* Kits & Marshall 2012

***Antrops bellavista* Kits & Marshall, 2013**

Holotype **♂** QCAZI 260006. Label 1: ECU, Prov. Pichincha, Bellavista Reserve 2200 m, 28 Oct 1999, S. A. Marshall. Label 2: HOLOTYPE *Antrops bellavista* Kits & Marshall 2012

Paratypes **♂**, 2 **♀** QCAZI 26007–QCAZI 26009. Label 1: ECU, Pichincha, Bellavista Reserve, 2200 m, ridge trail, pans nr dung, 28–30 Oct 1999, S. A. Marshall. Label 2: PARATYPE *Antrops bellavista* Kits & Marshall 2012

***Antrops bucki* Kits & Marshall, 2013**

Holotype **♂** QCAZI 260005. Label 1: ECU, Napo, Pichincha, Papallacta pass 4200 m, 0°19'15S, 78°11'51 W, paramo, yellow pans, 29 Apr–11 May 2002, Marshall & Buck. Label 2: HOLOTYPE *Antrops bucki* Kits & Marshall 2012

***Antrops cotopaxi* Kits & Marshall, 2013**

Holotype **♂** QCAZI 259974. Label 1: ECU, Pichincha, Cotopaxi Natl. Pk. Quebrada Mishahuaico 3600 m, along stream, pan traps, 26 Oct–8 Nov 1999, S. A. Marshall. Label 2: HOLOTYPE, *Antrops cotopaxi* Kits & Marshall 2012

Paratypes 6 **♂** QCAZI 259975–QCAZI 259980. Label 1: same data as the Holotype. Label 2: PARATYPE, *Antrops cotopaxi* Kits & Marshall 2012

***Antrops diversipennis* Kits & Marshall, 2013**

Holotype **♂** QCAZI 259905. Label 1: Ecu, Napo, Lago Papallacta nr 3400 m, forest above lake pans/dung, 4–8 Nov 1999, S. A. Marshall. Label 2: HOLOTYPE, *Antrops* diversipennis Kits & Marshall 2012.

Paratypes 5 **♂**, 3 **♀** QCAZI 259906–QCAZI 259913. Label 1: same data as the Holotype. Label 2: PARATYPE, *Antrops diversipennis* Kits & Marshall 2012

***Antrops eurus* Kits & Marshall, 2013**

Holotype **♂** QCAZI 260067. Label 1: ECU, Napo, Sierra Azul Lodge 14 km W Cosanga 2200 m, forest sweep, 5 Nov 1999, S. A. Marshall. Label 2: HOLOTYPE *Antrops eurus* Kits & Marshall 2012

***Antrops fuliginosus* Kits & Marshall, 2013**

Holotype **♂** QCAZI 259934. Label 1: ECU, Napo, 4000 m, Quito–Baeza road Elfin for. Dung tp., 1. iii. 1979, S. A. Marshall. Label 2: HOLOTYPE *Antrops fuliginosus* Kits & Marshall 2012

Paratypes 7 **♂**, 8 **♀** QCAZI 259935–QCAZI 259949. Label 1: same data as the Holotype. Label 2: PARATYPE *Antrops fuliginosus* Kits & Marshall 2012

***Antrops guandera* Kits & Marshall, 2013**

Holotype **♂** QCAZI 259950. Label 1: ECU, Carchi, Guandera For. Res. 15 km E San Gabriel 3300 m, trail to station sweeping, 1 Nov 1999 S. A. Marshall. Label 2: HOLOTYPE *Antrops guandera* Kits & Marshall 2012

Paratypes 7 **♂**, 7 **♀** QCAZI 259951–QCAZI 259964. Label 1: same data as the Holotype. Label 2: PARATYPE *Antrops guandera* Kits & Marshall 2012

***Antrops papallacta* Kits & Marshall, 2013**

Holotype **♂** QCAZI 260031. Label 1: ECU: Napo, Lago Papallacta nr 3400 m, forest above lake pans/dung, 4–8 Nov 1999, S. A. Marshall. Label 2: HOLOTYPE *Antrops papallacta* Kits & Marshall 2018

Paratypes 2 **♂**, 3 **♀** QCAZI 260032–QCAZI 260034, QCAZI 260036 and QCAZI 260037. Label 1: same data as the Holotype. Label 2: PARATYPE *Antrops papallacta* Kits & Marshall 2018

Paratype **♀** QCAZI 260035. Label 1: ECU, Napo, Quito–Baeza pass, 1 km E 3950 m, forest edge, dung traps, 4–8 Nov 1999, S. A. Marshall. Label 2: PARATYPE *Antrops papallacta* Kits & Marshall 2018

***Antrops pecki* Kits & Marshall, 2013**

Holotype **♂** QCAZI 260038. Label 1: ECU, Napo Prov. Quito–Baeza rd. Elfin forest above thermal spgs. Papallacta 3200 m, 16–21. ii. 1983 L. Masner pan trap. Label 2: HOLOTYPE *Antrops pecki* Kits & Marshall 2018

Paratypes 5 **♂**, 5 **♀** QCAZI 260039–QCAZI 260048. Label 1: same data as the Holotype. Label 2: PARATYPE *Antrops pecki* Kits & Marshall 2018

***Antrops quadrilobus* Kits & Marshall, 2013**

Holotype **♂** QCAZI 260049. Label 1: ECU, Carchi, Bosque El Arrayán, 6 km E San Gabriel 2830 m, forest dung traps, 2–4 Nov 1999, S. A. Marshall. Label 2: HOLOTYPE *Antrops quadrilobus* Kits & Marshall

Paratypes 5 **♂**, 2 **♀** QCAZI 260050–QCAZI 260056. Label 1: same data as the Holotype. Label 2: PARATYPE *Antrops quadrilobus* Kits & Marshall

***Antrops sierrazulensis* Kits & Marshall, 2013**

Holotype **♂** QCAZI 260068. Label 1: ECU, Napo, Sierra Azul Res. 14 km W Cosanga, 2200 m, 0°40'55S, 77°56'9 W dung baits, 8–11 May 2002, M. Buck. Label 2: HOLOTYPE *Antrops sierrazulensis* Kits & Marshall 2013

Paratype **♀** QCAZI 260069. Label 1: same data as the Holotype. Label 2: PARATYPE *Antrops sierrazulensis* Kits & Marshall 2013

***Antrops tetrastichus* Kits & Marshall, 2013**

Holotype ♂ QCAZI 260014. Label 1: ECU, Quito–Baeza road Elfinfor. 1. iii. 1979, dung, S. A. Marshall. Label 2: HOLOTYPE, *Antrops tetrastichus* Kits & Marshall 2013

Paratypes 3 **♂**, 2 **♀** QCAZI 260015–QCAZI 260017, QCAZI 260021, QCAZI 260022. Label 1: same label as the Holotype. Label 2: PARATYPE, *Antrops tetrastichus* Kits & Marshall 2013

Paratypes 3 **♂**, 4 **♀** QCAZI 260018–QCAZI 260020, QCAZI 260023–QCAZI 260026. Label 1: Ecuador, Prov. Napo Quito–Baeza pass, 1 km E forest edge dung traps, 4–8 Nov 1999, S. A. Marshall. Label 2: PARATYPE, *Antrops tetrastichus* Kits & Marshall 2013

***Antrops variegatus* Kits & Marshall, 2013**

Holotype **♂** QCAZI 260027. Label 1: ECU, Pich. Prov. 35 km E Tandapi (Cornejo Astorga), 24. vi. 75, 9300' S & J. Peck. Label 2: HOLOTYPE *Antrops variegatus* Kits & Marshall 2013

Paratypes 2 **♂**, 1 **♀** QCAZI 260028–QCAZI 260030. Label 1: same data as the Holotype. Label 2: PARATYPE *Antrops variegatus* Kits & Marshall 2013

***Aptilotella angela* Luk & Marshall, 2014**

Holotype QCAZI 260004. Label 1: ECU, Carchi, Páramo El Angel 14.1 km NW El Angel 3450 m, mixed Polylepis litter, 2 Nov 1999, R. Anderson. Label 2: HOLOTYPE *Aptilotella angela* Luk & Marshall

***Aptilotella ebenea* Luk & Marshall, 2014**

Holotype QCAZI 260001. Label 1: ECU, Pichincha, Bellavista Reserve, 12 km S Nanegalito, 2150 m, cloud forest, leaf litter, 30 Oct 1999, R. Anderson. Label 2: HOLOTYPE *Aptilotella ebenea* Luk & Marshall

***Aptilotella gemmula* Luk & Marshall, 2014**

Holotype QCAZI 260002. Label 1: ECU, Pichincha, Bellavista Reserve, 12 km S Nanegalito 2150 m, cloud forest, ridge trail leaf litter, 28 Oct 1999, R. Anderson. Label 2: HOLOTYPE *Aptilotella gemmula* Luk & Marshall

***Aptilotella pichinchensis* Luk & Marshall, 2014**

Holotype QCAZI 260003. Label 1: ECU, Pichincha, Campamento Pichan 27.5 km NW Quito 3350 m, cloud forest leaf litter, 22 Oct 1999, R. Anderson. Label 2: HOLOTYPE *Aptilotella pichinchensis* Luk & Marshall

***Boreantrops auranticeps* Kits & Marshall, 2015**

Holotype **♂** QCAZI 260057. Label 1: ECU, Napo, Sierra Azul Res. 14 km W Cosanga 2200 m, 0°40'55S, 77°56'9 W dung baits 8–11 May 2002, M. Buck. Label 2: HOLOTYPE *Boreantrops auranticeps* Kits & Marshall 2012

Paratypes 3 **♀** QCAZI 260058–QCAZI 260060. Label 1: same data as the Holotype. Label 2: PARATYPE *Boreantrops auranticeps* Kits & Marshall 2012

Paratypes 2 **♀** QCAZI 260061 and QCAZI 260062. Label 1: ECU, Pichincha, Bellavista Reserve 2200 m, 30 Oct 1999, S. A. Marshall. Label 2: PARATYPE *Boreantrops auranticeps* Kits & Marshall 2012

***Boreantrops pollex* Kits & Marshall, 2015**

Holotype **♂** QCAZI 259965. Label 1: ECU, Baeza, 05. III. 1979, S. A. Marshall. Label 2: HOLOTYPE *Boreantrops pollex* Kits & Marshall 2012

Paratypes 8 **♀** QCAZI 259966–QCAZI 259973. Label 1: ECU, Baeza, 05. III. 1979, S. A. Marshall. Label 2: PARATYPE *Boreantrops pollex* Kits & Marshall 2012

***Boreantrops subemarginatus* Kits & Marshall, 2015**

Paratype **♀** QCAZI 260063. Label 1: ECU, Napo, El Chaco, 4.8 km W, 1750 m, 7 Nov 1999, S. A. Marshall. Label 2: PARATYPE *Boreantrops subemarginatus* Kits & Marshall 2012

Paratype **♀** QCAZI 260064. Label 1: ECU, Baeza, 05. III. 1979, S. A. Marshall. Label 2: PARATYPE *Boreantrops subemarginatus* Kits & Marshall 2012

***Bromeloecia abundantia* Yau & Marshall, 2018**

Holotype **♂** QCAZI 260171. Label 1: ECU, Río Palenque, 27. II. 1979, dung, S. A. Marshall. Label 1: Holotype *Bromeloecia abundantia* Yau & Marshall 2018

Paratypes 2 **♂**, 3 **♀** QCAZI 260156–QCAZI 260160. Label 1: same data as the Holotype. Label 2: Paratype *Bromeloecia abundantia* Yau & Marshall 2018

Paratypes 1 **♂**, 2 **♀** QCAZI 260183, QCAZI 260185 and QCAZI 260186. Label 1: ECU, Río Palenque, 26. ii. 1976, S. Peck, J. Glasser trap. Label 2: Paratype *Bromeloecia abundantia* Yau & Marshall 2018

Paratypes 9 **♂**, 2 **♀** QCAZI 260171–QCAZI 260181. Label 1: ECU, Guayas, 78 km N Santa Elena, 27 km S Puerto López 500 ft. Dung trap, 25–27 Jul 1976, S. Peck. Label 2: Paratype *Bromeloecia abundantia* Yau & Marshall 2018

Paratypes 3 **♂**, 2**♀** QCAZI 260161–QCAZI 260165. Label 1: ECU, Napo, Tipunini Biodiversity Stn. vic. Yasuni Natl. Pk. 0°38' S, 76°10' W pitfall trap (human dung), 14–19 Feb 1998, D.C. Darling. Label 2: Paratype *Bromeloecia abundantia* Yau & Marshall 2018.

Paratypes 3 **♂**, 2 **♀** QCAZI 2600166–QCAZI 260170. Label 1: ECU, Napo, Jatun Sacha Res. 6 km E Misahuallí, 450 m, 1°4' S 77°37' W varzea, dung pans, 2–7 May 2002, M. Buck. Label 2: Paratype *Bromeloecia abundantia* Yau & Marshall 2018

***Bromeloecia aculatus* Yau & Marshall, 2018**

Paratypes 7 **♂**, 5 **♀** QCAZI 260113–QCAZI 260119, QCAZI 260122–QCAZI 260126. Label 1: ECU, Pich. 5200' 28 km E Alluriquín Chiriboga rd. 19–27. vi. 75, S. Peck, Moss For. Carr. Tp. Label 2: Paratype, *Bromeloecia aculatus* Yau & Marshall 2018

***Bromeloecia aurita* Yau & Marshall, 2018**

Holotype **♂** QCAZI 260331. Label 1: ECU: Napo, 17 km NE Baeza, 3–6. III. 1976, 1400 m, Dp Tp. S. Peck. Label 2: Holotype, *Bromeloecia aurita* Yau & Marshall 2018

Paratypes 14 **♂**, 16 **♀** QCAZI 260332–QCAZI 260361. Label 1: same data as the Holotype. Label 2: Paratype, *Bromeloecia aurita* Yau & Marshall 2018

***Bromeloecia balaena* Yau & Marshall, 2018**

Paratypes 13 **♂**, 8 **♀** QCAZI 260073–260093. Label 1: ECU, Pich. 8000' 22.2 km E Tandapi, 24–29 iv–75, S. Peck, Dung trap mass for. Label 2: Paratype *Bromeloecia balaena* Yau & Marshall 2018

***Bromeloecia brachium* Yau & Marshall, 2018**

Holotype **♂** QCAZI 260094. Label 1: ECU, Napo, Jatun Sacha Res., 6 km E Misahuallí, 450 m, 1°4' S, 77°37' W compost yellow pans, 1–2 May 2002, M. Buck. Label 2: Holotype, *Bromeloecia brachium* Yau & Marshall 2018

Paratypes 13 **♂**, 6 **♀** QCAZI 260095–260100, 260311–260323. Label 1: ECU, Esmeraldas, 11 km SE San Lorenzo, La Chiquita 5 m, 9–10 vi. 75, S. Peck, carrion and 10–11. vi. 1975, dung tp. Label 2: Paratype, *Bromeloecia brachium* Yau & Marshall 2018

***Bromeloecia cercarcuata* Yau & Marshall, 2018**

Holotype **♂** QCAZI 260254. Label 1: ECU, Napo, Tena 12 km SW 500 m, Peck dung trap 30–33 day 2–5, 8–11. vii. 1976. Label 2: Holotype, *Bromeloecia cercarcuata* Yau & Marshall 2018

Paratypes 5 **♂**, 5 **♀** QCAZI 260255–QCAZI 260264. Label 1: same data as the Holotype. Label 2: Paratype, *Bromeloecia cercarcuata* Yau & Marshall 2018

***Bromeloecia coniclunis* Yau & Marshall, 2018**

Paratypes 16 **♂**, 18 **♀** QCAZI 260214–QCAZI 260247. Label 1: ECU, Pich. 5200' 28 km E Alluriquín Chiriboga rd. 19–27. vi. 75, Peck, Moss For. Carr. Tp. Label 2: Paratype *Bromeloecia conclunis* Yau & Marshall 2018

Paratypes 4 **♂**, 2 **♀** QCAZI 260248–QCAZI 260253. Label 1: ECU, Pich. 8000' 22.2 km E Tandapi. 14–29. vi. 1975, Peck Dung trap mass for. Label 2: Paratype *Bromeloecia conclunis* Yau & Marshall 2018

***Bromeloecia pinna* Yau & Marshall, 2018**

Holotype **♂** QCAZI 260182. Label 1: ECU, Baeza, 5. iii. 1979, S. A. Marshall. Label 2: Holotype *Bromeloecia pinna* Yau & Marshall 2018

Paratype 1 **♀** QCAZI 260184. Label 1: same data as the Holotype. Label 2: Paratype *Bromeloecia pinna* Yau & Marshall 2018

Paratypes 4 **♂**, 4 **♀** QCAZI 260187–QCAZI 260194. Label 1: ECU, Napo, 15 km NW Baeza, 2–6 iii. 1976, 2200 m, S. Peck. Label 2: Paratype *Bromeloecia pinna* Yau & Marshall 2018

Paratypes 3 **♂**, 2 **♀** QCAZI 260195–QCAZI 260199. Label 1: ECU, Napo, Baeza, 17 km NE, 1400 m, carrion trap, 3–6. Mar 1976, S. Peck. Label 2: Paratype *Bromeloecia pinna* Yau & Marshall 2018

***Bromeloecia ponsa* Yau & Marshall, 2018**

Holotype **♂** QCAZI 260200. Label 1: Ecu, Napo, Lago Papallacta nr 3400 m, forest above lake pans/ dung, 4–8 Nov 1999, S. A. Marshall. Label 2: Holotype, *Bromeloecia ponsa* Yau & Marshall 2018

Paratypes 4 **♂**, 6 **♀** QCAZI 260204–QCAZI 260213. Label 1: ECU, Napo, 27 km NE Baeza 2700 m, 2–6 III. 1976, Speck. Label 2: Paratype, *Bromeloecia ponsa* Yau & Marshall 2018

Paratypes 1 **♂**, 2 **♀** QCAZI 260201–QCAZI 260203. Label 1: ECU, Napo, Baeza, 42 km NW 3300 m, dung trap, 2–6 May 1976 S. Peck. Label 2: Paratype, *Bromeloecia ponsa* Yau & Marshall 2018

***Bromeloecia ramus* Yau & Marshall, 2018**

Holotype **♂** QCAZI 260265. Label 1: ECU, Napo, Tiputini Biodiversity Stn. 0°36'50" S, 76°9'1" W; May 2011, S. A. Marshall. Label 2: Holotype, *Bromeloecia ramus* Yau & Marshall 2018

Paratypes 2 **♂**, 2**♀** QCAZI 260266–QCAZI 260269. Label 1: ECU, Napo, Tipunini Biodiversity Stn. Vic. Yasuni Natl. Pk. 0°38' S, 76°10' W pitfall trap (human dung), 19 Feb 1998, D. C. Darling. Label 2: Paratype, *Bromeloecia ramus* Yau & Marshall 2018.

***Bromeloecia robustora* Yau & Marshall, 2018**

Holotype **♂** QCAZI 260362. Label 1: ECU, Napo, Jatun Sacha Res. 6 km E Misahuallí 450 m, 1°4' S, 77°37' W varzea dung pans, 1–2 May 2002, Buck & Lonsdale. Label 2: Holotype *Bromeloecia robustora* Yau & Marshall 2018

Paratypes 12 **♂**, 18 **♀** QCAZI 260363–QCAZI 260392. Label 1: same data as the Holotype. Label 2: Paratype *Bromeloecia robustora* Yau & Marshall 2018.

***Bromeloecia triunguia* Yau & Marshall, 2018**

Holotype **♂** QCAZI 260140. Label 1: ECU, Río Palenque, 27. 11. 1979, S. A. Marshall. Label 2: Holotype *Bromeloecia triunguia* Yau & Marshall 2018

Paratypes 6 **♂**, 8 **♀** QCAZI 260141–QCAZI 260154. Label 1: ECU, Río Palenque, 25. II. 1979, dung, S. A. Marshall. Label 2: Paratype *Bromeloecia triunguia* Yau & Marshall 2018

***Bromeloecia undulata* Yau & Marshall, 2018**

Holotype **♂** QCAZI 260270. Label 1: ECU, Esmeraldas, 11 km SE San Lorenzo, La Chiquita 5 m, 10–11. vi. 1975, Dung Tp. S. Peck. Label 2: Holotype *Bromeloecia undulata* Yau & Marshall 2018

Paratypes 14 **♂**, 19 **♀** QCAZI 260271–QCAZI 260303. Label 1: same data as the Holotype and 9–10. vi. 75, 7–8. vi. 1975, 6–7 Jun 1975. Label 2: Paratype *Bromeloecia undulata* Yau & Marshall 2018

Paratypes 7 **♂**, 7 **♀** QCAZI 260304–QCAZI 260310, QCAZI 260324–QCAZI 260330. Label 1: ECU, Napo, Tena 12 km SW 500 m, dung trap 30–33 day 2–5, 8–11 Jul 1976. Label 2: Paratype *Bromeloecia undulata* Yau & Marshall 2018

***Bromeloecia wolverinei* Yau & Marshall, 2018**

Holotype **♂** QCAZI 260101. Label 1: ECU, Galapagos, St. Cruz, 2 km N Bellavista 360 m, guavathicket, 14. v–13 vii. 85 FIT Agriarea, S. & J. Peck. Label 2: Holotype, *Bromeloecia wolverinei* Yau & Marshall 2018

Paratypes 2 **♂** QCAZI260102 and QCAZI 260103. Label 1: same data as the Holotype. Label 2: Paratype, *Bromeloecia wolverinei* Yau & Marshall 2018

Paratypes 10 **♂**, 13 **♀** QCAZI 260104–QCAZI 260112, QCAZI 260120, QCAZI 260127–QCAZI 260139. Label 1: ECU, Galap. St. Cruz, Los Gemelos, 600 m, Scalesia for, dung tp., 31. I–4. II. 1989 B. J. Sinclair. Label 2: Paratype, *Bromeloecia wolverinei* Yau & Marshall 2018

***Coproica bispatha* Bergeron, Marshall & Swann, 2015**

Paratypes QCAZI 2398–QCAZI 2404, QCAZI 2422–QCAZI 2430, QCAZI 2436–QCAZI 2438, QCAZI 2456–QCAZI 2467. Label 1: Ecu: Galap: St. Cruz, 2 km N Bellavista, 360 m, guava thicket, 2. vii. 85, S & J. Peck, Agricultural area, FIT, and 14. v–13. vii. 1985. Label 2: PARATYPE, *Coproica bispatha* M. D. Bergeron

Paratypes QCAZI 2405–QCAZI 2421, QCAZI 2431–QCAZI 2435, QCAZI 2441–QCAZI 2450. Label 1: Ecu: Galap: St. Cruz 4 km N Bellavista, Media Luna 620 m, 21–31. V. 85, S & J. Peck, Miconia zone, dung trap, and 14. v–13. vii. 85. Label 2: PARATYPE, *Coproica bispatha* M. D. Bergeron

Paratypes QCAZI 2455. Label 1: Ecu: Galap: Isabela 7 km WNW Sto. Tomas near Campamento Pumas, 640 m, 8. vii. 1985, sifted horse dung, S. & J. Peck. Label 2: PARATYPE, *Coproica bispatha* M. D. Bergeron

Paratypes QCAZI 2452–QCAZI 2454. Label 1: Ecu: Galap. St. Cruz 25 km N Bellavista; 360 m, 13. vii. 1985, guava thicket Agricultural area, S. & J. Peck. Label 2: PARATYPE, *Coproica bispatha* M. D. Bergeron

Paratypes QCAZI 2439, QCAZI 2440, QCAZI 2451. Label 1: Ecu: Galap: St. Cruz, Los Gemelos, 31 km n Sta. Ros. Scalesia forest 570 m, 15. vii. 1985, S. & J. Peck. Label 2: PARATYPE, *Coproica bispatha* M. D. Bergeron

***Coproica brachystyla* Bergeron, Marshall & Swann, 2015**

Paratype QCAZI 2390. Label 1: Ecu: Napo, Jatun Sacha Res. 6 km E Misahuallí 450 m, 1°4' S, 77°37' W. Varzea dung pans, 2–7 May 2002, M. Buck, debu 00196107. Label 2: PARATYPE, *Coproica brachystyla*, M. D. Bergeron

Paratype QCAZI 2391. Label 1: Ecuador, Prov. Napo, Yasuni Res., forest malaise trap 3–20 Nov 1998, Pape & Viklund, debu 00116953. Label 2: PARATYPE, *Coproica brachystyla,* M. D. Bergeron

***Coproica diabolia* Bergeron, Marshall & Swann, 2015**

Paratype QCAZI 2392. Label 1: Ecu: Pichincha, Nanegalito, 7 km SE trout farm San José 1500 m, riverine forest, sweep tree falls, 27–30 Oct 1999, S. A. Marshall Label 2: PARATYPE *Coproica diabolia* M.D. Bergeron

Paratype QCAZI 2393. Label 1: Ecu: Napo, Prov. 5 km N El Chaco, 15–II–1983, M. J. Sharkey, Malaise trap & wet det. Label 2: PARATYPE *Coproica diabolia* M. D. Bergeron

Paratype QCAZI 2394. Label 1: Ecu: Pich. 47 km S Sto. Domingo, Río Palenque Res. Stn., 2. V. 1987, B. Brown, light. Label 2: PARATYPE *Coproica diabolia* M. D. Bergeron

Paratype QCAZI 2395. Label 1: ECU: Pichincha, Maquipucuna Biol. Res. 1200 m, 0°7'34" N, 78°37'57" W, on foliage, 27–28 Apr 2002, M. Buck. Label 2: PARATYPE *Coproica diabolia* M. D. Bergeron.

***Coproica galapagosensis* Bergeron, Marshall & Swann, 2015**

Paratype QCAZI 2377–QCAZI 2389 and QCAZI 2376. Label 1: Ecu: Galap: Espanola, Bahia Manzanilla, 7–10. VI. 1985, S. Peck, J. Peck, sand beach, carrion trap. Label 2: PARATYPE, *Coproica galapagos* M. D. Bergeron

***Coproica novacula* Bergeron, Marshall & Swann, 2015**

Paratype QCAZI 2396. Label 1: ECU: 12–23 III. 1992, S. Peck. Label 2: PARATYPE, *Coproica novacula* M. D. Bergeron

***Leptocera papallacta* Buck & Marshall, 2009**

Holotype **♂** QCAZI 2081. Label 1: ECU: Pichincha, Cotopaxi Natl. Pk. Quebrada Mishahuaicu, 3600 m, pans along stream, 26 Oct–8 Nov 1999, S. A. Marshall. Label 2: HOLOTYPE *Leptocera papallacta* Buck sp. n.

Paratypes 31 **♂**, 20 **♀** QCAZI 2050–QCAZI 2080 and QCAZI 2082–QCAZI 2101. Label 1: same data as the Holotype. Label 2: PARATYPE, *Leptocera papallacta* Buck sp. n.

***Leptocera plax* Buck & Marshall, 2009**

Paratype **♂** QCAZI 2102. Label 1: ECU: Napo, El Chaco, 4.8 km W, 1750 m, 7 Nov 1999, S. A. Marshall. Label 2: *Leptocera plax* Buck sp. n. Det. Buck 2008. Label 3: PARATYPE, *Leptocera plax* Buck sp. n.

Paratypes 3 **♂** QCAZI 2103, QCAZI 2107 and QCAZI 2108. Label 1: ECU: Napo, 5 km N El Chaco, 15. II. 1983, M. J. Sharkey, malaise trap & Wet net. Label 2: *Leptocera plax* Buck sp. n. Det. Buck 2008. Label 3: PARATYPE, *Leptocera plax* Buck sp. n.

Paratype **♂** QCAZI 2104. Label 1: ECU: Napo Prov. El Chaco, 2000 m, 15–23. II. 1983, m. Mal.t p. L. Masner & M. Sharkey. Label 2: *Leptocera plax* Buck sp. n. Det. Buck 2008. Label 3: PARATYPE, *Leptocera plax* Buck sp. n.

Paratype **♂** QCAZI 2105. Label 1: ECU: Napo, Baeza, 1500 m, wet, montane rain forest, 16–19 May 1987, L. Coote & Brown. Label 2: *Leptocera plax* Buck sp. n. Det. Buck 2008. Label 3: PARATYPE, *Leptocera plax* Buck sp. n.

Paratype **♂** QCAZI 2106. Label 1: ECU: Napo, Cosanga 2.5 km W, pass, 5–7 Nov 1999, S. A. Marshall. Label 2: *Leptocera plax* Buck sp. n. Det. Buck 2008. Label 3: PARATYPE, *Leptocera plax* Buck sp. n.

***Minilimosina sclerophallus* Marshall, 1985**

Paratype QCAZI 1352. Label 1: ECU Pich. 9300´ 35 km E Tandapi, 24–29. vi. 1976, S. Peck. Label 2: PARATYPE *Minilimosina* (M.) *sclerophallus* Det. S. A. Marshall

***Photoantrops echinus* Kits & Marshall, 2013**

Holotype **♂** QCAZI 260066. Label 1: ECU, Napo, Sierra Azul Lodge 14 km W Cosanga 2200 m, 0°40'55S, 77°56'9 W at light, 8 May 2002, S. M. Paiero. Label 2: HOLOTYPE *Photoantrops echinus* Kits & Marshall 2013

***Poecilantrops stellans* Kits & Marshall, 2013**

Holotype **♂** QCAZI 259981. Label 1: ECU: Napo, Quito-Baeza pass, 1 km E 3950 m, forest edge, dung traps, 4–8 Nov 1999, S. A. Marshall. Label 2: HOLOTYPE *Poecilantrops stellans* Kits & Marshall 2012

Paratypes 6 **♂**, 8 **♀** QCAZI 259982, QCAZI 259983, QCAZI 259986, QCAZI 259988–QCAZI 259990, QCAZI 259992–QCAZI 259999. Label 1: Ecuador, Napo, Pichincha, Quito–Baeza pass, 4000 m, pans traps in moss, 4–8 Nov 1999, S. A. Marshall. Label 2: PARATYPE *Poecilantrops stellans* Kits & Marshall 2012

Paratypes 2 **♂**, 1 **♀** QCAZI 259984, QCAZI 259985, QCAZI 260000. Label 1: same data as the Holotype. Label 2: PARATYPE *Poecilantrops stellans* Kits & Marshall 2012

Paratypes **♂** QCAZI 259987. Label 1: ECU, Carchi, Bosque El Arrayán, 6 km E San Gabriel 2830 m, forest dung traps, 2–4 Nov 1999, S. A. Marshall. Label 2: PARATYPE *Poecilantrops stellans* Kits & Marshall 2012

Paratype 1 **♀** QCAZI 259991. Label 1: ECU, Carchi, Páramo El Angel 18.8 km NW El Angel 3300 m, pan in Polylepis litter, 3 Nov 1999, S. A. Marshall. Label 2: PARATYPE *Poecilantrops stellans* Kits & Marshall 2012

**FAMILY SYRINGOGASTRIDAE**

***Syringogaster atricalyx* Marshall & Buck, 2009**

Paratypes MEPN 36943 and MEPN 36944. Label 1: ECUADOR: Dpto. Orellana Reserva Waorani, 1 km S. Onkone Gare Camp, Transect Ent. 10 OCT 1994, 00°39'25.7" S, 76°27'10.8" W, 216.3 m, T. L. Erwin et al., Trans. 4, Sta. 10, Fogging in terre firme forest Lot # 949 and Sta. 3, Lot # 1553. PARATYPE, *Syringogaster atricalyx* Marshall

***Syringogaster brachypecta* Marshall & Buck, 2009**

Holotype QCAZI 2130. Label 1: ECU: Napo Jatun Sacha Res. 6 km E Misahualli 450 m, 01°4' S, 77°37' W, varzea, 30 Apr–8 May 2002, S. A. Marshall, debu 00196252. Label 2: HOLOTYPE, *Syringogaster brachypecta* Marshall

Paratype QCAZI 2131. Label 1: Ecu: Napo Jatun Sacha Res. 6 km E Misahualli, 450 m, 01°4' S, 77°37' W, on foliage, 30 Apr–8 May 2002, M. Buck. Label 2: PARATYPE, *Syringogaster brachypecta* Marshall

***Syringogaster plesioterga* Marshall & Buck, 2009**

Paratype MEPN 36946. Label 1: ECUADOR: Dpto Orellana Reserva Waorani, 1 km S. Onkone Gare Camp, Transect Ent. 31 JUL 1994, 00°39'25.7" S, 76°27'10.8" W, 216.3 m, T. L. Erwin et al., Trans. 3, Sta. 7, Fogging in terre firme forest Lot # 766. Label 2: PARATYPE, *Syringogaster plesioterga* Marshall

Paratypes MEPN 36947, MEPN 36948 and MEPN 36949. Label 1: ECUADOR. Dpto Orellana: nr Yasuni National Park, Tiputini Biodiversity Station, 8 FEB 1999, (6 FEB 1999 and 5 FEB 1999), 00°37'55" S, 76°08'39" W, 220–250 m, T. L. Erwin et al., Trans. 4, 7 and 9 Sta. 3, 6 and 5 Fogging in terre firme forest Lot # 2032, Lot # 2065, Lot # 2087. Label 2: PARATYPE, *Syringogaster plesioterga* Marshall

**FAMILY TACHINIDAE**

***Erythromelana arciforceps* Inclan, 2013**

Paratype QCAZI 2638. Label 1: Nova Teutonia, S. C. Brazil, Nov 1970, F. Plaumann. Label 2: PARATYPE, *Erythromelana arciforceps*, Inclan D. J.

***Erythromelana catarina* Inclan, 2013**

Paratype QCAZI 2637. Label 1: Nova Teutonia, S. C. Brazil, Nov 1970, F. Plaumann. Label 2: PARATYPE, *Erythromelana catarina*, Inclan D. J.

***Erythromelana cryptica* Inclan, 2013**

Paratype QCAZI 2636. Label 1: Ecuador, Napo, 7 km S. Baeza, 22. II. 79, 2000 m, G. Wood, M. Wood. Label 2: PARATYPE, *Erythromelana cryptica* Inclan D. J.

***Erythromelana distincta* Inclan, 2013**

Paratypes QCAZI 2641 and QCAZI 2642. Label 1: Nova Teutonia, S. C. Brazil, Ago 1969, F. Plaumann, and DEC. 1970. Label 2: PARATYPE, *Erythromelana distincta*, Inclan D. J.

***Erythromelana leptoforceps* Inclan, 2013**

Paratype QCAZI 2634 and QCAZI 2635. Label 1: Nova Teutonia, S. C. Brazil, Oct 1961 and 1970, F. Plaumann. Label 2: PARATYPE, *Erythromelana leptoforceps* Inclan D. J.

***Erythromelana woodi* Inclan, 2013**

Paratype QCAZI 2640. Label 1: Bolivia, Cbba Chapare, Villa Tunarí–Cochabamba road km 388, 2200 m, G. & M. Wood, 3. XII. 96. Label 2: PARATYPE, *Erythromelana woodi*, Inclan D. J.

**FAMILY TANIPEZIDAE**

***Neotanypeza marshalli* Lonsdale, 2013**

Holotype QCAZI 2471. Label 1: ECU: Napo, Sierra Azul Res. 14 km W Cosanga, 2200 m, 0°40'55" S, 77°56'9" W, 8–11 May. 2002, S. A. Marshall. Label 2: HOLOTYPE, *Neotanypeza marshalli* Lonsdale

Paratypes 4 QCAZI 2472–QCAZI 2475. Label 1: same data as the Holotype. Label 2: PARATYPE, *Neotanypeza marshalli* Lonsdale

***Neotanypeza plotoplax* Lonsdale, 2013**

Holotype **♂** QCAZI 2469. Label 1: Ecu: Prov. Pichincha, Bellavista Reserve trail B 2200 m, 30 Oct 1999, S. A. Marshall. Label 2: HOLOTYPE, *Neotanypeza plotoplax* Lonsdale

***Neotanypeza posthos* Lonsdale, 2013**

Paratype QCAZI 2470. Label 1: Ecu; Napo, Baeza 2000 m, 1/3/1979, S. A. Marshall. Label 2: PARATYPE, *Neotanypeza posthos* Lonsdale

***Neotanypeza vexilla* Lonsdale, 2013**

Holotype QCAZI 2468. Label 1: ECU: Napo, Sierra Azul Res. 14 km W Cosanga 2200 m, 0°40'55" S, 77°56'9" W, 8–11 May 2002, S. A. Marshall. Label 2: HOLOTYPE, *Neotanypeza vexilla* Lonsdale

**FAMILY TEPHRITIDAE**

***Anastrepha amaryllis* Tigrero, 1998**

Holotype QCAZI 3442. Label 1: Ecuador, Napo, Coca, X–87, Legit G. Onore. Label 2: *Anastrepha amaryllis,* Tipo, Det. J. Tigrero

***Anastrepha anopla* Norrbom & Korytkowski, 2012**

Holotype MEPN 37358. Label 1: ECUADOR: NAPO, Res. Ethnica Waorani, 1 km S. Onkone Gare Camp. Trans. Ent. 6 Oct 1994, 220 m, 00°39'10'' S, 076°26' W. T. L. Label 2: Insecticidal fogging of mostly bare green leaves, some with covering of lichenous or bryophytic plants in terre firme forest. At Trans 10. Sta. 6 Project MAXUS Lot 885. Label 3: HOLOTYPE, *Anastrepha anopla* N + K

Paratype MEPN 37359. Label 1: same data as the Holotype, except: 10 feb 1995. Label 2: same data as the Holotype, except: At Trans 7. Sta. 5 Project MAXUS Lot 994. Label 3: PARATYPE, *Anastrepha anopla* N + K

***Anastrepha grandicanina* Norrbom & Korytkowski, 2012**

Paratype MEPN 37360. Label 1: ECUADOR: NAPO, Res. Ethnica Waorani, 1 km S. Onkone Gare Camp. Trans. Ent. 22 jun 1996, 220 m, 00°39'10'' S, 076°26' W. T. L. Erwin, et al. collectors. Label 2: Insecticidal fogging of mostly bare green leaves, some with covering of lichenous or bryophytic plants in terre firme forest. At Trans 6. Sta. 3 Project MAXUS Lot 1573. Label 3: PARATYPE, *Anastrepha grandicanina*

***Anastrepha hadracantha* Norrbom & Korytkowski, 2012**

Holotype MEPN 37361. Label 1: ECUADOR: NAPO, Res. Ethnica Waorani, 1 km S. Onkone Gare Camp. Trans. Ent. 12 feb 1995, 220 m, 00°39'10'' S, 076°26' W. T. L. Erwin, et al. Label 2: Insecticidal fogging of mostly bare green leaves, some with covering of lichenous or bryophytic plants in terre firme forest. At Trans 6. Sta. 4 Project MAXUS Lot 1033. Label 3: HOLOTYPE, *Anastrepha hadracantha* N + K

Paratype MEPN 37362. Label 1: same data as the Holotype, except: 21 jun 1994. Label 2: same data as the Holotype, except: At Trans 9 x–trans. 82 m, Project MAXUS Lot 717. Label 3: PARATYPE, *Anastrepha hadracantha* Norrborn & Korytkowski

***Anastrepha haplacantha* Norrbom & Korytkowski, 2012**

Holotype MEPN 37363. Label 1: ECUADOR: NAPO, Res. Ethnica Waorani, 1 km S. Onkone Gare Camp. Trans. Ent. 9 feb 1995, 220 m, 00°39'10'' S, 076°26' W. T. L. Erwin, et al. Label 2: Insecticidal fogging of mostly bare green leaves, some with covering of lichenous or bryophytic plants in terre firme forest. At Trans 1. Sta. 10 Project MAXUS Lot 979. Label 3: HOLOTYPE, *Anastrepha haplacantha* N + K

***Anastrepha hyperacantha* Norrbom & Korytkowski, 2012**

Holotype MEPN 37364. Label 1: ECUADOR: NAPO, Res. Ethnica Waorani, 1 km S. Onkone Gare Camp. Trans. Ent. 5 feb 1996, 220 m, 00°39'10'' S, 076°26' W. T. L. Erwin, et al. Label 2: Insecticidal fogging of mostly bare green leaves, some with covering of lichenous or bryophytic plants in terre firme forest. At Trans 3. Sta. 1 Project MAXUS Lot 1421. Label 3: HOLOTYPE, *Anastrepha hyperacantha* N + K

Paratype MEPN 37365. Label 1: same data as the Holotype, except: 9 Oct 1994. Label 2: same data as the Holotype, except: At Trans 6. Sta. 8 Project MAXUS Lot 927. Label 3: PARATYPE, *Anastrepha hyperacantha* Norrborn & Korytkowski

***Anastrepha isolata* Norrbom & Korytkowski, 2009**

Holotype MEPN 37372. Label 1: ECUADOR: NAPO, Res. Ethnica Waorani, 1 km S. Onkone Gare Camp. Trans. Ent. 10 feb 1996, 220 m, 00°39'10'' S, 076°26' W. T. L. Erwin, et al. Label 2: Insecticidal fogging of mostly bare green leaves, some with covering of lichenous or bryophytic plants in terre firme forest. At Trans 9. Sta. 3 Project MAXUS Lot 1483. Label 3: HOLOTYPE, *Anastrepha isolata* N + K

***Anastrepha macracantha* Norrbom & Korytkowski, 2012**

Holotype MEPN 37366. Label 1: ECUADOR: NAPO, Res. Ethnica Waorani, 1 km S. Onkone Gare Camp. Trans. Ent. 6 Jul 1995, 220 m, 00°39'10'' S, 076°26' W. T. L. Erwin, et. al. Label 2: Insecticidal fogging of mostly bare green leaves, some with covering of lichenous or bryophytic plants in terre firme forest. At Trans 10. Sta. 4 Project MAXUS Lot 1124. Label 3: HOLOTYPE, *Anastrepha macracantha* N + K

***Anastrepha neogigantea* Norrbom & Korytkowski, 2012**

Holotype MEPN 37371. Label 1: ECUADOR: NAPO, Res. Ethnica Waorani, 1 km S. Onkone Gare Camp. Trans. Ent. 6 Jul 1995, 220 m, 00°39'10'' S, 076°26' W. T. L. Erwin, et. al. Label 2: Insecticidal fogging of mostly bare green leaves, some with covering of lichenous or bryophytic plants in terre firme forest. At Trans 10. Sta. 4 Project MAXUS Lot 1124. Label 3: HOLOTYPE, *Anastrepha neogigantea*

***Molynocoelia erwini* Norrbom, 2011**

Holotype MEPN 38220. Label 1: ECUADOR: Orellana: Reserva Etnica Waorani Onkone Gare Camp, Transect 4, 0°39'25.7" S, 76°27'10.8" W, 216.3 m, Sheet 7. Label 2: Insecticidal fogging, terre firme forest, 15 Oct 2005, T. L. Erwin, Pimienta et al. Project Maxus lot 3036. Label 3: HOLOTYPE, *Molynocoelia erwini* Norrborn

**ORDER EPHEMEROPTERA**

**FAMILY LEPTOPHLEBIIDAE**

***Atopophlebia pitculya* Flowers, 2012**

Holotype QCAZI 2743. Label 1: Ec. Manabí, Est. Jurón, 26–feb–2010, R. W. Flowers. Label 2: Holotype

Alotype QCAZI 2744 and Paratype 2745. Label 1: same data as the Holotype. Label 2: Allotype

Paratype QCAZI 2746. Label 1: vía Bilsa–Naranjal, 6–mar–2010, R. W. Flowers. Label 2: Paratype

Paratype QCAZI 2747. Label 1: Ec. Manabí, Est. Jurón, 25-ene-2010, F. García, P. García, R. W. Flowers. Label 2: Paratype

**ORDER HEMIPTERA**

**FAMILY COREIDAE**

***Onoremia acuminata* Brailovsky, 1995**

Holotype 1409. Label 1: Ecuador, Napo Tondaci, MAR 1991, Gonore. Label 2: HOLOTYPE. Label 3: H. Brailovsky A. det. *Onoremia acuminata* Brailovsky

**ORDER HYMENOPTERA**

**FAMILY APIDAE**

***Oxytrigona huaoranii* González & Roubik, 2008**

Paratypes 20 QCAZI 1578–QCAZI 1597. Label 1: Ecuador, Napo Prov. Yasuni Nat. Park. Est. La Catolica, 14–26 Apr 1998 and 13–27 Apr 1998. D. Roubik. Label 2: Paratype *Oxytrigona huaoranii* 2006 González & Roubik n. sp.

**FAMILY DIAPRIIDAE**

***Turripria woldai* Masner & García, 2002**

Paratype QCAZI 1598. Label 1: Panama, C. Z., Barro Colorado Is., 2–8. VII. 1978, Light trap I. H. Wolda. Label 2: Paratype *Turripia woldai* Masner & García CNC N° 22462. Label 3: *Turripia woldai* Det. L. Masner 2002

**FAMILY DRYINIDAE**

***Gonatopus sandovalae* Guglielmino, Olmi & Speranza, 2016**

Holotype QCAZI 200255. Label 1: Ecuador, Pichincha, C. C. Río Palenque, COL. MAB–UNESCO 10 JAN 1981 S. Sandoval. Label 2: HOLOTYPE, *Gonatopus sandovalae* 2015, M. Olmi

***Gonatopus tapiai* Olmi & Guglielmino, 2016**

Holotype QCAZI 200256. Label 1: Ecuador, Cotopaxi, Las Pampas, 1800 m, 78°57'04" W; 00°25'16" S, 30 JUN 1997, I. G. Tapia, P. Ponce. Label 2: HOLOTYPE, *Gonatopus tapiai* n. sp. 2015, M. Olmi

**FAMILY ENCYRTIDAE**

***Anagyrus lizanorum* Noyes & Menezes, 2000**

Paratype MEPN 37250. Label 1: COSTA RICA: San José Cerro de la Muerte 19 km, 5–3 W Empalme 2600 m, IV–V–1993 P. Manson. Label 2: PARATYPE, *Anagyrus lizanorum* sp. n. det J. S. Noyes 2000 + A. Menezes MEPN 37250

Paratype MEPN 37261. Label 1: COSTA RICA: San Jose Cerro de la Muerte 26 km N. San Isidro. Label 2: 9°30' N 83°43' W ix. x. 1992, 2100 m. P. Hanson. Label 3: PARATYPE, *Anagyrus lizanorum* sp. n det J. S. Noyes 2000 + A. Menezes

Paratype MEPN 37262. Label 1: COSTA RICA: San Jose Cerro Muerte, 19 km W. Empalme. Label 2: 2600 m, 1991, P. Hanson, C. Godoy. Label 3: PARATYPE, *Anagyrus lizanorum* sp. n. det J. S. Noyes 2000 + A. Menezes

***Anagyrus paralia* Noyes & Menezes, 2000**

Paratype MEPN 37259, MEPN 37260. Label 1: COSTA RICA: San Jose 26 km. N. San Isidro 2100 m. Label 2: 9°30' N, 83°43' W 11. iv. 1993 P. Hanson and i–ii. 1991, C. Godoy. Label 3: PARATYPE, *Anagyrus paralia* sp. n. det J. S. Noyes 2000 + A. Menezes

***Anagyrus sinope* Noyes & Menezes, 2000**

Paratype MEPN 37256. Label 1: CIBC, Trinidad org. USA: Texas Meame X–1978. Label 2: Lab reared *Phenococcus grenadensis*. Label 3: PARATYPE, *Anagyrus sinope* sp. n. det J. S. Noyes 2000 + A. Menezes

Paratype MEPN 37257. Label 1: USA: Texas Heame ix–1978 F. D. Bennett. Label 2: ex *Phenococcis gossypii*. Label 3: PARATYPE, *Anagyrus sinope* sp. n. det J. S. Noyes 2000 + A. Menezes

Paratype MEPN 37258. Label 1: BAHAMAS: San Andros viii. 1977 F. D. Bennett. Label 2: ex *Phenococcis* on Acalypha. Label 3: PARATYPE, *Anagyrus sinope* sp. n. det J. S. Noyes 2000 + A. Menezes

***Blepyrus hansoni* Noyes, 2000**

Paratypes MEPN 37247 and MEPN 37248. Label 1: COSTA RICA: Guanascaste Santa Rosa NP D. Janzen, I. Gauld SE 6C and H3O, 21. ii–14. iii. 1987. Label 2: PARATYPE, *Blepyrus hansoni* sp. n det J. S. Noyes 2000

***Blepyrus zenonis* Noyes, 2000**

Paratype MEPN 37249. Label 1: COSTA RICA: Guanascaste Santa Rosa NP BH90, iii. 1987, D. Janzen, I. D. Gauld. Label 2: PARATYPE, *Blepyrus zenonis* sp. n det J. S. Noyes 2000

***Gyranusoidea amasis* Noyes, 2000**

Paratype MEPN 37255. Label 1: COSTA RICA: Guanascaste PV Sta. Rosa NP Hacienda–3–O. Label 2: Janzen & Gauld, 21. ii.–14. iii. 87 PARATYPE, *Gyranusoidea amasis* sp. n det J. S. Noyes 2000

***Gyranusoidea rhodope* Noyes, 2000**

Paratypes MEPN 37253 and MEPN 37254. Label 1: COSTA RICA: Guanascaste PV Sta. Rosa NP Sn. Emilio–8–C and Emilio–6–C. Label 2: Janzen & Gauld 14. iii.–4. iv. 87 and 21. ii.–14. iii. 87. Label 3: PARATYPE, *Gyranusoidea rhodope* sp. n det J. S. Noyes 2000

***Hambletonia pilosifrons* Sharkov & Woolley, 1997**

Paratype MEPN 37251. Label 1: COSTA RICA: Guanascaste PV. Sta. Rosa NP Hacienda–1–O. Label 2: Janzen & Gauld 20. xii. 86–10. i. 87. Label 3: PARATYPE, *Hambletonia pilosifrons* Sharkov & Woolley

Paratype MEPN 37252. Label 1: COSTA RICA: Heredia 3 km S. Puerto Viejo. Label 2: OTS–La Selva, 100 m, ii–iii 1993, P. Hanson. Label 3: PARATYPE, *Hambletonia pilosifrons* Sharkov & Woolley

**FAMILY FORMICIDAE**

***Basiceros onorei* Baroni & De Andrade, 2007**

Holotype QCAZI 1334. Label 1: Ecuador, Baños de Agua Santa, Prov. Tungurahua 01°24' S, 78°25' W, 1860 m, 26. 8. 2004, Juan Manuel Vieira Correa. Label 2: *Basiceros onorei* Holotype Baroni Urbani & de Andrade 2007, Det. C. Baroni Urbani

***Leptanilloides copalinga* Delsine & Donoso, 2015**

Holotype QCAZI 254329. Label 1: *Leptanilloides copalinga*, HOLOTYPE, Delsine & Donoso 2015. Ecuador, Worker, Zamora Chinchipe Prov., Copalinga Private Reserve 1510 m, 4°4'56,6" S, 78°58'5,71" W, 2 Apr 2010, soil sample, coll Thibaut Delsine and Tania Milena Arias-Penna

Comments: Collecting data obtained from the publication, information was not on the label

***Leptanilloides prometea* Delsine & Donoso, 2015**

Holotype QCAZI 25438. Label 1: *Leptanilloides prometea*, HOLOTYPE, Delsine & Donoso 2015. Ecuador, Worker, Zamora Chinchipe Prov., Reserva Biológica San Francisco (RBSF), 2010 m, 3°58' S, 79°05' W, 13 May 2010, within 0,5 m2 of leaf litter extracted with a mini–Winkler apparatus for and 96 h., coll. Thibaut Delsine and Tania Milena Arias–Penna

Comments: Collecting data obtained from the publication, information was not on the label

Paratype QCAZI 254326–QCAZI 254327. Label 1: *Leptanilloides prometea*, PARATYPE, Delsine & Donoso 2015. same data as the Holotype and mini–Winkler apparatus for 48 h., coll.

Comments: Collecting data obtained from the publication, information was not on the label

***Pachycondyla cernua* Mackay & Mackay, 2010**

Paratype QCAZI 2169. Label 1: Ecuador, Napo, near Dureno, 287 m, 20–vii–2005, W & E Mackay # 21270. Label 2: 0°4'40.8" N; 76°43'50.5" W, forest, day sol, last in log. brood in nest. Label 3: PARATYPE *Pachycondyla cernua*, Mackay and Mackay. Desg. Mackays 2005

***Pyramica heterodonta* Rigato & Scupola, 2008**

Paratype QCAZI 2157 and QCAZI 2158. Label 1: Ecuador, Pichincha, Pasochoa, 2900 m, 0°25'19" S–78°30'57" W, 26. vii. 2006 leg. G. Caoduro & A. Scupola. Label 2: *Pyramica heterodonta* Rigato & Scupola 2008 PARATYPUS

***Simopelta transversa* Mackay & Mackay, 2008**

Paratype QCAZI 2168. Label 1: Pance, 1700 m, Mun de Cali, 16–VI. Mt. rain forest. Label 2: Colombia, Valle, 1971, W. L. Brown, S. Chaplin. Label 3: PARATYPE *Simopelta transversa* Mackay & Mackay, Desg. Mackays 2005

***Strumigenys lojaensis* Lattke & Aguirre, 2015**

Holotype QCAZI 3078. Label 1: ECU, Loja, 6 km SSE Loja, Reserva Madrigal, 2350 m, 4.04655°S, 79.17583° W, bosque secundario, rastrojo. Label 2: 28. VIII. 2014, G. Piedra, M. Velez, C. Gomez, M. Tuza, JE. Lattke 3590–14 muestra hojarasca tamizada. Label 3: HOLOTYPE *Strumigenys lojanensis* n. sp. Lattke 2014

***Strumigenys longimala* Baroni & de Andrade, 2007**

Holotype QCAZI 1332. Label 1: Ecuador, Yasuní Scientific Station, Prov. Napo, 28. 8. 2004, leaf–litter C. Baroni Urbani & M. L. de Andrade. Label 2: *Strumigenys longimala* Holotype, Baroni Urbani & de Andrade. Det. C. Baroni Urbani

Paratypes QCAZI 1331 and QCAZI 1333. Label 1: same data as the Holotype. Label 2: *Strumigenys longimala* Paratype, Baroni Urbani & de Andrade, Det. C. Baroni Urbani

***Strumigenys nageli* Baroni & de Andrade, 2007**

Holotype QCAZI 1330. Label 1: Ecuador, Rio Verde, Prov. Esmeraldas, Vía San Lorenzo km 67, 22. 8. 2004, leaf–litter C. Baroni Urbani & M. L. de Andrade. Label 2: *Strumigenys nageli* Holotype, Baroni Urbani & de Andrade. Det. C. Baroni Urbani

Paratypes QCAZI 1328 and QCAZI 1329. Label 1: same data as the Holotype. Label 2: *Strumigenys nageli* Paratype, Baroni Urbani & de Andrade. Det. C. Baroni Urbani

***Strumigenys onorei* Baroni & De Andrade, 2007**

Holotype QCAZI 1335. Label 1: Ecuador, Baños de Agua Santa, Prov. Tungurahua 01°24' S, 78°25' W, 1860 m, 26. 8. 2004, Juan Manuel Vieira Correa. Label 2: *Strumigenys onorei* Holotype Baroni & De Andrade 2007. Det. C. Baroni Urbani

Paratype QCAZI 1336. Label 1: same data as the Holotype. Label 2*: Strumigenys onorei* Paratype Baroni & De Andrade 2007. Det. C. Baroni Urbani

**FAMILY HALICTIDAE**

***Chlerogella euprepia* Engel, 2010**

Paratype QCAZI 2048. Label 1: Ecuador, Napo, Vía Hollin–Loreto km 25, 1100 m, 6/12/87, Lg. M. M. Mena. Label 2: PARATYPE *Chlerogella euprepia* Michael S. Engel

***Chlerogella mourella* Engel, 2003**

Paratype QCAZI 2049. Label 1: Ecuador, Napo, Cosanga, Aragon, 8 Nov 1993, G. Onore. Label 2: PARATYPE *Chelrogella mourella* Michael S. Engel

**FAMILY SPHECIDAE**

***Pison arachniraptor* Menke, 1988**

Paratype QCAZI 1973. Label 1: Ecuador, Napo, Muyuna 5 Km W of Tena, 22–iv–1981, M. Cooper. Label 2: PARATYPE *Pison arachniraptor* A. S. Menke

**FAMILY TRICHOGRAMMATIDAE**

***Adryas erwini* Pinto & Owen, 2004**

Holotype MEPN 5424. Label 1: ECUADOR: NAPO, Tiputini Biodiversity Sta. nr. Yasuni Nat'l Park x–22–1998; 220–250 m. 00°37'55" S, 76°8'39" W, fogging terre firme forest, T. Erwin, et al. Lot # 1971, Key: 98–10–22–11. Label 2: HOLOTYPE, *Adryas erwini* Pinto + Owen

Allotype MEPN 9947. Label 1: ECUADOR: NAPO, Onkone Gare Camp 1 km. S.; Reserva Etnica Waorani; 216.3 m. x–5–1995, 00°39'25.7" S, 76°27'10.8" W, T. Erwin, et al.; Lot 1194, Key: 95-10–05–13. Label 2: ALLOTYPE, *Adryas erwini* Pinto + Owen

Comments: specimens preserved in glass slides

***Pachamama speciosa* Owen & Pinto, 2004**

Holotype MEPN 5405. Label 1: ECUADOR: NAPO, Tiputini Biodiversity Sta. nr. Yasuni Nat'l Park x–26–1998; 220–250 m. 00°37'55" S, 76°8'39" W fogging terre firme forest, T. Erwin, et. al. Lot # 1940, Key: 98–10–26–17. Label 2: HOLOTYPE, *Pachamama speciosa* Owen + Pinto

**ORDER LEPIDOPTERA**

**FAMILY SATURNIIDAE**

***Automeris abdominapoensis* Brechlin & Meister, 2011**

Paratype QCAZI 2731. Label 1: Ecuador, Napo, Cosanga, Las caucheras, Yanayacu research station 00°35,96S, 077°53,43 W, 2150 m, 4. 04. 09, Leg. H. Kaech. Label 2: Paratypus, *Automeris abdominapoensis* Brechlin & Meister 2011, Entomo–Santsphingia 4(1): 23

***Automeris abdomipichinchensis* Brechlin & Meister, 2011**

Paratype QCAZI 2732. Label 1: Intag, 27–II–8, 2400 m, Imbabura. Label 2: Paratypus, *Automeris abdomipichinchensis* Brechlin & Meister 2011, Entomo–Satsphingia 4(1): 23

***Automeris isabellae* Brechlin & Käch, 2017**

Paratypes QCAZI 224728 and QCAZI 224729. Label 1: Ecuador, Carchi, Limonal–Chical, road Carmen–Chical 00°53'36,4" N/78°13'39,5" W, 1075 m–1800 m, 25. 5. 12, leg. H. Kaech & F. Piñas. Label 2: Paratypus, *Automeris isabellae* Brechlin & Käch 2017 Entomo–Satsphingia 10(2): 70

***Automeris manzonoi* Brechlin, Käch & Meister, 2013**

Paratypes QCAZI 224725 and QCAZI 224726. Label 1: Ecuador, Manabí, El Balsamo, San Clemente, Road Bahía, dC–Jipijapa 0°45'09,9" S; 80°28'03,9" W, 142 m, 26. I. 12, leg. H. Käch & F. Piñas. Label 2: Paratypus, *Automeris manzanoi* Brechlin, Käch & Meister 2013. Entomo–Satsphingia 6(3): 9

***Automeris parapichinchaensis* Brechlin & Meister, 2011**

Paratype QCAZI 2726. Label 1: Ecuador, Pichincha, Pululahua crater, Niebli, road Quito–Calacalí 00°03,36,7' N, 78°30'27,1" W, 3088 m, 5. 10. 10, Leg. I. Manzano. Label 2: Paratypus, *Automeris parapichinchaensis* Brechlin & Meister 2011, Entomo–Satsphingia 4(1): 38

***Citheronia kaechi* Brechlin, 2019**

Paratype **♂** QCAZI 261235. Label 1: Ecuador oriente, Prov. Napo–Hollin Road Loreto–Coca 00°42.45.9' S, 77°44'26.7 W, 28. X. 2008, H. Kach. Label 2: Paratypus *Citheronia kaechi* Brechlin, 2019, Entomo–Satsphingia 12(2): 41–74

Paratype QCAZI 261236. Label 1: Ecuador oriente, Prov. Napo, Cascabeles, road Chaco–Reventador 00°09.55.08/77°45'25.7 W, 26. IX. 2013, H. Kach, I. Manzano. Label 2: Paratypus *Citheronia kaechi* Brechlin, 2019, Entomo–Satsphingia 12(2): 41–74

Paratypes 1 **♀** QCAZI 261237–QCAZI 261242. Label 1: Ecuador, Napo, Puente Río Pingullo, Road Hollìn-Loreto 00°44.18.7' S/77°33'13.6" W, 930 m, C. O. 26. 2. 14, leg. H. Kaech/I. Manzano. Label 2: Paratypus *Citheronia kaechi* Brechlin, 2019, Entomo–Satsphingia 12(2): 41–74

Paratype **♂** QCAZI 261238. Label 1: Ecuador, Napo, Los Cocodrilos km 36 Road Baeza–Tena 00°39.28.0' N 77°47'15.6 W, 1673 m, 10. V. 2013, H. Kach. Label 2: Paratypus *Citheronia kaechi* Brechlin, 2019, Entomo–Satsphingia 12(2): 41–74

Paratype QCAZI 261239 and QCAZI 261241. Label 1: Ecuador oriente, Prov. Napo–Narupa Road Loreto–Coca 0°43.30.47' S, 77°46'01.45 W 1100 m, 27. 04. 2017; H. Kaech. Label 2: Paratypus *Citheronia kaechi* Brechlin, 2019, Entomo–Satsphingia 12(2): 41–74

***Copaxa andorientalis* Brechlin & Meister, 2012**

Paratype QCAZI 2733. Label 1: Ecuador, Napo, Cosanga, Las caucheras, Yanayacu research station 00°35,96S, 077°53,43 W, 2150 m, 4–4–05, leg. H. Kaech. Label 2: Paratypus, *Copaxa andorientalis* Brechlin & Meister 2012, Entomo–Satsphingia 5(2): 12

Paratype QCAZI 2739. Label 1: Ecuador, Napo, Los Cocodrilos reserva, Road Baeza–Tena 00°38,25,8' N 77°48'20,0 W, 1898 m, 22. 8. 12, leg. H. Kach/I. Manzano. Label 2: Paratypus, *Copaxa andorientalis* Brechlin & Meister 2012, Entomo–Satsphingia 5(2): 12

***Copaxa litensis* Wolf & Colan, 2002**

Paratypes **♂**, **♀** QCAZI 1468 and QCAZI 1469. Label 1: Ecuador, Carchi Prov. Rd Salinas to San Lorenzo 7.4 km W Lita el. 800 m, 30 Apr 2000, GPS: 00°52.39N 78°29,53 W, W. K. Wolfe, S. Wolfe, C. Molan, M. Conlan. Label 2: *Copaxa litensis* Wolf & Colan 2001–(2002) Paratype **♂**

***Dirphia apeggyae* Brechlin, Meister & Käch, 2011**

Paratype 1 **♂** QCAZI 2729. Label 1: *Dirphia subhorca* male. 15/5/02, Los Bancos, Prov. Pichincha, Ecuador, H. Käch. Label 2: Paratypus, *Dirphia apeggyae* Brechlin & Meister, 2011, Entomo–Satsphingia 4(5): 14

***Dirphia sachai* Brechlin & Käch, 2017**

Paratype QCAZI 224727. Label 1: Ecuador, Oriente, Sucumbios, Bermejo Tecpetrol road Lumbaqui–Lago Agrio 0°12,02,3 N/77°18'52,2" W, 920 m, 10. 7. 17, leg. H. Käch. Label 2: Paratypus, *Dirphia sachai*, Brechlin & Käch 2017, Entomo–Satsphingia 10(2): 23

***Gamelia kaechi* Brechlin & Meister, 2012**

Paratype QCAZI 2727. Label 1: Ecuador, Napo, Cosanga, Las caucheras, Yanayacu research station 00°35,96S, 077°53,43 W, 2150 m, 28. 8. 6, Leg. H. Kaech. Label 2: Paratypus, *Gamelia kaechi* Brechlin & Meister 2012. Entomo–Satsphingia 5(1): 19

***Hirpida kaechi* Brechlin, 2019**

Paratypes QCAZI 261243–QCAZI 261245. Label 1: Ecuador, Napo, Los Cocodrilos/ reserva Sumaco Road Baeza–Tena 00°39.2015' S/ 77°47'29.9 O, 1800 m, 06. 08. 2018, H. Kaech, and H. Kaech/I. Manzano. Label 2: Paratypus, *Hirpida kaechi*, Brechlin 2019, Entomo–Satsphingia 12(1): 37

***Periga barragani* Brechlin, Käch & Meister, 2013**

Paratypes QCAZI 2728 and QCAZI 2738. Label 1: Ecuador, Carchi, Limonal–Chical, Road Carmen–Chical 00°50,30,2N, 78°13'39,3 W; 2335 m, 27. 6. 12; Leg. H. Kach. Label 2: Paratypus, *Periga barragani* Brechlin, Meister & Käch 2013. Entomo-Satsphingia 6(2): 26

Paratype QCAZI 2737. Label 1: Ecuador, Carchi, road El Limonal–Chical, 2150 m. Label 2: Paratypus, *Periga barragani* Brechlin, Meister & Käch 2013. Entomo–Satsphingia 6(2): 26

***Rothschildia aricia ariciopichichensis* Brechlin, Käch & Meister, 2012**

Paratype QCAZI 251886. Label 1: Ecuador, Pichincha, paseo del Quinde road Nono–Nanegalito 00°00'51,8" S/78°39'05,3" W, 1914 m, 25. II. II leg. H. Kaech. Label 2: PARATYPUS *Rothschildia aricia ariciopichichensis* Brechlin, Kach & Meister 2012, Entomo–Satsphingia xx. Noviembre 2012

***Rothschildia aricia napoecuadoriana* Brechlin & Meister 2010**

Paratype QCAZI 251884. Label 1: Ecuador, Morona Santiago, Antenas de Limón, Road Gualaceo–Mendez 3°00'04" S–78°30'49" W 2380 m, 5. 3. 08, leg, H. Kaech & I. Manzano. Label 2: PARATYPUS *Rothschildia aricia napoecuadoriana* Brechlin & Meister 2010, Entomo–Satsphingia 3(3): xx–xx, Junio 2010

Paratype QCAZI 251885. Label 1: Ecuador, Morona Santiago, km 68 Guamote–Macas 2°12'10,4" S–78°21'59,1" W, 2300 m, 13. 5. 10, Leg. H. Kaech & I. Manzano. Label 2: PARATYPUS *Rothschildia aricia napoecuadoriana* Brechlin & Meister 2010, Entomo–Satsphingia 3(3): xx–xx, Junio 2010

***Rothschildia inca incecuatoriana* Brechlin & Meister, 2012**

Paratype **♀** QCAZI 2735. Label 1: Ecuador, Napo, Cando, 1000 m, 25. 6. 2006. Label 2: Paratypus, *Rothschildia inca incecuatoriana*, Brechlin, Käch & Meister 2012, Entomo–Satsphingia 5(3): 17

***Rothschildia lebecuatoriana* Brechlin & Meister, 2012**

Paratypes QCAZI 2734, QCAZI 2740 and QCAZI 2742. Label 1: 28/12/02, Los Bancos, Prov. Pichincha, Ecuador. Label 2: Paratypus, *Rothschildia lebecuatoriana* Brechlin, Käch & Meister 2012, Entomo–Satsphingia 5(3): 15

Paratypes 1 **♂**, 1 **♀** QCAZI 251887 and QCAZI 251888. Label 1: 26/2/03 Los Bancos, Prov. Pichincha, Ecuador, H. Kach. Label 2: PARATYPUS *Rothschildia lebecuatoriana* Brechlin, Kach & Meister 2012, Entomo–Satsphingia 5(3): xx–xx Noviembre 2012

**ORDER ORTHOPTERA**

**FAMILY TETTIGONIIDAE**

***Artiotonus tinae* Montealegre, Morris, Sarria & Mason, 2011**

Holotype **♂** QCAZI 2801. Label 1: Ecuador, Prov. Pichincha, 7 km Sto. Domingo de los Colorados, Tinalandia 600 m, July 16, 1986, G. K. Morris. Label 2: *Artiotonus tinae* Montealegre et al. 2011. Holotype

Alotype **♀** QCAZI 2802. Label 1: same data as the Holotype. Label 2: *Artiotonus tinae* Montealegre et al. 2011. Alotipo. Specimen misplaced within the collection, not reviewed yet

***Supersonus undulus* Sarria, Morris, Windmill, Jackson & Montealegre, 2014**

Holotype **♂** QCAZI 3007. Label 1: Ecuador, Pichincha, Sto. Domingo de los Colorados, Tinalandia, Dec. 11–18, 2011, F. Sarria. Label 2: Type Label 3: *Supersonus undulus*

Allotype **♀** QCAZI 3008. Label 1: same data as the Holotype. Label 2: Type. Label 3: *Supersonus undulus*

**ORDER TRICHOPTERA**

**FAMILY ANOMALOPSYCHIDAE**

***Contulma paluguillensis* Holzenthal & Ríos, 2012**

Paratypes 1 **♀**, 2 ND QCAZI 2656–QCAZI 2658. Label 1: B. Rios–Touma & F. González, Ecuador, Pichincha, Res. Paluguillo, Quebrada Saltana 0°19'1,801" S, 78°13'8,8" W, 2848 m, 27 IV 2011. Label 2: PARATYPE *Contulma paluguillensis* Holzenthal & Ríos 2012

**PHYLUM MOLLUSCA**

**CLASS GASTROPODA**

**ORDER STYLOMMATOPHORA**

**FAMILY BULIMULIDAE**

***Bostryx bermudezae* Weyrauch, 1958**

Paratypes MEPN–INV 45854. Label 1: Bostryx bermudezae WEYRAUCH, C–PERU: Laraos, Valle Cañete, leg. Dativa Beltrán, Paratypes

Comments: 3 Paratypes preserved in the same box

***Bostryx vilchezi* Weyrauch, 1960**

Paratypes MEPN–INV 45867. Label 1: Bostryx vilchezi WEYRAUCH, N–PERU: Socota cerca Cutervo, 1950 m, leg. Weyrauch, Paratypes

Comments: 10 Paratypes preserved in the same box

***Sculatus versicolor lachayensis* Weyrauch, 1967**

Paratypes MEPN–INV 45919. Label 1: Bulimulus (Scutalus) versicolor lachayensis WEYRAUCH, C–PERU: Lomas de Lachay cerca Chancay, 300 m, leg. W. Weyrauch, Paratypes

Comments: 9 Paratypes preserved in the same box

**FAMILY CLAUSILIIDAE**

***Hemicena cerrateae* Weyrauch, 1958**

Paratypes MEPN–INV 45905. Label 1: Hemicena cerrateae WEYRAUCH, C–PERU: Ainín cerca Chiquián, 3300 m, leg. Weyrauch, Paratypes

Comments: 2 Paratypes preserved in the same box

***Parabalea omissa* (Weyrauch, 1957)**

Paratypes MEPN–INV 45903. Label 1: Temesa (Temesa) omissa WEYRAUCH, C–PERU: Tarmatambo cerca Tarma, 3400 m, leg. W. Weyrauch, Paratypes

Comments: 9 Paratypes preserved in the same box

***Steeriana celendinensis isidroensis* Weyrauch & Zilch, 1954**

Paratypes MEPN–INV 45898. Label 1: Steeriana celendinensis isidroënsis WEYRAUCH & ZILCH, N–PERU: Cerro San Isidro cerca Celendin, 2750 m–2800 m, leg. W. Weyrauch, Paratypes

Comments: 9 Paratypes preserved in the same box

**PHYLUM NEMATA**

**CLASS SECERNENTEA**

**ORDER STRONGYLIDA**

**FAMILY MOLONEIDAE**

***Neomolineus pierredesseti* Guerrero, 2020**

Paratype MEPN 7642. Label 1: PARATYPE. Label 2: En: *Caenolestes condorensis* N. Label 3: Cotacachi Cayapas Ecological Reserve, Imbabura, Ecuador (0°29'14" N, 78°29'33" W), 2600 m

**FAMILY STRONGYLOIDIDAE**

***Parastrongyloides noetropicalis* Guerrero, 2016**

Holotype MEPN 7748. Label 1: HOLOTYPE. Label 2: Bosque Protector Mindo–Nambillo, Cerro Guarumos, Pichincha, Ecuador, 9994339/17762119, 2600 m

Allotype MEPN 7749. Label 1: ALOTIPO. Label 2: same data as the Holotype

**PHYLUM PLATYHELMINTHES**

**CLASS CESTODA**

**ORDER PHYLLOBOTHRIIDEA**

**FAMILY PHYLLOBOTHRIIDAE**

***Clistobothrium amyae* Caira, Hayes & Jensen, 2020**

Holotype MEPN. Label 1: ex *Pseudocarcharias kamoharai* spiral intestine (2°12'24.4" S, 80°56'58.1" W) Santa Rosa de Salinas, Santa Elena, Ecuador, Coll: 23 May 2014. Label 2: *Clistobothrium amyae* Caira, Hayes & Jensen, HOLOTYPE

Paratype MEPN. Label 1: same data as the Holotype. Label 2: *Clistobothrium amyae* Caira, Hayes & Jensen, PARATYPE

Comments: specimens with no MEPN id numbers assigned, preserved in glass slides

***Clistobothrium gabywalterorum* Caira, Hayes & Jensen, 2020**

Holotype MEPN. Label 1: ex Pseudocarcharias kamoharai spiral intestine (2°12'24.4" S, 80°56'58.1" W) Santa Rosa de Salinas, Santa Elena, Ecuador, Coll: 23 May 2014. Label 2: *Clistobothrium gabywalterorum* Caira, Hayes & Jensen, HOLOTYPE

Comments: specimens with no MEPN id numbers assigned, preserved in glass slides

***Scyphophyllidium timvickiorum* Caira, Hayes & Jensen, 2020**

Holotype MEPN. Label 1: ex *Pseudocarcharias kamoharai* spiral intestine. Pacific Oc., Ecuador, Santa Elena, Santa Rosa de Salinas, (2°12'24.4" S, 80°56'58.1" W), Coll: 23 May 2014. Label 2: *Scyphophyllidium timvickiorum* Caira, Hayes & Jensen, HOLOTYPE

Paratype MEPN. Label 1: same data as the Holotype. Label 2: *Scyphophyllidium timvickiorum* Caira, Hayes & Jensen, PARATYPE

Comments: specimens with no MEPN id numbers assigned, preserved in glass slides

**ORDER TETRAPHYLLIDEA**

**FAMILY SERENDIPIDAE**

***Serendip deborahae* Brooks & Barriga, 1994**

Holotype MEPN and 2 specimens with the same label. Label 1: HOLOTYPE

Paratypes MEPN 4 specimens. Label 1: PARATYPE

“Puerto Bolivar, Provincia de el Oro, Ecuador”. Data extracted from the original paper

Comments: specimens with no MEPN id numbers assigned, preserved in glass slides
